# Solid/Gas *In Crystallo* Reactivity of an Ir(I) Methylidene Complex

**DOI:** 10.1021/acs.organomet.4c00119

**Published:** 2024-06-06

**Authors:** Kristof M. Altus, M. Arif Sajjad, Matthew R. Gyton, Adrian C. Whitwood, Samuel J. Page, Stuart A. Macgregor, Andrew S. Weller

**Affiliations:** † Department of Chemistry, University of York, Heslington, York YO10 5DD, U.K.; ‡ EaStCHEM School of Chemistry, North Haugh, University of St Andrews, St Andrews KY16 9ST, U.K.; § Department of Chemistry, University of Durham, Durham DH1 3LE, U.K.

## Abstract

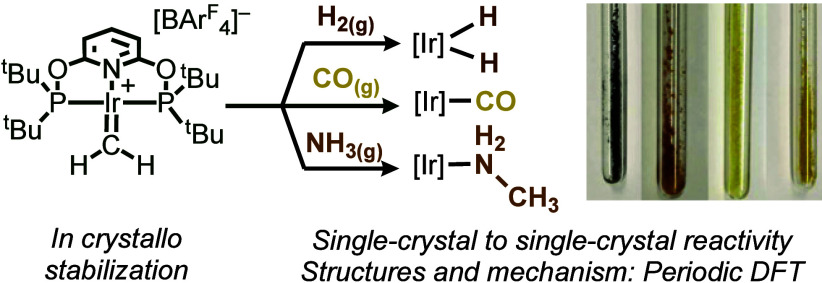

*In crystallo* stabilization of known, but solution unstable, methylidene complex [Ir(^t^Bu-PONOP)(=CH_2_)][BAr^F^_4_] allows single-crystal to single-crystal solid/gas reactivity associated with the {Ir=CH_2_} group to be studied. Addition of H_2_ results in [Ir(^t^Bu-PONOP)(H)_2_][BAr^F^_4_]; exposure to CO forms iridium(I) carbonyl [Ir(^t^Bu-PONOP)(CO)][BAr^F^_4_], and reaction with NH_3_ gas results in the formation of methylamine complex [(^t^Bu-PONOP)Ir(NH_2_Me)][BAr^F^_4_] via an aminocarbene intermediate. Periodic density functional theory and electronic structure analyses confirm the Ir=CH_2_ bond character but with a very low barrier to rotation around the Ir=CH_2_ bond. Calculations show that addition of NH_3_ to the electrophilic alkylidene carbon gives an initial ammonium ylid intermediate. Stepwise N–H and C–H transfers then form the aminocarbene intermediate as a kinetic product from which two successive C–H couplings lead to the more stable methylamine product.

Highly reactive organometallic complexes can be challenging to characterize in solution due to competitive reactivity with solvent,^1,2^ which may displace a weakly bound ligand or undergo activation at a metal center. Low temperatures are thus often used for characterization using *in situ* nuclear magnetic resonance (NMR) spectroscopy or recrystallization. An alternative approach is to remove the solvent completely, generating the reactive complex of interest directly in a single-crystal to single-crystal (SC–SC) transformation.^3^ For example, *in situ* low-temperature photocrystallography allows the characterization of highly reactive intermediates in SC–SC transformations using single-crystal X-ray diffraction.^4−6^ Similarly, SC–SC transformations can be used to synthesize cationic σ-alkane complexes, which are unstable in solution even at very low temperatures, by the solid/gas hydrogenation of room-temperature stable precursor alkene complexes.^7,8^ Interested in expanding the regions of chemical space in which such *in crystallo* solid-state molecular organometallic chemistry (SMOM)^9^ techniques can be used, we hypothesized that low-temperature solution synthesis and recrystallization could be combined with subsequent room-temperature SC–SC reactivity first to stabilize a solution-unstable organometallic complex *in crystallo* and then to explore its onward reactivity in solid/gas reactions. While conceptually straightforward, we believe this combined approach has not been previously adopted for molecular single-crystal reactivity. Related site-isolation of reactive metal fragments in MOFs is well-established.^10,11^

To demonstrate this methodology, the previously reported cationic methylidene complex [Ir(^t^Bu-PONOP)(=CH_2_)]^+^, **[1]**^**+**^, was chosen as an exemplar system, as shown in Figure 1 [^t^Bu-PONOP = κ^3^-2,6-(^t^Bu_2_PO)_2_C_6_H_3_N]. First reported by Carmona, Brookhart, and co-workers as the [B(C_6_F_5_)_4_]^−^ salt, **[1][B(C**_**6**_**F**_**5**_**)**_**4**_**]** is synthesized by a low-temperature (−20 °C, C_6_D_5_Br) hydride abstraction from the corresponding methyl complex Ir(^t^Bu-PONOP)CH_3_.^12^ Stable at −20 °C, **[1][B(C**_**6**_**F**_**5**_**)**_**4**_**]** slowly decomposes in solution and cannot be isolated in the crystalline state. Nevertheless, the *in situ* solution reactivity of **[1][B(C**_**6**_**F**_**5**_**)**_**4**_**]** demonstrated the electrophilic nature of the methylidene group, undergoing C–C coupling with ethyl diazoacetate, hydrogenolysis with H_2_, and ylide formation with PMe_3_. Inspired by these observations and the relative scarcity of iridium methylidene complexes,^13−15^ we now show that the corresponding [BAr^F^_4_]^−^ salt of **[1]**^**+**^ [Ar^F^ = 3,5-(CF_3_)_2_C_6_H_3_] can be isolated by low-temperature recrystallization from solution in good yield to form a room-temperature stable crystalline solid that undergoes SC–SC solid/gas reaction of the electrophilic Ir=CH_2_ group with H_2_, CO, and NH_3_.

**Figure 1:**
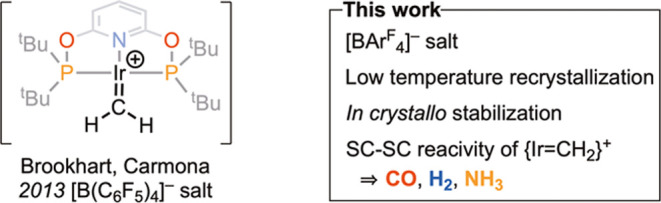
[Ir(^t^Bu-PONOP)(=CH_2_)][B(C_6_F_5_)_4_], **[1][B(C**_**6**_**F**_**5**_**)**_**4**_], and a description of this work.

Recent success in developing the organometallic solid/gas SC–SC reactivity of other cationic PONOP pincer complexes partnered with the [BAr^F^_4_]^−^ anion^16,17^ encouraged the synthesis of **[1][BAr**^**F**^_**4**_**]**. Key to isolating pure **[1][BAr**^**F**^_**4**_**]** was the use of [CPh_3_][BAr^F^_4_] as a limiting reagent (98%) and concentrated 1,2-F_2_C_6_H_4_ solutions (∼150 mg of [Ir complex]/2 cm^3^, −30 °C).^18^ Recrystallization at −30 °C (1,2-F_2_C_6_H_4_/heptane) gave dark-green rod-like crystals in 82% yield (Figure 2A). In the crystalline state, **[1][BAr**^**F**^_**4**_**]** is indefinitely stable at 298 K under an Ar atmosphere, as measured by solid-state NMR spectroscopy (SSNMR). These, and corresponding solution NMR (C_6_H_5_F, −30 °C), data are essentially the same as those reported for the cation in **[1][B(C**_**6**_**F**_**5**_**)**_**4**_**]**. Notably, the ^13^C{^1^H} 298 K SSNMR spectrum shows a broad (full width at half-maximum = 720 Hz) signal at δ 255 (lit.^12^ δ 252.2, C_6_D_5_Br) assigned to Ir=CH_2_, and a broad signal is observed in the ^31^P{^1^H} SSNMR spectrum at δ 184 (lit.^12^ δ 186.4).

**Figure 2:**
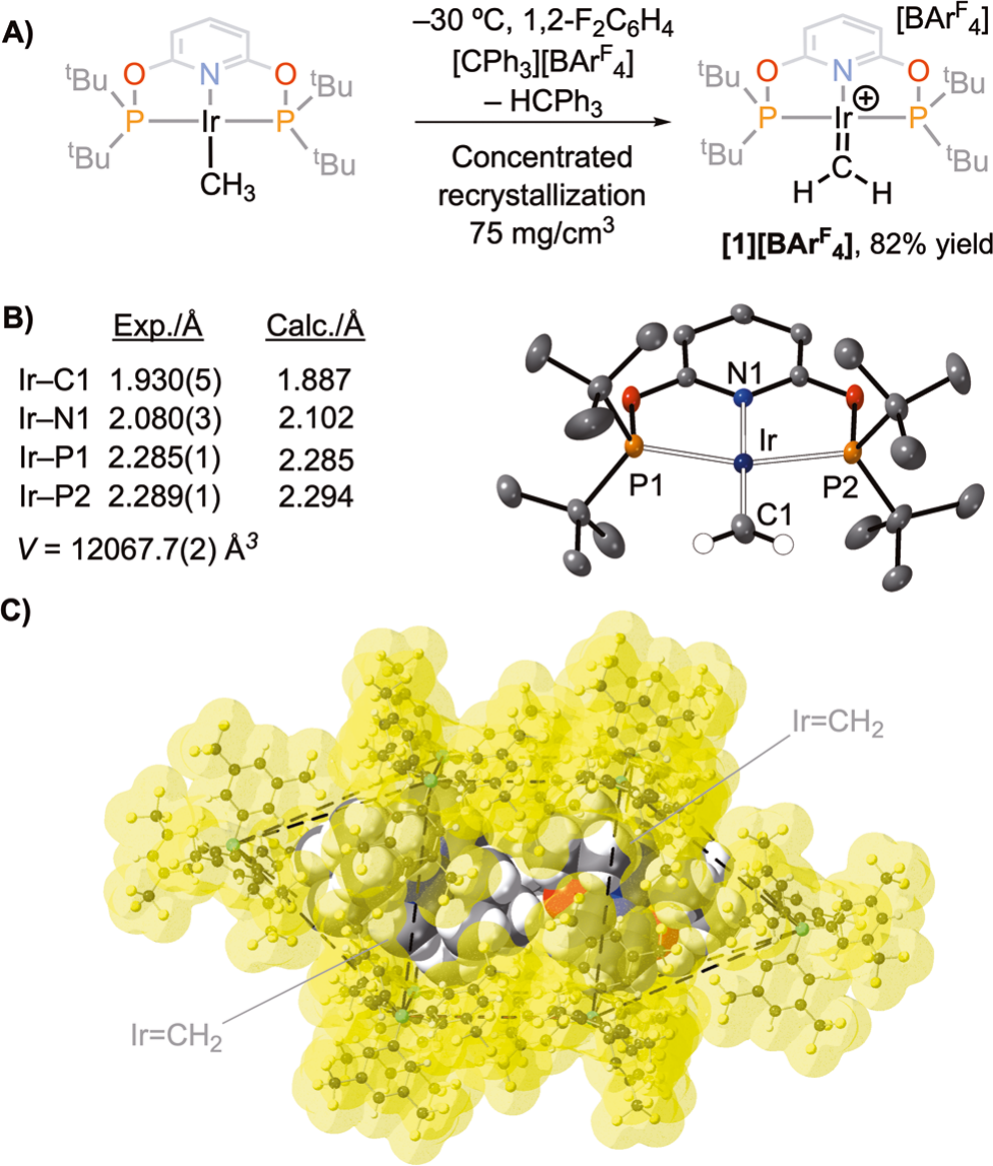
(A) Synthesis of **1[BAr**^**F**^_**4**_**]**. (B) Solid-state molecular structure of **[1]**^**+**^ (displacement ellipsoids shown at the 30% probability level). Selected H atoms are shown. (C) Diagram showing the arrangement of [BAr^F^_4_]^–^ anions (van der Waals radii).

Complex **[1][BAr**^**F**^_**4**_**]** crystallizes in space group *C*_2_/*c* [*V* = 12019.5(2) Å^3^; *Z*′ = 1] (Figure 2B). The Ir=C bond, 1.930(5) Å, is longer^19^ than that of the only other crystallographically characterized Ir=CH_2_ complex, neutral Ir{N(SiMe_2_CH_2_PPh_2_)_2_}(=CH_2_), 1.868(9) Å,^14,15^ but shorter than that of cationic Ir(I) diphenylcarbene complex [Ir(^t^Bu_2_PCH_2_P^t^Bu_2_)(=CPh_2_)(CO)][PF_6_], 1.996(8) Å.^20^ The hydrogen atoms associated with the methylidene were not located in the final difference map. The [BAr^F^_4_]^−^ anions form a bicapped square prismatic arrangement around two crystallographically equivalent cations (Figure 2C), as previously noted for related [M(PONOP)L_*n*_][BAr^F^_4_] complexes.^16,17^

The structure of **[1][BAr**^**F**^_**4**_**]** was fully optimized in the solid state using periodic density functional theory (DFT), and this provided excellent agreement for the Ir-PONOP fragment while somewhat underestimating the Ir=C1 distance (Figure 2B). The computed Wiberg bond index of 1.40 is consistent with the Ir=C1 bond character, and QTAIM gives an Ir=C1 BCP electron density, ρ(*r*), of 0.186 au.^21^ These values are both substantially larger than for the Ir–Me bond in [Ir(^t^Bu-PONOP)(H)(Me)]^+^ [WBI = 0.65; ρ(*r*) = 0.126 au].^22^ The [Ir=CH_2_] moiety is planar (∑_angles_ at C1 = 360.0°), and the CH_2_ ligand is rotated by 30.0° relative to the Ir(PONOP) plane. Full rotation of the Ir=CH_2_ unit has a very low barrier [<3.5 kcal/mol (see Figure S45)], and this reflects the near degeneracy of the two occupied dπ orbitals of the d^8^-T-shaped [Ir(PONOP)]^+^ fragment. The computed C1 ^13^C chemical shift in **[1]**^**+**^ (δ 247) is in good agreement with the value from SSNMR (δ 255), although the calculated value is significantly dependent on the orientation of the alkylidene ligand (Figure S43). The calculated ^13^C and ^1^H chemical shifts are not significantly affected when recomputed in the presence of the neighboring [BAr^F^_4_]^−^ anion, suggesting any ring current effects due to proximate Ar^F^ groups are minimal.^23^ Consistent with this, QTAIM, NCI, and IGMH analyses of the **[1][BAr**^**F**^_**4**_**]** ion pair identify only weak inter-ion interactions (Figures S40–S47). In terms of potential reactivity, the LUMO of **[1]**^**+**^ corresponds to a Ir–C1 π*-orbital heavily located on C1 and an MEP map identifies C1 as an electron-deficient site (see Figure S40). Both features suggest that the alkylidene will be susceptible to nucleophilic attack.

The solid/gas reactivity of **[1][BAr**^**F**^_**4**_**]** was explored. Addition of H_2_ (2 bara, 298 K, 24 h unoptimized, bara = bar absolute) resulted in a change in the color of the crystals from dark green to orange red (Figure S2). Application of vacuum to remove H_2_ and subsequent analysis by solution NMR spectroscopy (CD_2_Cl_2_, 298 K) showed the formation of known^24^ Ir(III) dihydride complex [Ir(^t^Bu-PONOP)(H)_2_][BAr^F^_4_], **[2][BAr**^**F**^_**4**_**]**. As H_2_ addition to **[1][B(C**_**6**_**F**_**5**_**)**_**4**_**]** at −60 °C in solution forms the related tetrahydride,^12^ we suggest this is also formed in the solid state and application of vacuum removes H_2_. Methane is presumably also formed. The ^31^P{^1^H} SSNMR spectrum of these crystals shows a broadened signal at δ 206 (lit. 206.8, C_6_D_5_Cl).^24^ This reaction is a SC–SC transformation, and the unit cell and motif of [BAr^F^_4_]^−^ anions are essentially unchanged from those of **[1][BAr**^**F**^_**4**_**]** (Figure S50).

When crystalline **[1][BAr**^**F**^_**4**_**]** is placed under CO (2 bara), there is a change in color from dark green to golden yellow over 19 h (Figure S30). Single-crystal X-ray diffraction of the resulting crystals showed the formation of known iridium(I) CO complex^22^ [Ir(^t^Bu-PONOP)(CO)][BAr^F^_4_], **[3][BAr**^**F**^_**4**_**]** (Figure 3), in a SC–SC transformation. The structural metrics of the cation are essentially the same as those of the previously reported [PF_6_]^−^ salt,^25^ while the [BAr^F^_4_]^−^ anions retain the same bicapped square prismatic motif as in **[1][BAr**^**F**^_**4**_**]**. Analysis of the bulk crystalline material by ^31^P{^1^H} SSNMR showed this reaction to be quantitative, with a resonance observed at δ 207.8 (lit.^22^ δ 205.0, C_2_Cl_4_D_2_), while in the ^13^C{^1^H} SSNMR spectrum, the CO group is observed at δ 182.1 (lit.^22^ δ 182.2, C_2_Cl_4_D_2_). Infrared spectroscopy showed a ν(CO) at 2008 cm^–1^. As described for other rhodium and iridium carbene complexes,^14,20,26^ this reaction likely occurs with formation of ketene, H_2_C=C=O, by sequential attack of two molecules of CO at {Ir=CH_2_} (eq 1), which is then expelled from the crystalline lattice. Due to the small amounts formed, most likely as the diketene dimer, repeated attempts to detect its formation were unsuccessful, as described previously.^14^ Nevertheless, this solid–gas reaction occurs with retention of crystallinity.
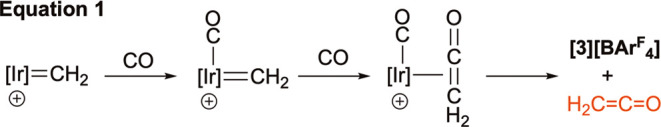
1**[1][B(C**_**6**_**F**_**5**_**)**_**4**_**]** reacts with PMe_3_ in solution to initially give phosphonium ylid [Ir(^t^Bu-PONOP)(CH_2_PMe_3_)][B(C_6_F_5_)_4_]. Wanting to explore if similar reactivity occurred under solid/gas conditions with an appropriate gaseous nucleophile, **[1][BAr**^**F**^_**4**_**]** crystalline **[1][BAr^F^_4_]** was exposed to an atmosphere of NH_3_ (1.2 bara, 298 K) for 24 h (unoptimized). During this time, the crystals changed from dark green to yellow orange in color (Figure S4). This reaction is a SC–SC transformation, and a resulting single-crystal X-ray diffraction study showed the product to be iridium(I) methylamine complex [Ir(^t^Bu-PONOP)(NH_2_Me)][BAr^F^_4_], **[4][BAr**^**F**^_**4**_**]** (Figure 3): Ir–N2, 2.104(5) Å; N2–C1, 1.451(10) Å. In the ^1^H NMR spectrum (CD_2_Cl_2_, 298 K), aside from the ^t^Bu-PONOP and [BAr^F^_4_]^−^ resonances, a broad singlet at δ 3.77 (relative integral 2 H) is assigned to Ir–NH_2_ and a triplet at δ 2.97 [relative integral 3 H, *J*(HH) = 6.5 Hz], which remains a triplet in the ^1^H{^31^P} NMR spectrum, is assigned to the methyl group. A COSY NMR experiment showed that these two signals are mutually coupled. In the ^13^C{^1^H} NMR spectrum, a new signal observed at δ 40.4 is assigned to the Ir–NH_2_*C*H_3_ group. A single environment is observed in the ^31^P{^1^H} NMR spectrum (δ 182.4).^27^ The corresponding SSNMR spectra are essentially the same. These NMR and metrical data are consistent with previously reported Ir-NH_2_Me complexes.^28,29^

**Figure 3:**
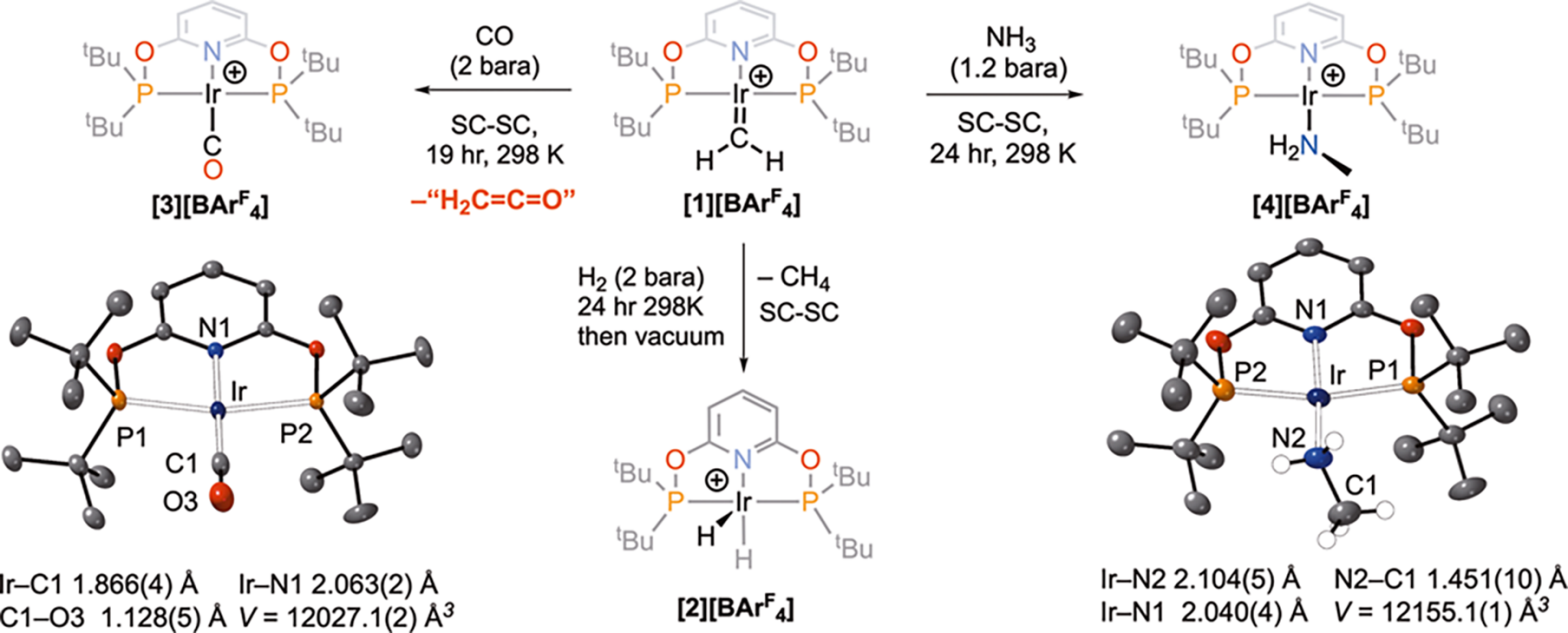
*In crystallo* reactions of **[1][BAr^F^_4_]** with H_2_, CO, and NH_3_. [BAr^F^_4_]^−^ anions are not shown. Displacement ellipsoids are shown at the 30% probability level. Selected H atoms are shown. SC–SC = single-crystal to single-crystal.

Stopping this solid/gas reaction after 1 h by removing the NH_3_ atmosphere and dissolving the resulting crystalline material in 1,2-F_2_C_6_H_4_ revealed that while all of the methylidene starting material, **[1][BAr**^**F**^_**4**_**]**, had been consumed, there was a new dominant species (85%) observed alongside the final product, **[4][BAr**^**F**^_**4**_**]** (Scheme 1). If the reaction is allowed to proceed *in crystallo* after NH_3_ removal, then **[4][BAr**^**F**^_**4**_**]** is the final product after 24 h, showing that this new species is an intermediate and does not require NH_3_ to form **[4][BAr**^**F**^_**4**_**]**. In the ^1^H NMR spectrum, a new relative integral 2 H hydride signal is observed at δ −8.2 [t, *J*(PH) = 15.8 Hz], characteristic of *trans*-hydrides. A relative integral 1 H signal is observed at δ 11.86 in the region associated with carbene M=C*H*R groups. These data tentatively identify this intermediate as aminocarbene complex [Ir(^t^Bu-PONOP)(=CHNH_2_)(H)_2_][BAr^F^_4_], **[5][BAr**^**F**^_**4**_**]**, a hypothesis supported by DFT calculations (see below). These findings demonstrate that **[5][BAr**^**F**^_**4**_**]** is the kinetic product of the reaction and **[4][BAr**^**F**^_**4**_**]** is the thermodynamic product. **[5][BAr**^**F**^_**4**_**]** is closely related to previously reported Ir(PNP)(=CHR)(H)_2_ [PNP = {N(2-P^i^Pr_2_-4-Me-C_6_H_3_)_2_}^−^, where R = morpholine]: δ_hydride_ −8.92, δ_=C*H*R_ 12.70.^30^

**Scheme 1:**
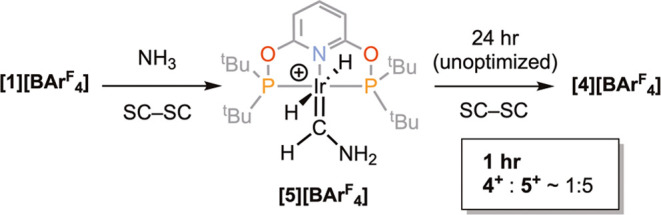
Kinetic, **[5][BAr^F^_4_]**, and Thermodynamic, **[4][BAr^F^_4_]**, Products of the Reaction of **[1][BAr^F^_4_]** with NH_3_ ([BAr^F^_4_]^–^ anions not shown)

The reaction of **[1][BAr**^**F**^_**4**_**]** with NH_3_ was modeled in the solid state using periodic DFT calculations (Figure 4). The initial addition of NH_3_ at the electrophilic methylene group forms an ammonium ylid, **I**^**+**^.^31^ N–H transfer to iridium then gives an Ir(III) aminomethyl species, **II**^**+**^, that can then undergo α-H transfer with a barrier of 0.8 kcal/mol to give *trans*-dihydride aminocarbene complex **5**^**+**^ at −14.1 kcal/mol. The formation of **5**^**+**^ is reversible and if coupled to a second C–H coupling results in the formation of a methylamine ligand, initially bound as a C–H σ-complex (**III**^**+**^). Rearrangement then forms Ir–NH_2_Me product **4**^**+**^. The free energy span for the formation of **4**^**+**^ from **5**^**+**^ is 23.7 kcal/mol. The overall profile is consistent with the rapid formation of **5**^**+**^ as the kinetic product (Δ*G*^⧧^ = 8.1 kcal/mol, and Δ*G* = −14.1 kcal/mol) followed by a relatively slow conversion to **4**^**+**^ as the thermodynamic product (Δ*G*^⧧^ = 23.7 kcal/mol, and Δ*G* = −6.4 kcal/mol), as seen experimentally.

**Figure 4:**
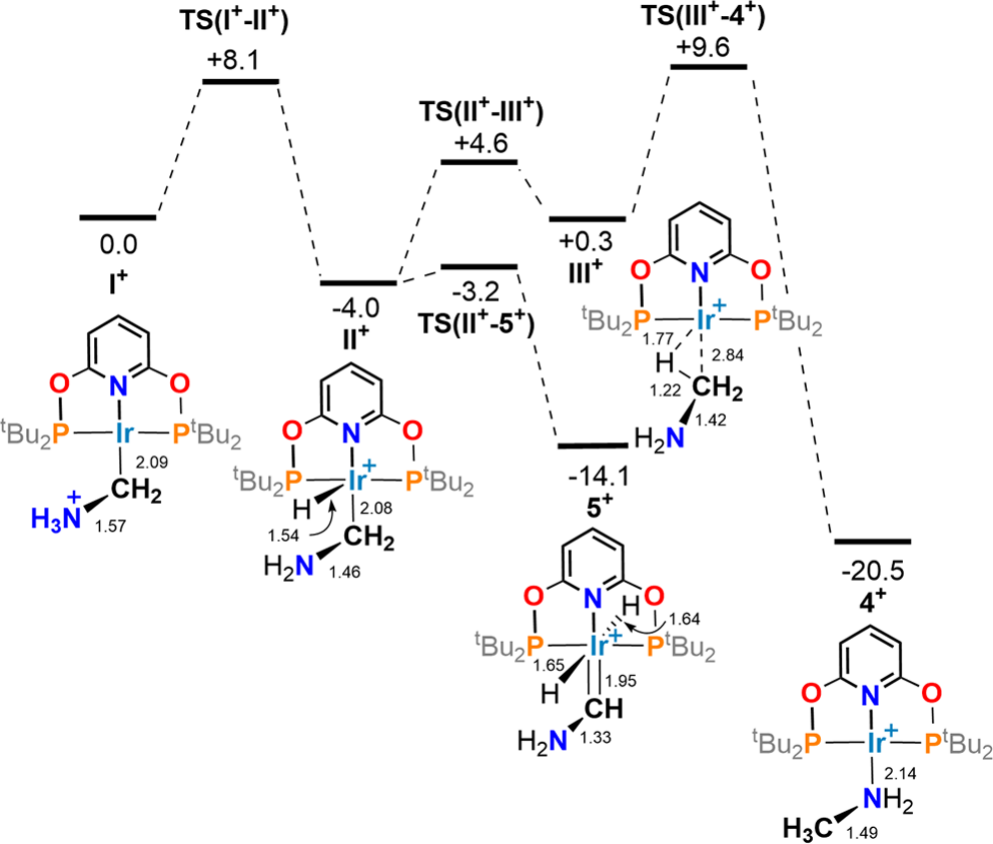
Computed free energy reaction profile (kcal/mol; periodic DFT based on **[1][BAr**^**F**^_**4**_**]**) for the rearrangement of the methylamine ylid, **I**^**+**^, to methylamine adduct **[4]**^**+**^. Selected distances around the Ir–CH_2_NH_3_ moiety (Å are also provided).

A similar reaction profile was computed for the isolated molecular cation, albeit with variations of ≲3 kcal/mol in individual stationary points that indicate some impact of the solid-state environment on reactivity.^32^ Experimentally, addition of NH_3_ (1.2 bara) to **[1][BAr**^**F**^_**4**_**]** in a C_6_H_5_F solution at 20 °C resulted in the rapid formation of **[5][BAr**^**F**^_**4**_**]**, which was then converted into **[4][BAr**^**F**^_**4**_**]**.^33^ However, other, as yet unidentified, species were also observed, meaning that a direct analysis of solution versus single-crystal reactivity is not straightforward. What is clear is that the solid/gas reactivity proceeds cleanly compared with that in solution, an observation we and others have made previously.^34^

As well as being an example of a SC–SC reaction of an electrophilic Ir methylidene unit with a nucleophile, the formation of **[4][BAr**^**F**^_**4**_**]** is directly relevant to deactivation pathways observed for second-generation Grubbs-type Ru methylidene complexes in metathesis chemistry, as outlined by Fogg and co-workers.^35,36^ Here small nucleophilic alkyl amines can attack a {Ru=CH_2_} group, resulting in decomposition of the organometallic complex and formation of free methylamines. The formation of **[4][BAr**^**F**^_**4**_**]** thus offers a model pathway for this process, where the thus-formed methylamine remains bound to the metal center, albeit on Ir not Ru. The [Rh=CH_2_]^+^ fragment has also been suggested to react with NH_3_ in the gas phase to form methylamine.^37^

In conclusion, we have demonstrated that low-temperature recrystallization of a reactive organometallic methylidene complex generates an *in crystallo*-stabilized complex that can undergo subsequent SC–SC solid/gas reaction at room temperature. This general methodology offers a conceptually simple route for studying organometallic reactivity in the crystalline phase. It will be interesting to see if this approach is a more general one for solution-based organometallic chemistry, allowing the reactivity of species to be studied in the crystalline phase that are challenging to isolate or observe by using more conventional routes.

## Supporting Information Available

Full details of the experimental methods, characterization data for all of the new complexes, single-crystal X-ray diffraction studies, and coordinates and energies of computed intermediates (PDF)
Gas phase reactivity based on **[1]**^+^ (XYZ)
Solid-state reactivity based on **[1][BAr^F^4**] (XYZ)
Solid-state reactivity based on **[4][BAr^F^_4_]** (XYZ)

## Supplementary Material

om4c00119_si_001.pdf

om4c00119_si_002.xyz

om4c00119_si_003.xyz

om4c00119_si_004.xyz
