# Supplementary material for: Solid/Gas In Crystallo Reactivity of an Ir(I) Methylidene Complex
Source: Organometallics. 2024 Jun 6;43(24):3137–42. doi: 10.1021/acs.organomet.4c00119 (PMC11673653; doi:10.1021/acs.organomet.4c00119)
Supplement: Supplementary file 1 — om4c00119_si_001.pdf [file om4c00119_si_001.pdf]

Supporting information for

Solid/Gas *InCrystallo* reactivity of an Ir(I) Methylidene Complex.

Kristof M. Altus,[a] M. Arif Sajjad,[b] Matthew R. Gyton,[a] Adrian C. Whitwood,[a] Samuel J. Page,[c] Stuart A. Macgregor*[b] and Andrew S. Weller.*[a]

[a]Department of Chemistry, University of York, York, YO10 5DD, UK
[b] EaSTCHEM School of Chemistry, North Haugh, University of St. Andrews, St Andrews KY16 9ST, UK
[c] Department of Chemistry, University of Durham, Durham, DH1 3LE, UK

Email:

andrew.weller@york.ac.uk

sam38@st-andrews.ac.uk

Table of Contents

Experimental S3
General considerations S3
Synthesis of Compounds S5
NMR and IR Spectra S9
[1][BAr$^F_4$] S9
[2][BAr$^F_4$] S12
[3][BAr$^F_4$] S14
[4][BAr$^F_4$] S17
Visual representation of the single-crystal to single-crystal reaction of **[1][BAr$^F_4$]** with CO over time. S22
NMR analysis of the reaction between **[1][BAr$^F_4$]** and NH_3. .... S24
Solid-State Calculations S26
Molecular Calculations S26
Electronic Structure Analyses. S27
[1][BAr$^F_4$] S27
[(^{t}Bu-PONOP)Ir(Me)(H)][BAr$^F_4$] S32
NMR Chemical Shift Calculations on [1]$^+$. S34
[1][BAr$^F_4$] Ion-pair Interactions S36
Reaction of [1][BAr$^F_4$] with NH_3 .... S38
Crystallographic data S43
[1][BAr$^F_4$] S44
[2][BAr$^F_4$] S45
[3][BAr$^F_4$] S46
[4][BAr$^F_4$] S47
References S48

Experimental

General considerations

All manipulations, unless otherwise stated, were performed under an inert (argon or nitrogen, BOC, N4.8 purity) atmosphere using standard Schlenk line and glovebox (<0.1 ppm H_2O/O_2) techniques. Glassware was oven-dried at 140 °C overnight prior to use. All solvents were degassed by three successive freeze-pump-thaw cycles and stored over activated 3 Å molecular sieves under inert gas in resealable glass ampoules fitted with PTFE high vacuum stopcocks (J. Young or Rotaflo HP). Hexane was dried using a commercially available solvent system by passage through stainless steel columns containing activated alumina. Heptane was purchased anhydrous from Sigma-Aldrich and decanted by cannula into resealable glass ampoules and stored as above. CD_2Cl_2 and 1,2-$C_6H_4F_2$ (pre-dried by stirring over activated alumina) were dried over CaH_2, before vacuum transfer and storage as above. H_2 (N5.0), CO and NH_3 (N3.8) were purchased from CK special gasses ltd. All other chemicals were from commercial sources and used without further purification.

Solution state NMR data were collected on a Bruker AVIIIHD 500 MHz or AVIIIHD 600 MHz Widebore spectrometer at the temperatures specified. Multiplicity abbreviations: singlet (s), doublet (d), triplet (t), virtual triplet (vt), quartet of quartets (qq), septet (sept). Coupling constants are designated as $^{n}J_{X\text{-}Y}$ for each respective nucleus. The J values reported for the PONOP ligand represent the *apparent* coupling between adjacent peaks of the apparent triplet. Solution ^{1}H and $^{13}C\{^{1}H\}$ NMR spectra were referenced to the residual solvent peaks. All NMR assignments for complexes were made on the basis of 1D and 2D NMR data as well as crystallographic analysis were relevant. $^{31}P\{^{1}H\}$ solution spectra were referenced externally to 85% H_3PO_4 in D_2O All dichloromethane-d2 solution phase NMR were prepared on a greaseless high vacuum line (<5 x 10^{-2} mbar) by condensation of the solvent under static vacuum onto solid samples in 5 mm thin wall NMR tubes fitted with high vacuum PTFE (J. Young) valves.

Solid-state NMR samples were prepared in an argon-filled glovebox by pre-loading 60-100 mg (4.0 mm) or 10-20 mg (2.0 mm) of crushed material into zirconia solid-state NMR rotor and sealed with Kel-F, vespel or zirconia caps. Solid-state NMR data were obtained on Bruker Avance III HD spectrometers, operating at 100.63 MHz ($^{13}C\{^{1}H\}$), 100.56 MHz ($^{13}C\{^{1}$ H$\}$), 162.04 MHz ($^{31}P\{^{1}H\}$), 161.99 MHz ($^{31}P\{^{1}H\}$) at the MAS rates and temperatures specified. All $^{13}C\{^{1}H\}$ CP MAS spectra were referenced to adamantane where the upfield methine resonance was taken to be δC = 29.5 ppm, secondarily referenced to δC($SiMe_4$) = 0.0 ppm. $^{31}P\{^{1}H\}$ CP MAS spectra were referenced to triphenylphosphine (δP = -9.3 ppm relative to H_3PO_4) or calcium hydrogen phosphate (δP = 1.4 ppm relative to H_3PO_4). Solid-state NMR

spectra were recorded at varied MAS rates to determine isotropic chemical shifts. Elemental analysis was carried out by Ms Orla McCullough at London Metropolitan University.

Synthesis of (^{t}Bu-PONOP)IrMe[S1] and $[CPh_3][BAr^F_4]$[S2] were carried out according to literature procedures.

Synthesis of Compounds

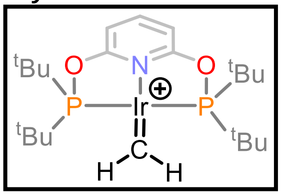

[(^{t}Bu-PONOP)IrCH$_2$][BAr$^F{}_4$], ([1][BAr$^F{}_4$]): To and oven dried Schlenk flask under argon was added (^{t}Bu-PONOP)IrMe (96.2 mg, 0.158 mmol), [CPh$_3$][BAr$^F{}_4$] (172 mg, 0.155 mmol) and a magnetic stir bar. The two solids were mixed in the solid-state until the mixture became homogeneous and was finely powdered (powdering the two substrates is crucial for a successful reaction). The flask was cooled to –30°C prior to addition of 1,2-$F_2C_6H_4$ (DFB) (1-2 mL). Upon addition of DFB, the solids dissolved immediately turning the solution a dark emerald green. The reaction was stirred for approximately one minute ensuring all solids dissolved, at which point, hexane (~50 mL) was added at once which precipitated a green solid. Keeping the suspension at –30 °C the mother liquor was decanted via cannula filtration. The green solid was then dried under reduced pressure. At this point the solid is stable at room temperature and can be manipulated under argon or nitrogen.

* Notes for synthesis: [CPh$_3$][BArF$_4$] must be kept as the limiting reagent to reduce chance of recrystalising unreacted starting material, which cannot be separated from the product. Limiting [CPh$_3$][BArF$_4$] too much also causes decomposition during recrystallisation. Freshly distilled 1,2-$F_2C_6H_4$ must be used to avoid decomposition as best as possible. Furthermore, the reaction should be conducted as concentrated as possible as this helps reduced side product formation from reaction with trace impurities in the solvent.

Recrystallisation procedure: The green solid was added to a J-young's ampoule and dissolved in DFB (1 mL) that had been pre-cooled to –30°C. Heptane that had been pre-cooled to –30 °C was then carefully layered on top of the DFB. the ampoule was placed in a glove box freezer at –30 °C. Full recrystallisation of the solid took on average 3-4 days giving dark green rod/needle like crystals. The mother liquor was decanted and the crystals washed with room temperature heptane (2 x 10 mL) before drying under vacuum over night at 2 x 10^{-2} mbar giving 187 mg of dark green crystals in 82 % yield. Solution-state NMR data was consistent with the literature reported values for **[1][B(C$_6$F$_5$)$_4$]**, NMR data and spectra have been included for reference.

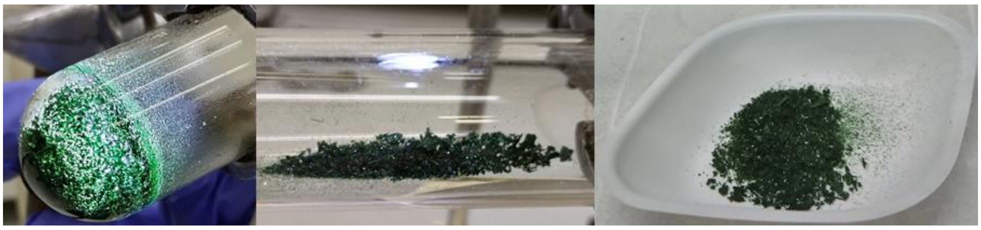

*Figure S1. Crystalline material of **[1][BAr$^F{}_4$]**.*

^{1}H NMR (C_6H_5F, 500 MHz, 243 K): δ 18.5 (t, $^3J_{P\text{-}H}$ = 7.5 Hz, 2H, Ir=C***H***$_2$), 8.16 (s, 8H, *ortho*-BAr$^F{}_4$), 6.46 (d, $^3J_{H\text{-}H}$ = 8.2 Hz, 2H, *m*-PONOP), 1.07 (vt, *J* = 8.5 Hz, 36 H, (C(C***H***$_3$)$_3$))

^{1}H{^{31}P} NMR (C_6H_5F, 500 MHz, 243 K): δ 18.5 (s, Ir=C***H***$_2$) 8.16 (s, *ortho*-BAr$^F{}_4$), 6.46 (d, *J* = 8.5 Hz, *m*-PONOP), 1.07 (s, 36 H, (C(C***H***$_3$)$_3$))

^{31}P{^{1}H} NMR (C_6H_5F, 202 MHz, 243 K): δ 180 (s)

^{31}P{^{1}H} CPMAS SSNMR (10 KHz, 162 MHz, 298 K): δ 184 (broad)

^{13}C{^{1}H} CPTOSS MAS NMR (10 KHz, 100 MHz, 298 K): δ 255 (Ir-***C***H$_2$, broad), 161(v. broad), 129, 101, 41, 25

Elemental analysis for $C_{54}H_{53}BF_{24}IrNO_2P_2$ (C, H, N;): Calculated C, 44.15; H, 3.64; N, 0.95; Found C, 44.55; H, 3.65; N, 0.91.

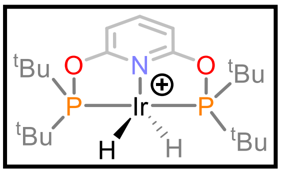

[(^{t}Bu-PONOP)IrH$_2$][BAr$^F{}_4$], ([2][BAr$^F{}_4$]): To an oven dried J-young's NMR tube was added **[1][BAr$^F{}_4$]** (15.0 mg, 0.100 mmol). To the crystals was added dihydrogen (2 bar absolute) and the reaction left for 24 hours. The dihydrogen atmosphere was then removed under vacuum at 2 x 10^{-2} mbar for 24 hours. Recovery of the crystals from the NMR tube gave 11.0 mg of yellowish-orange crystals. NMR data is consistent with the literature reported values.

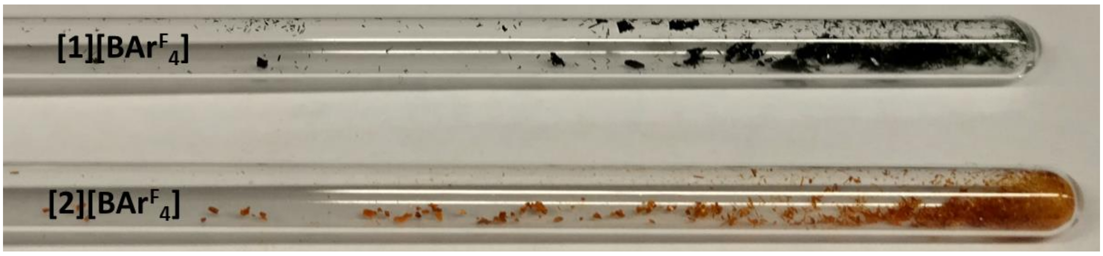

*Figure S2. Visualisation of the SC-SC reaction of **[1][BAr$^F{}_4$]** with H_2.*

^{1}H NMR (CD_2Cl_2, 500 MHz, 243 K): δ 7.96 (t, $^3J_{H\text{-}H}$ = 8.2 Hz, 1H, *p*-PONOP), 7.70 (s, *ortho*-BAr$^F{}_4$, 8H) 7.53 (s, *para*-BAr$^F{}_4$, 4 H), 7.05 (d, $^3J_{H\text{-}H}$ = 8.2 Hz, 2H, *m*-PONOP), 1.26 (vt, *J* = 8.5 Hz, 36H, (C(C***H$_3$***)$_3$)), -25.5 ($^2J_{P\text{-}H}$ = 12 Hz, 2H).

^{31}P{^{1}H} NMR (CD_2Cl_2, 202 MHz, 243 K): δ 206

^{31}P{^{1}H} CPMAS SSNMR (10 KHz, 162 MHz, 298 K): δ 205

^{13}C{^{1}H} CPTOSS MAS NMR (10 KHz, 100 MHz, 298 K): δ 162, 132, 117, 103, 40.5, 25.9

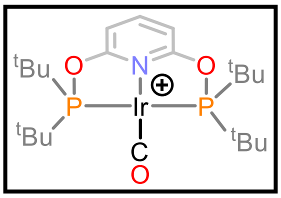

[(^{t}Bu-PONOP)IrCO][BAr$^F_4$] ([3][BAr$^F_4$]): To an oven dried J-young's NMR tube was added **[1][BAr$^F_4$]** (15.0 mg, 0.100 mmol). To the crystals was added carbon monoxide (2 bar absolute) and the reaction left for 19 hours. The carbon monoxide atmosphere was then removed and replaced with argon. Recovery of the crystals from the NMR tube gave 13.0 mg of golden yellow crystals. NMR data is consistent with the literature reported values. Solution-state ^{1}H and ^{31}P NMR as well and ^{13}C and ^{31}P SSNMR have been included.

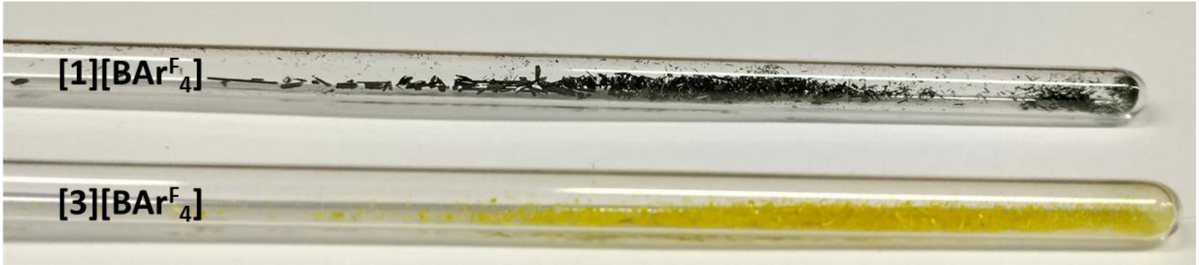

*Figure S3. Visualisation of the SC-SC reaction of **[1][BAr$^F_4$]** with CO to give **[3][BAr$^F_4$]**.*

^{1}H NMR (CD_2Cl_2, 500 MHz, 243 K): δ 7.96 (t, $^3J_{H-H}$ = 8.2 Hz, 1H, *p*-PONOP), 7.00 (d, $^3J_{H-H}$ = 8.2 Hz, 2H, *m*-PONOP), 1.36 (vt, *J* = 8.5 Hz, 36H, (C(C***H*₃**)₃)).

^{31}P{^{1}H} NMR (CD_2Cl_2, 202 MHz, 243 K): δ 204.

^{31}P{^{1}H} CPMAS SSNMR (10 KHz, 162 MHz, 298 K): δ 207.

^{13}C{^{1}H} CPTOSS MAS NMR (10 KHz, 100 MHz, 298 K): δ.182 (***C***O), 165, 161, 134, 131, 130, 126, 123, 118, 103, 102, 42(***C***(CH_3)$_3$), 26 (C(***C***H_3)$_3$).

FT-IR (ATR): νCO 2008 cm^1 (s).

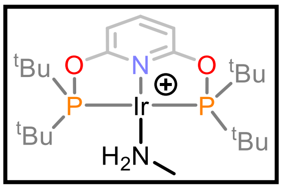

[(^{t}Bu-PONOP)IrNH$_2$Me][BAr$^F{}_4$] ([4][BAr$^F{}_4$]): To an oven dried J-young's NMR tube was added **[1][BAr$^F{}_4$]** (15.0 mg, 0.10.0 mmol). To the crystals was added ammonia (1.2 bar absolute) and the reaction left for 24 hours. The ammonia atmosphere was then removed and replaced with argon. Recovery of the crystals from the NMR tube gave 12.1 mg of yellowish-orange crystals.

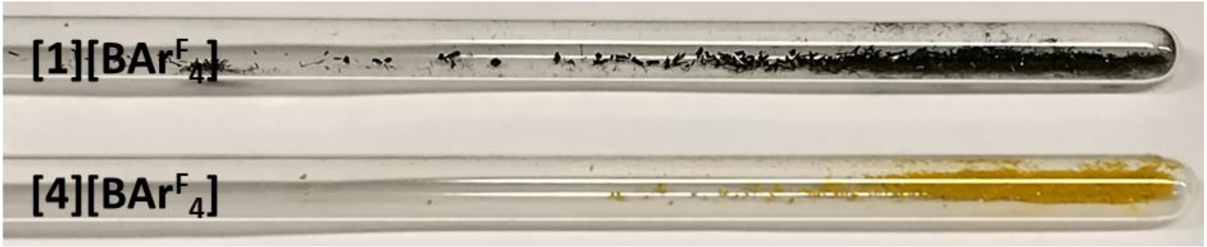

*Figure S4. Visualisation of the SC-SC reaction of **[1][BAr$^F{}_4$]** with NH_3 to give **[4][BAr$^F{}_4$]**.*

^{1}H NMR (CD_2Cl_2, 500 MHz, 298 K): δ 7.79 (t, $^3J_{H\text{-}H}$ = 8.2 Hz, 1H, *para*-PONOP), 7.72 (s, 8H, *ortho*-BAr$^F{}_4$), 7.56 (s, 4H, *para*-BAr$^F{}_4$), 6.62 (d, $^3J_{H\text{-}H}$ = 8.2 Hz, 2H, *meta*-PONOP), 3.77 (br s, 2H, NH_2), 2.97 (t, $^3J_{H\text{-}H}$ = 6.6 Hz, 3H, NCH_3), 1.42 (vt, J = 8.5 Hz, 36H, (C(C***H*$_3$**)$_3$)).

^{1}H{^{31}P} NMR (CD_2Cl_2, 500 MHz, 243 K): 7.79 (t, $^3J_{H\text{-}H}$ = 8.0 Hz, 1H, *para*-PONOP), 7.72 (s, 8H, *ortho*-BAr$^F{}_4$), 7.56 (s, 4H, *para*-BAr$^F{}_4$), 6.62 (d, $^3J_{H\text{-}H}$ = 8.2 Hz, 2H, *meta*-PONOP), 3.77 (br s, 2H, NH_2), 2.97 (t, $^3J_{H\text{-}H}$ = 6.6 Hz, 3H), 1.42 (s, 36H, (C(C***H*$_3$**)$_3$)).

^{31}P{^{1}H} NMR (CD_2Cl_2, 202 MHz, 298 K): δ 182 (s).

^{31}C{^{1}H} NMR (CD_2Cl_2, 151 MHz, 298 K): δ 165 (apparent triplet, $J_{P\text{-}C}$ = 3.7 Hz, *ortho*-PONOP), 162 (*ipso*-BAr$^F{}_4$), 139 (*para*-PONOP), 135 (*meta-BAr$^F{}_4$)*, 129 (qq, $^2J_{F\text{-}C}$ = 31.5 Hz; $^4J_{F\text{-}C}$ = 2.82 Hz,***C***CF_3), 124 (q, $^1J_{F\text{-}C}$ = 274 Hz, ***C***F_3), 117 (sept, $^3J_{F\text{-}C}$ =4.3 Hz, *para-BAr$^F{}_4$)*, 102 (apparent triplet, $J_{P\text{-}C}$ = 2.5 Hz, *meta*-PONOP), 42.1 (vt, $J_{P\text{-}C}$ = 10 Hz, ***C***(CH_3)$_3$), 40.4 (H_2N***C***H_3), 27.9 (vt, $J_{P\text{-}C}$ = 3.7 Hz, C(***C***H_3)$_3$).

^{31}P{^{1}H} CPMAS SSNMR (10 KHz, 162 MHz, 298 K): δ 185.

^{13}C{^{1}H} CPTOSS MAS NMR (10 KHz, 100 MHz, 298 K): δ 165.24, 163.29, 137.89, 134.83, 130.66, 125.97, 123.60, 118.36, 117.11, 101.59, 41.72, 38.75, 26.93.

NMR and IR Spectra

[1][BAr^F$_4$]

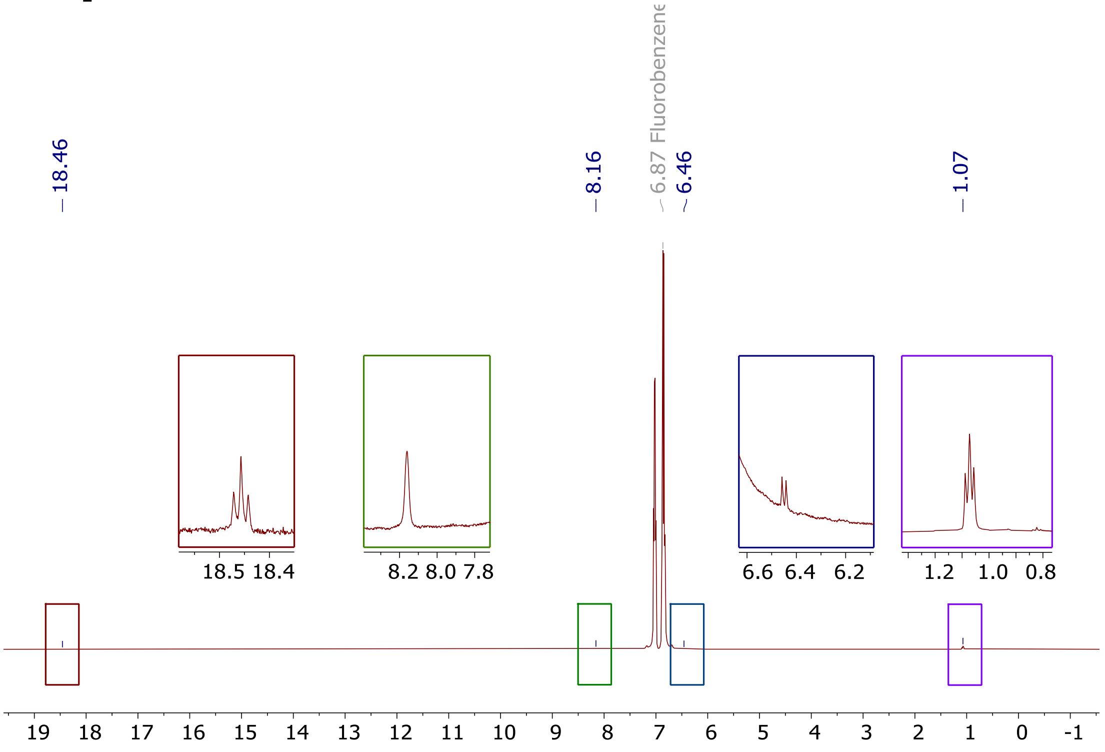

Figure S5. ^{1}H NMR (C_6H_5F, 500 MHz, 243 K) of crystalline ***[1][BAr^F$_4$]***.

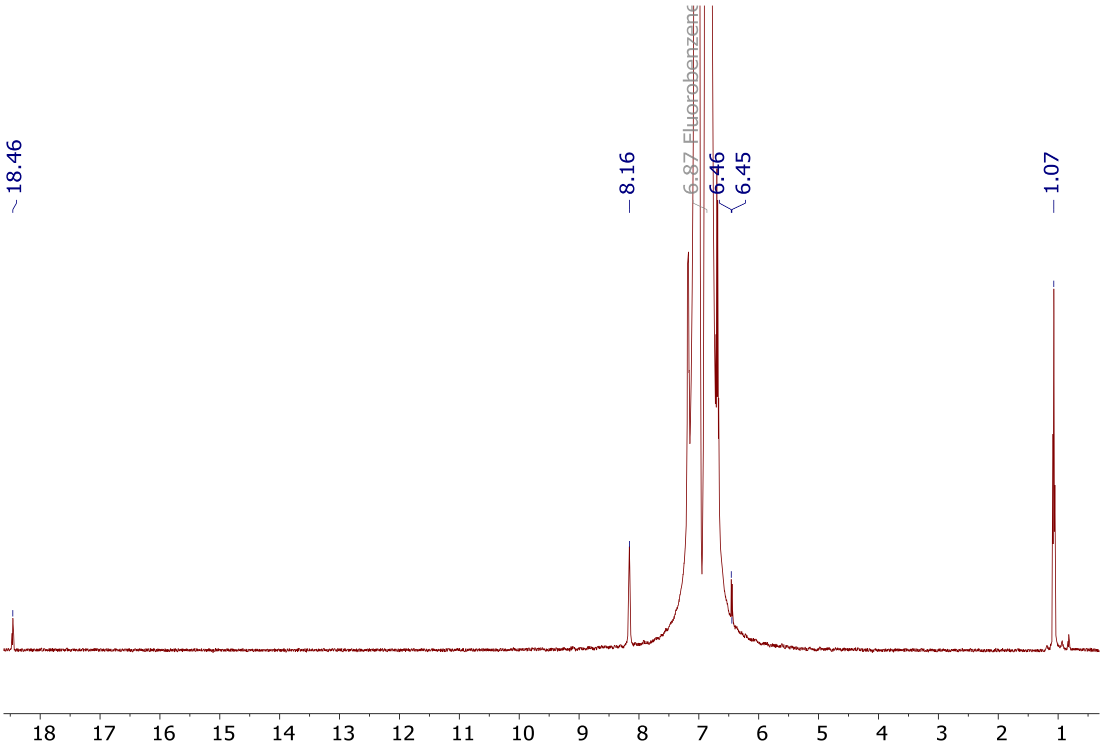

Figure S6. ^{1}H NMR spectrum (C_6H_5F, 500 MHz, 243 K) of crystalline ***[1][BAr^F$_4$]***. *Spectrum magnified.*

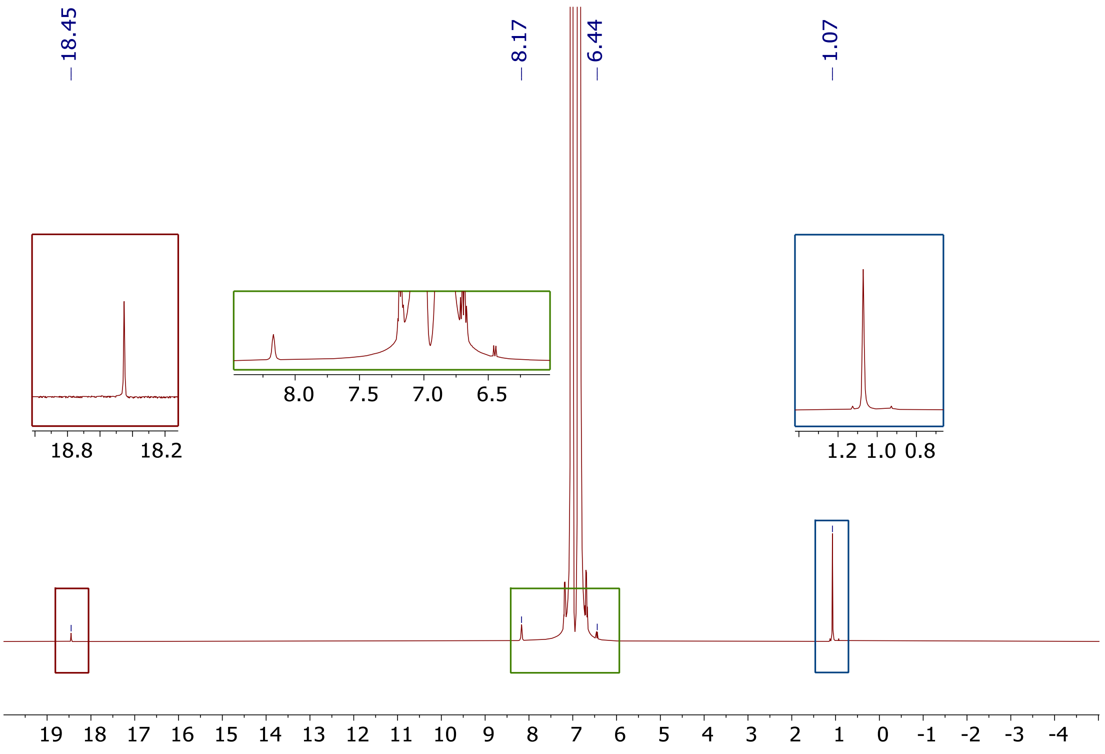

*Figure S7. ¹H{³¹P} NMR spectrum (C_6H_5F, 500 MHz, 243 K) of crystalline **[1][BAr^F$_4$]**.*

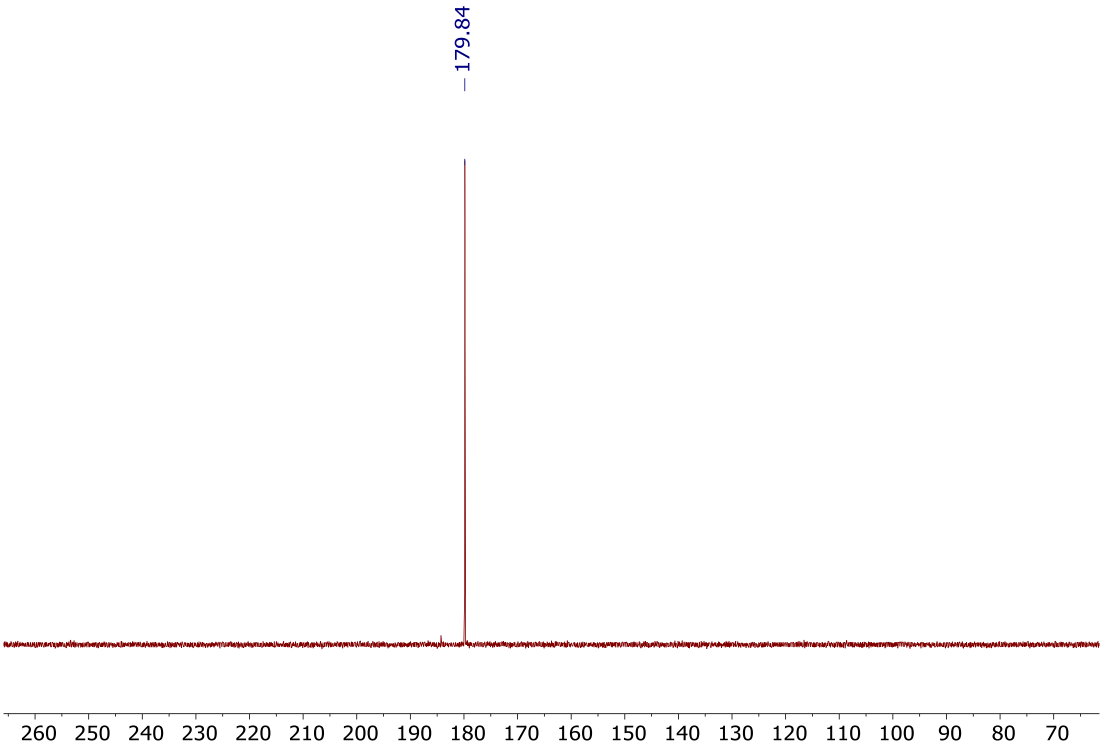

*Figure S8. ³¹P{¹H} NMR spectrum (C_6H_5F, 202 MHz, 243 K) of crystalline **[1][BAr^F$_4$]**. The peak at δ 184 corresponds to a decomposition product that grows in over time (10 % increase in 2 hours).*

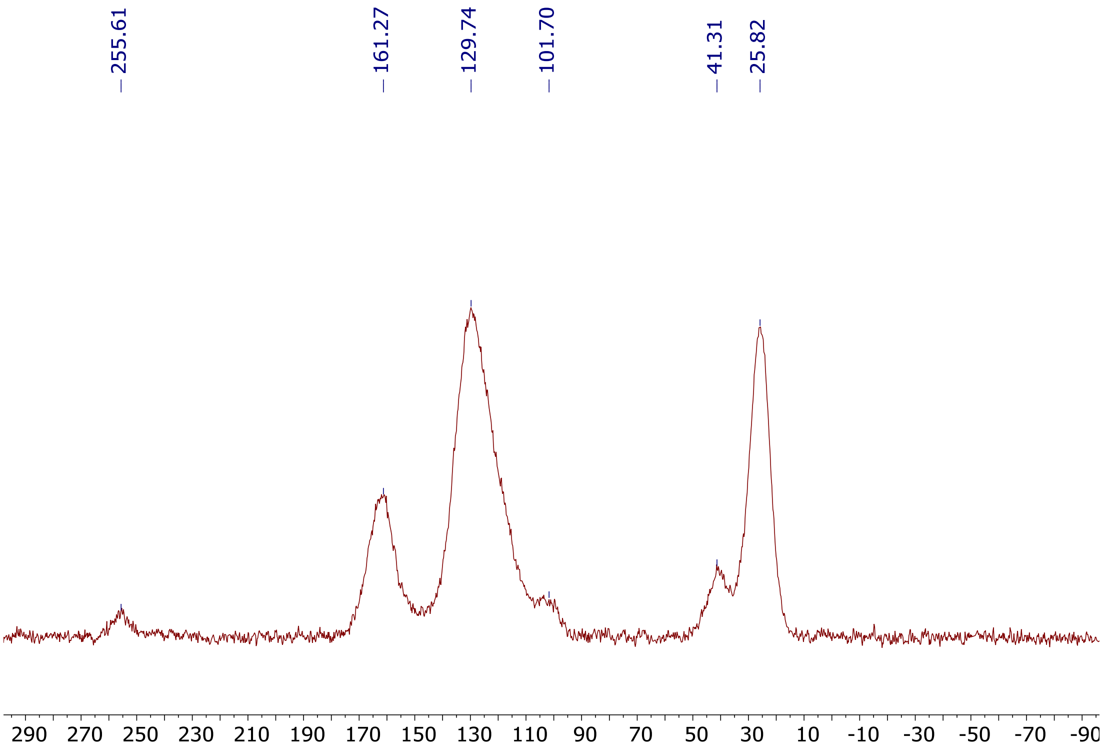

Figure S9. $^{13}C\{^{1}H\}$ *CPTOSS MAS NMR spectrum (10 KHz, 100 MHz, 298 K) of crystalline* ***[1][BAr$^{F}_{4}$]***.

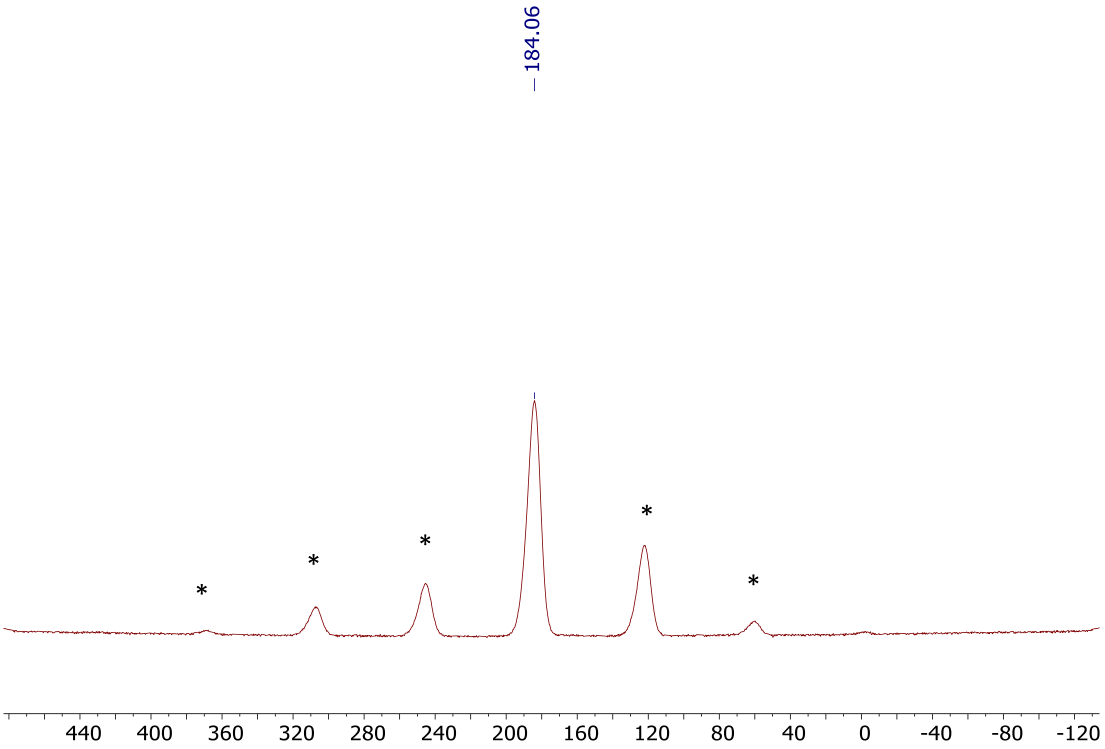

Figure S10. $^{31}P\{^{1}H\}$ *CPMAS SSNMR spectrum (10 KHz, 162 MHz, 298 K) of crystalline* ***[1][BAr$^{F}_{4}$]***.** indicates spinning side bands.*

[2][BArF4]

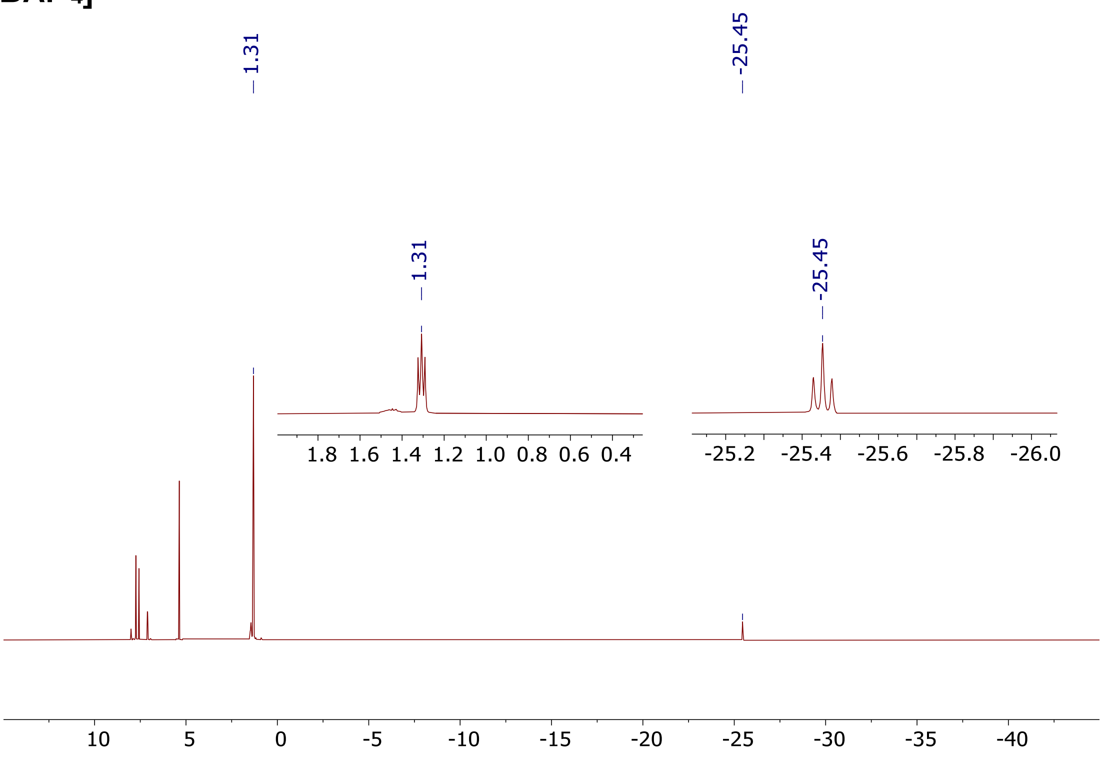

Figure S11. $^{1}H\{^{31}P\}$ NMR spectrum (CD_2Cl_2, 500 MHz, 243 K) of crystalline ***[2][BArF4]****.*

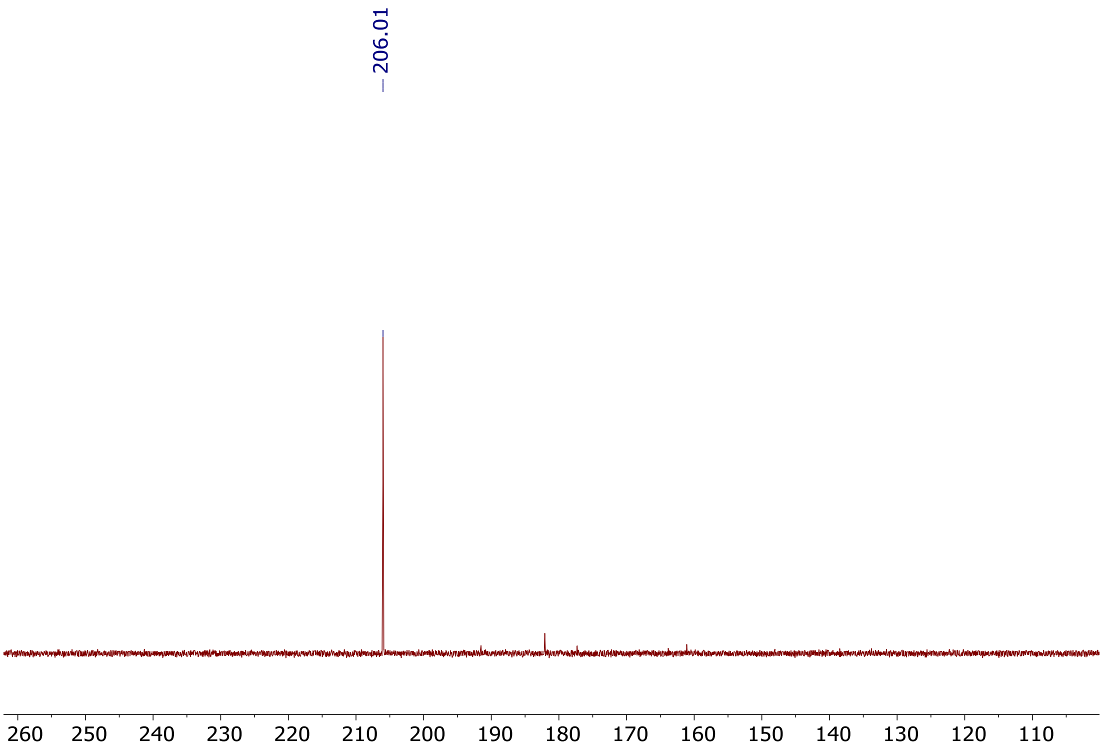

Figure S12. $^{31}P\{^{1}H\}$ NMR spectrum (CD_2Cl_2, 202 MHz, 243 K) of crystalline ***[2][BArF4]****.*

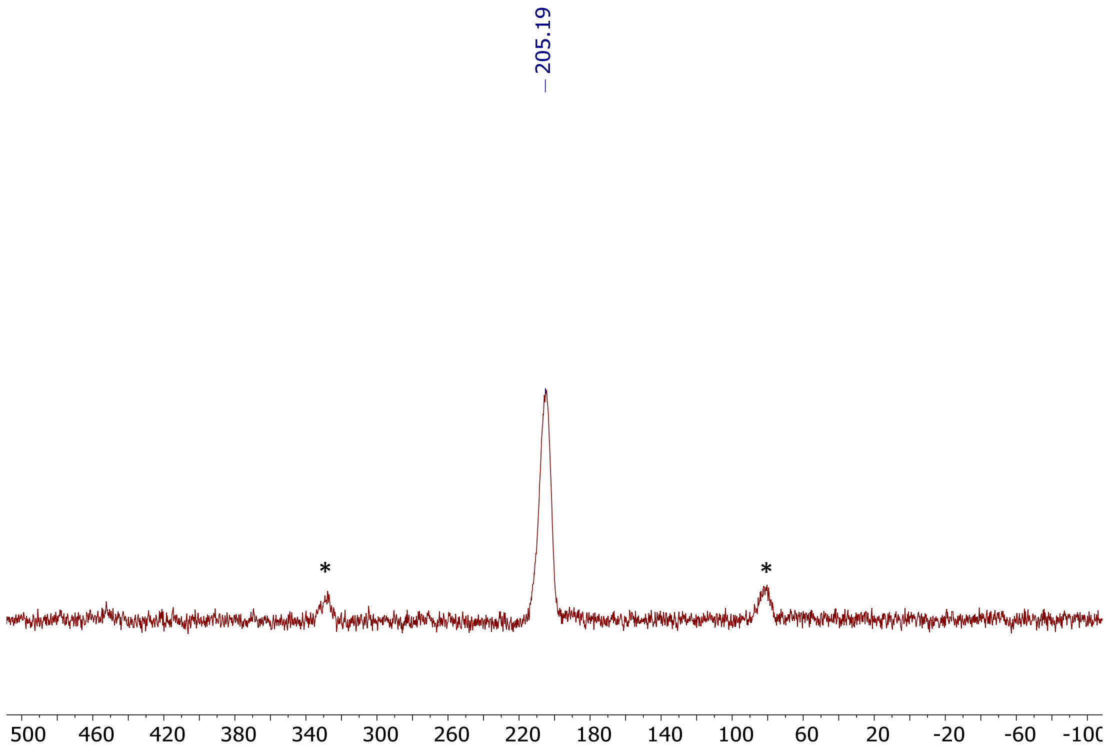

Figure S13. $^{31}P\{^1H\}$ *CPMAS SSNMR spectrum (20 KHz, 162 MHz, 298 K) of crystalline* ***[2][BAr$^F{}_4$]****.* indicates spinning side bands.*

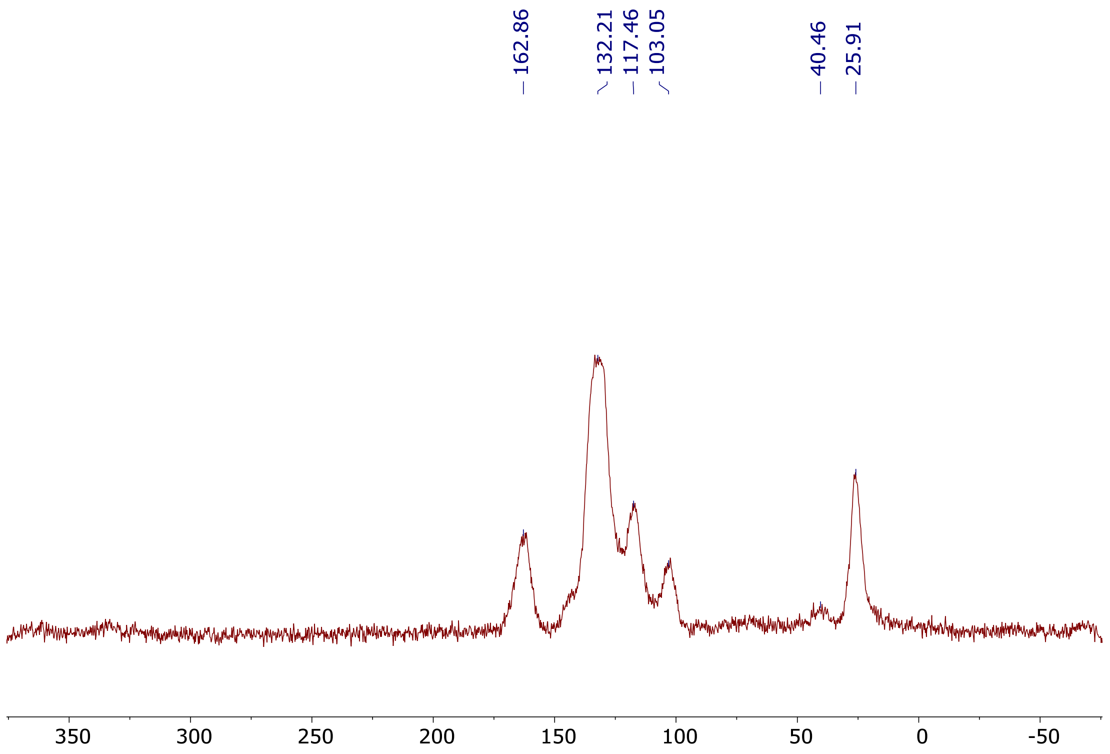

Figure S14. $^{13}C\{^1H\}$ *CPMAS NMR spectrum (20 KHz, 100 MHz, 298 K) of crystalline* ***[2][BAr$^F{}_4$]****.*

[3][BArF4]

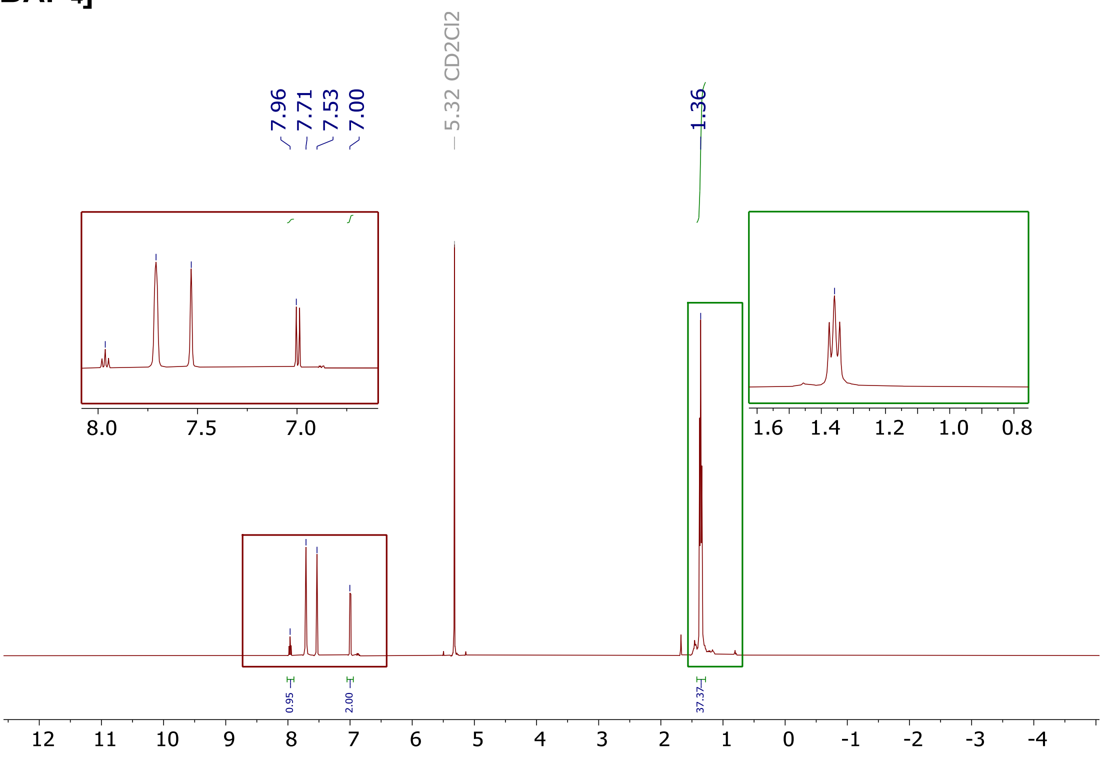

*Figure S15. ^{1}H NMR spectrum (CD_2Cl_2, 500 MHz, 298K) of crystalline **[3][BAr^F$_4$]**.*

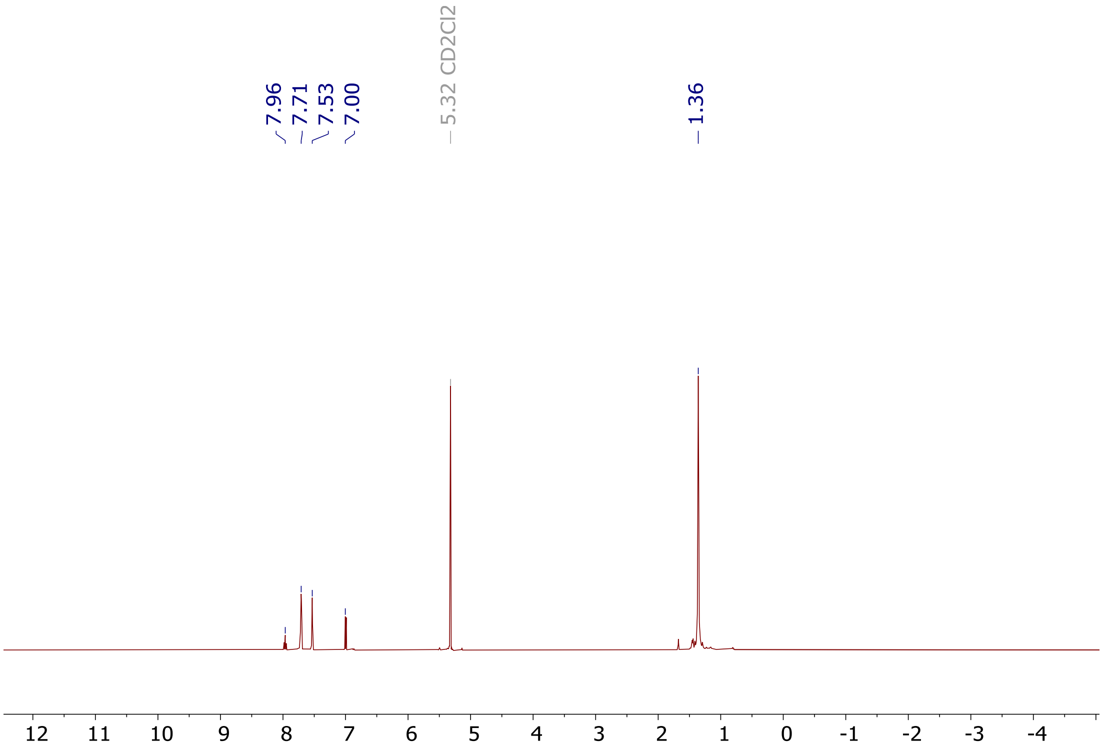

*Figure S16. ^{1}H{^{31}P} NMR spectrum (CD_2Cl_2, 500 MHz, 298K) of crystalline **[3][BAr^F$_4$]**.*

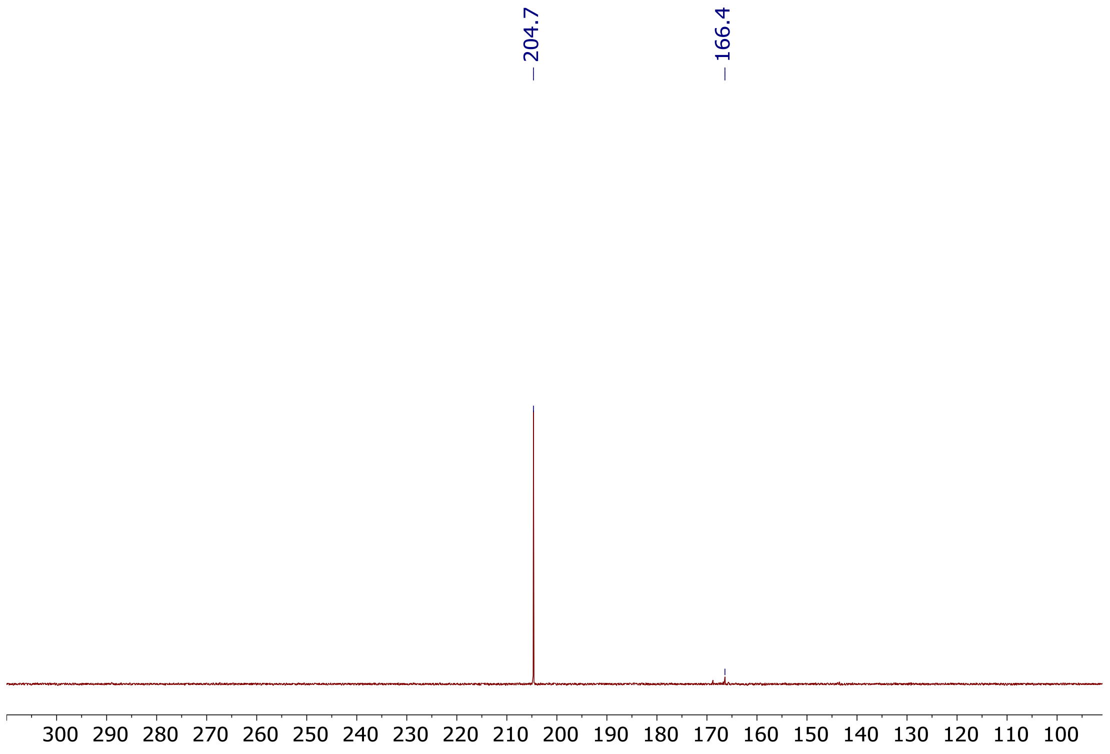

*Figure S17. $^{31}P\{^1H\}$ NMR spectrum (CD_2Cl_2, 202 MHz, 298K) of crystalline **[3][BAr$^F{}_4$]**. Peak 166 corresponds to an unidentified impurity.*

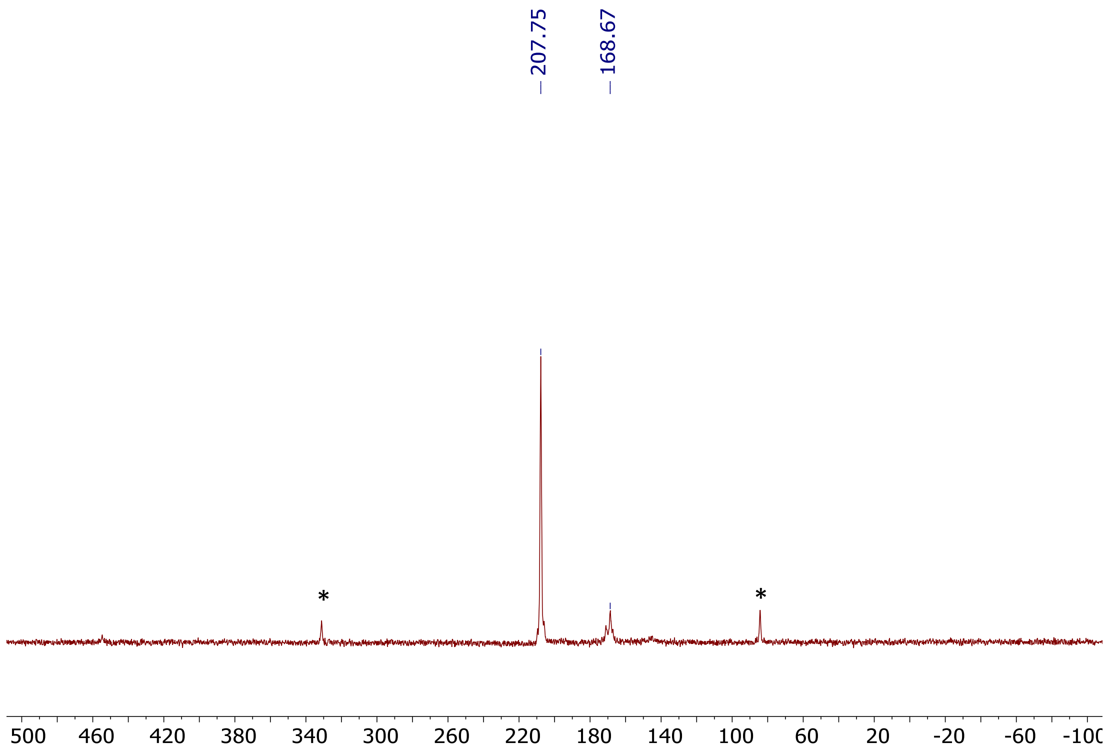

*Figure S18. $^{31}P\{^1H\}$ CPMAS SSNMR spectrum (20 KHz, 162 MHz, 298 K) of crystalline **[3][BAr$^F{}_4$]**.* indicates spinning side bands. Peak 168 corresponds to an unknown trace impurity in the CO gas. The peak at 168 is an unidentified trace impurity. Due to the nature of cross-polarization during acquisition the relative intensities of these peaks are not quantitative (see solution data above).*

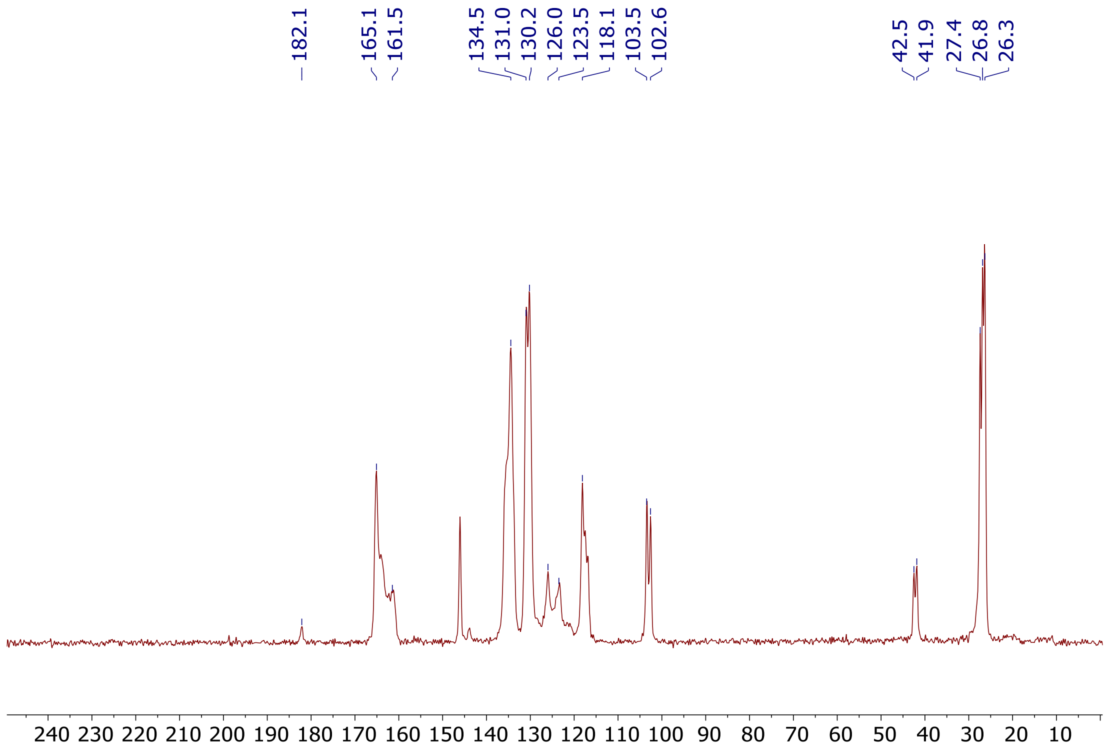

Figure S19. $^{13}C\{^{1}H\}$ *CPMAS NMR spectrum (20 KHz, 100 MHz, 298 K) of crystalline* ***[3][BAr^F$_4$]***.

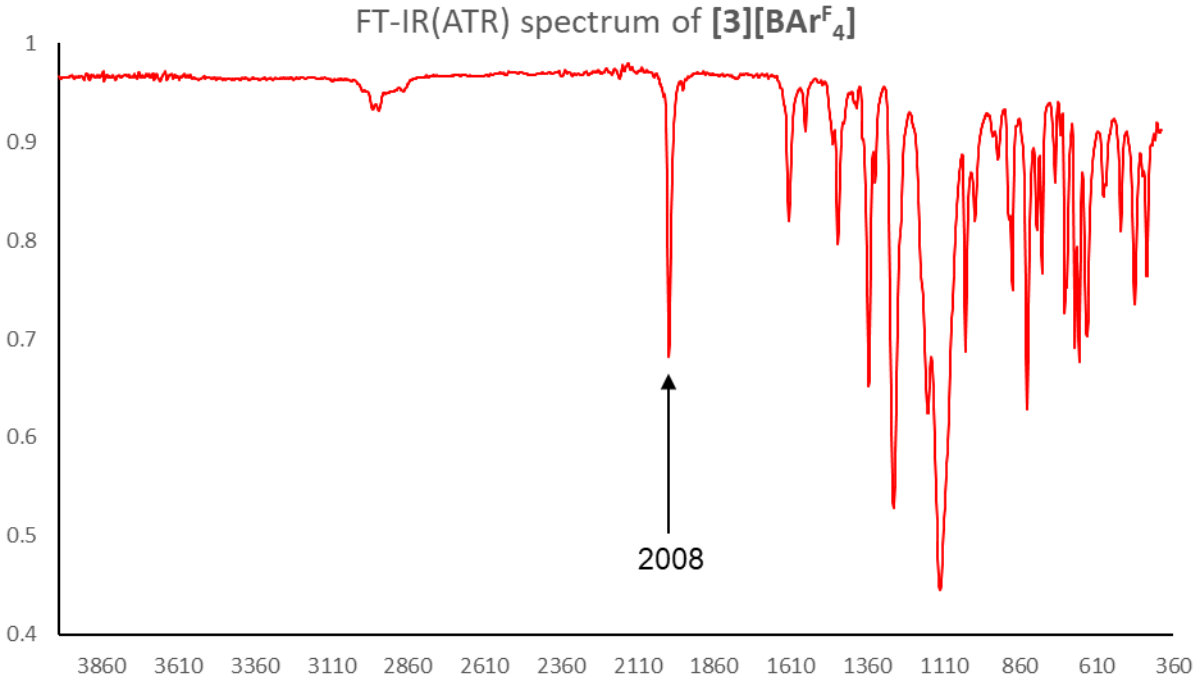

Figure S20. FT-IR(ATR) spectrum of crystalline ***[3][BAr^F$_4$]***.

[4][BAr^F$_4$]

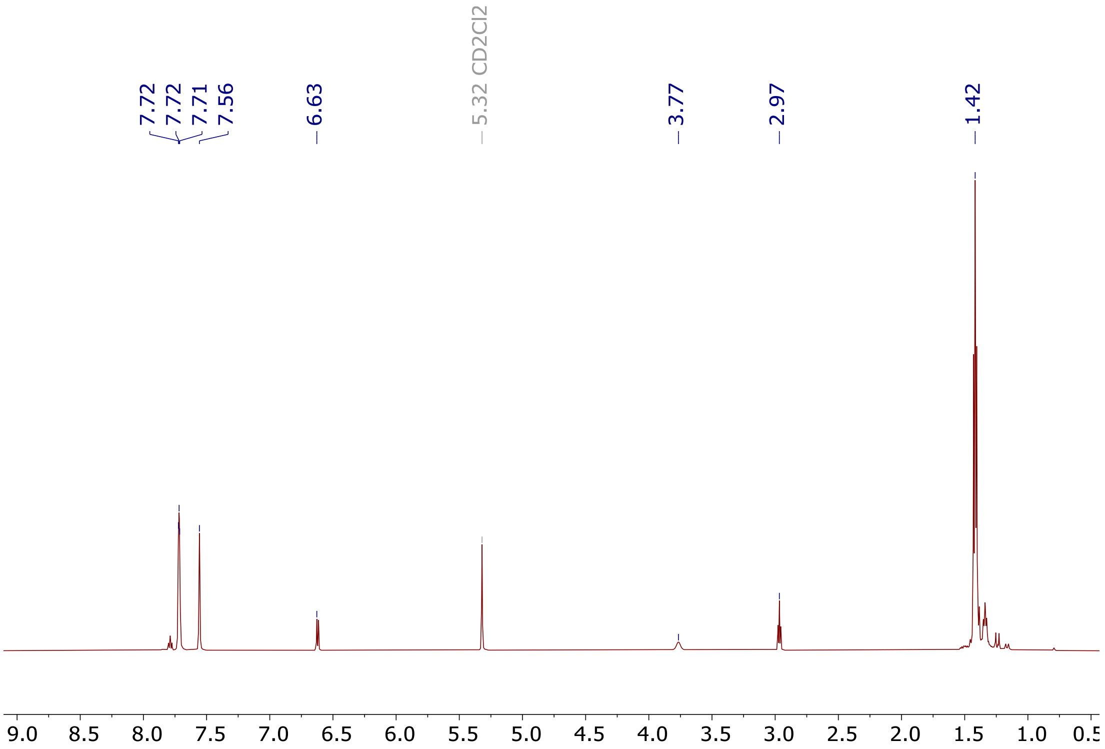

*Figure S21. ^{1}H NMR spectrum (CD_2Cl_2, 600 MHz, 298K) of crystalline **[4][BAr^F$_4$]**.*

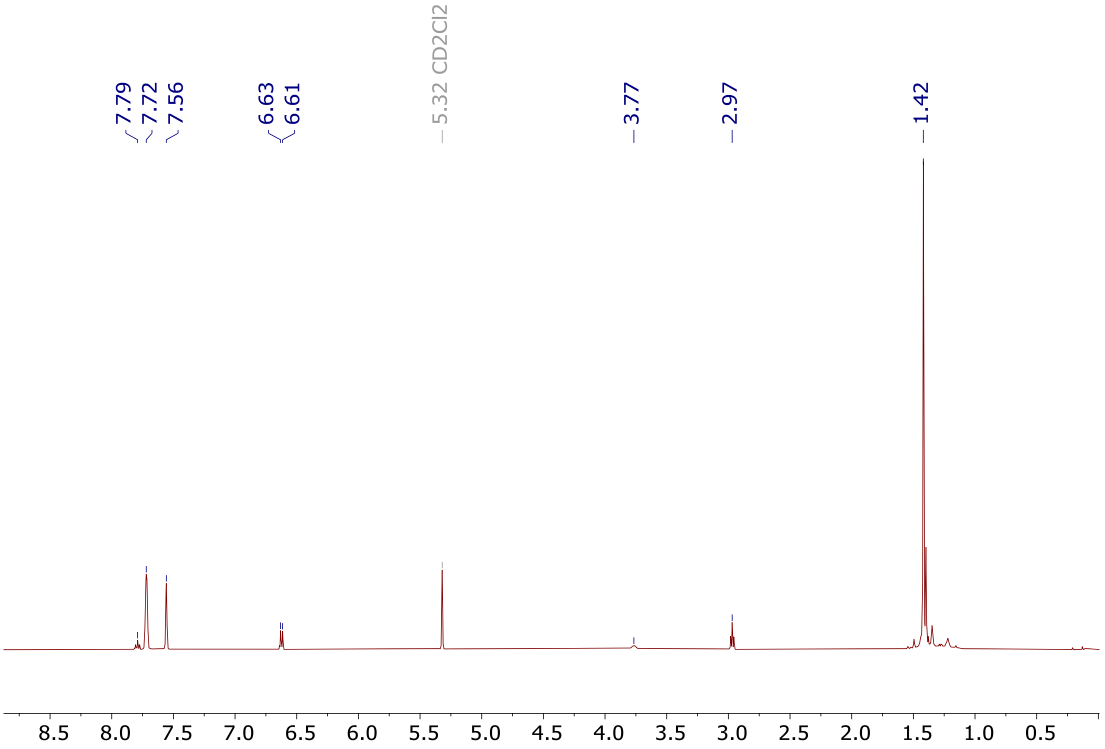

*Figure S22. ^{1}H{^{31}P} NMR spectrum (CD_2Cl_2, 500 MHz, 298K) of crystalline **[4][BAr^F$_4$]**.*

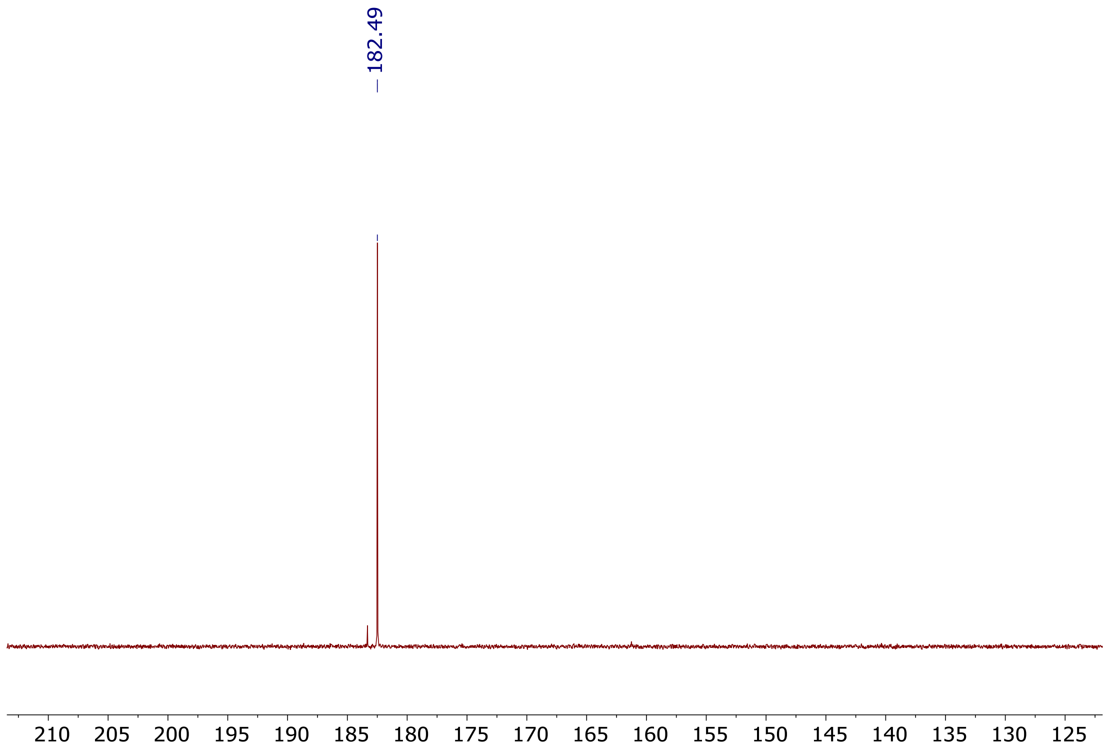

Figure S23. $^{31}P\{^{1}H\}$ NMR spectrum (CD_2Cl_2, 202 MHz, 298K) of crystalline ***[4][BAr^F$_4$]***.

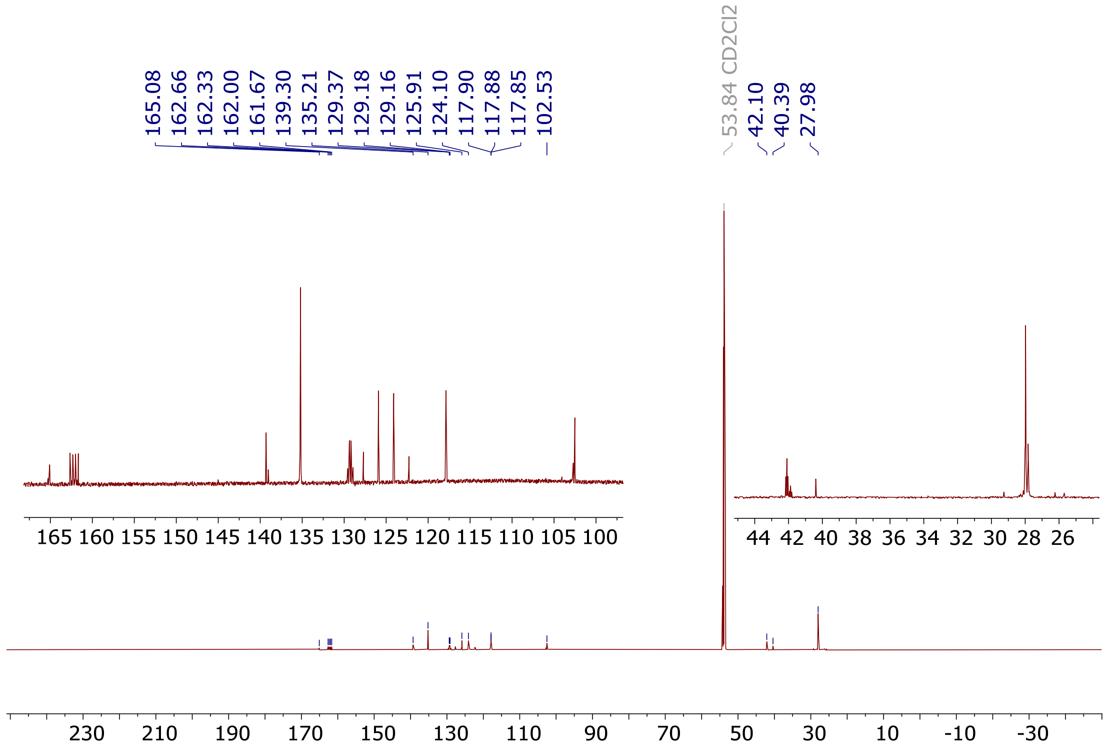

Figure S24. $^{13}C\{^{1}H\}$ NMR spectrum (CD_2Cl_2, 150 MHz, 298 K) of crystalline ***[4][BAr^F$_4$]***.

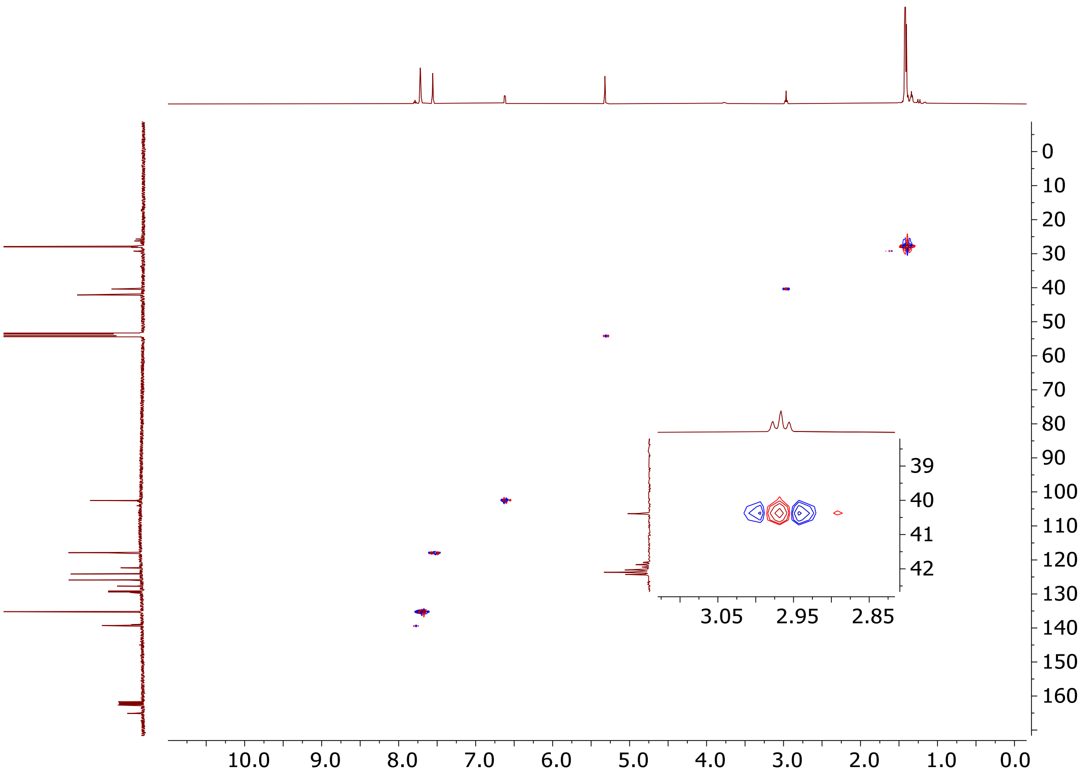

*Figure S25. ^1H-^{13}C HSQC spectrum (CD_2Cl_2,600/150 MHz, 298 K) of crystalline **[4][BAr^F$_4$]**. Inset shows the $^1J_{C-H}$ correlation between the methylamine ligand protons and carbon.*

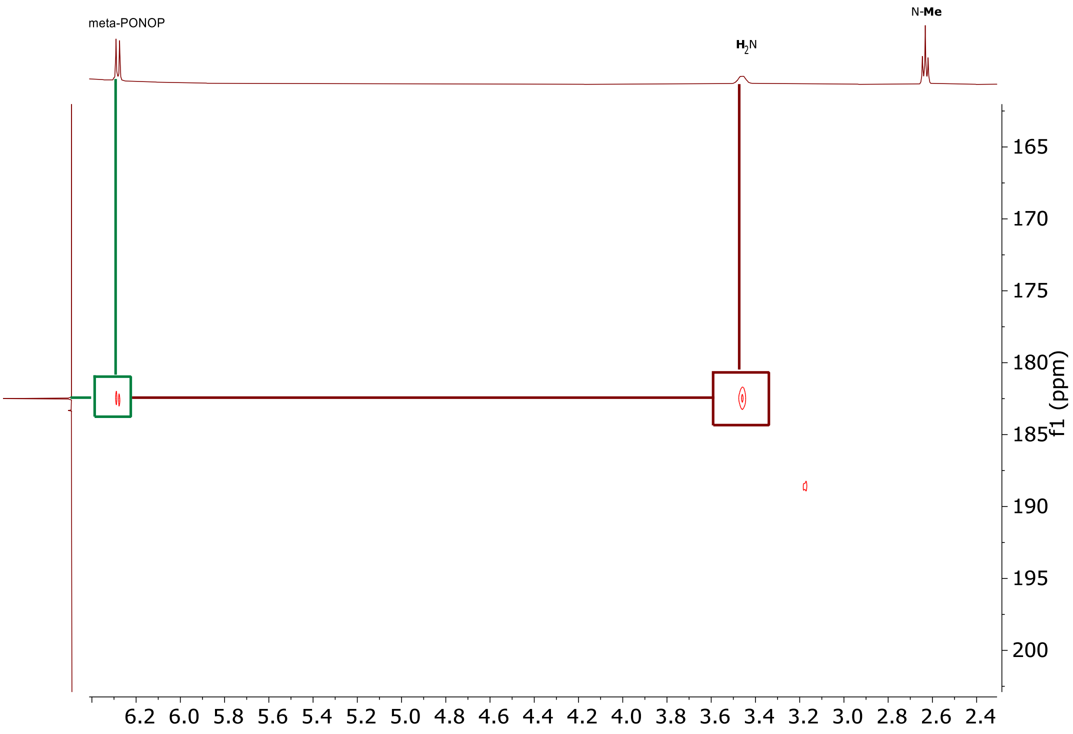

*Figure S26. ^1H-^{31}P HMBC spectrum (FC_6H_5, 500/202 MHz, 298 K) of crystalline **[4][BAr^F$_4$]** showing correlation between the meta-PONOP proton signal and phosphorus signal as well as the correlation between the **H_2**NMe signal and the phosphorus signal. Noteworthy is the lack of correlation between the H_2N**Me** triplet at 2.63 ppm and the phosphorus resonance confirming coordination of the methylamine ligand through the nitrogen.*

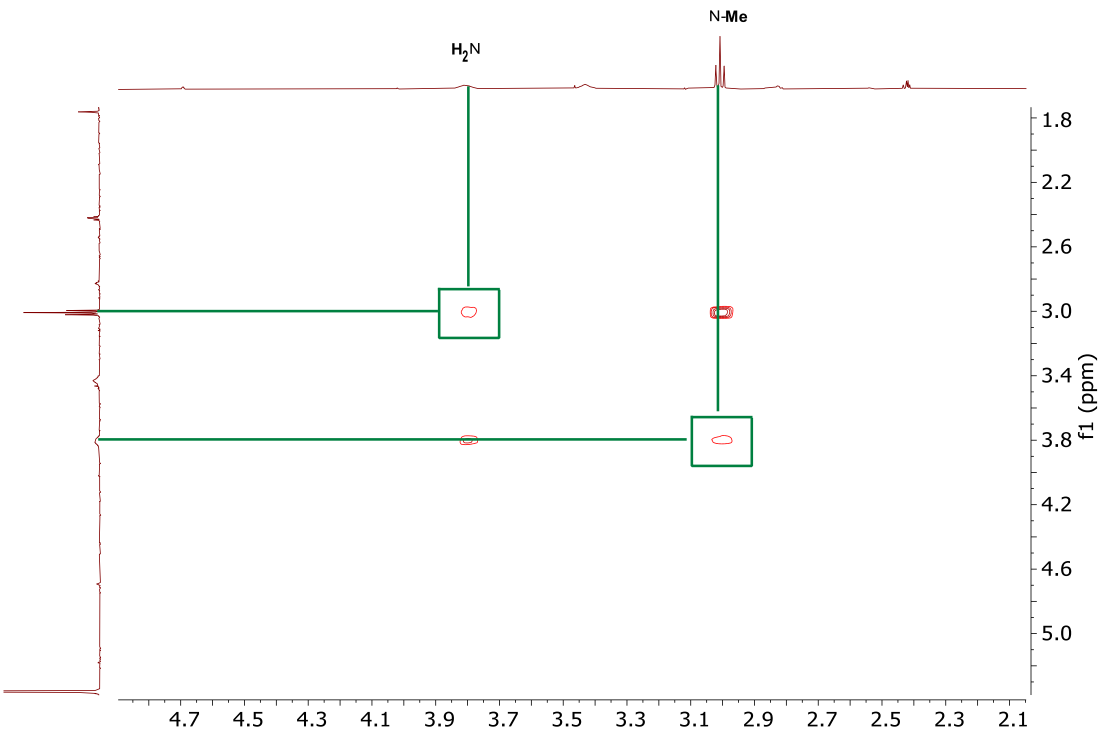

Figure S27. ^{1}H-^{1}H *COSY NMR spectrum (*CD_2Cl_2*, 500/500 MHz, 298K) of crystalline* ***[4][BAr^F_4]***.

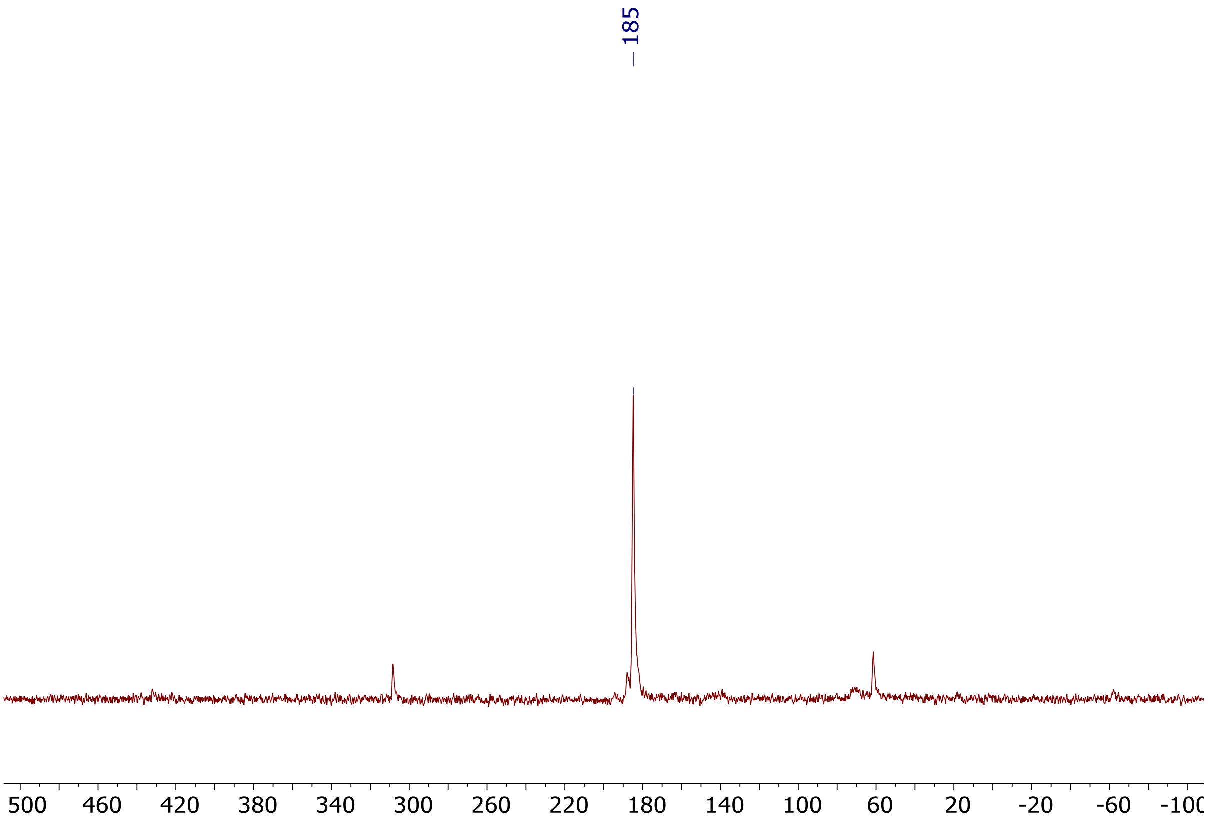

Figure S28. $^{31}P\{^{1}H\}$ *CPMAS SSNMR spectrum (20 KHz, 162 MHz, 298 K) of crystalline* ***[4][BAr^F_4]****.* indicates spinning side bands.*

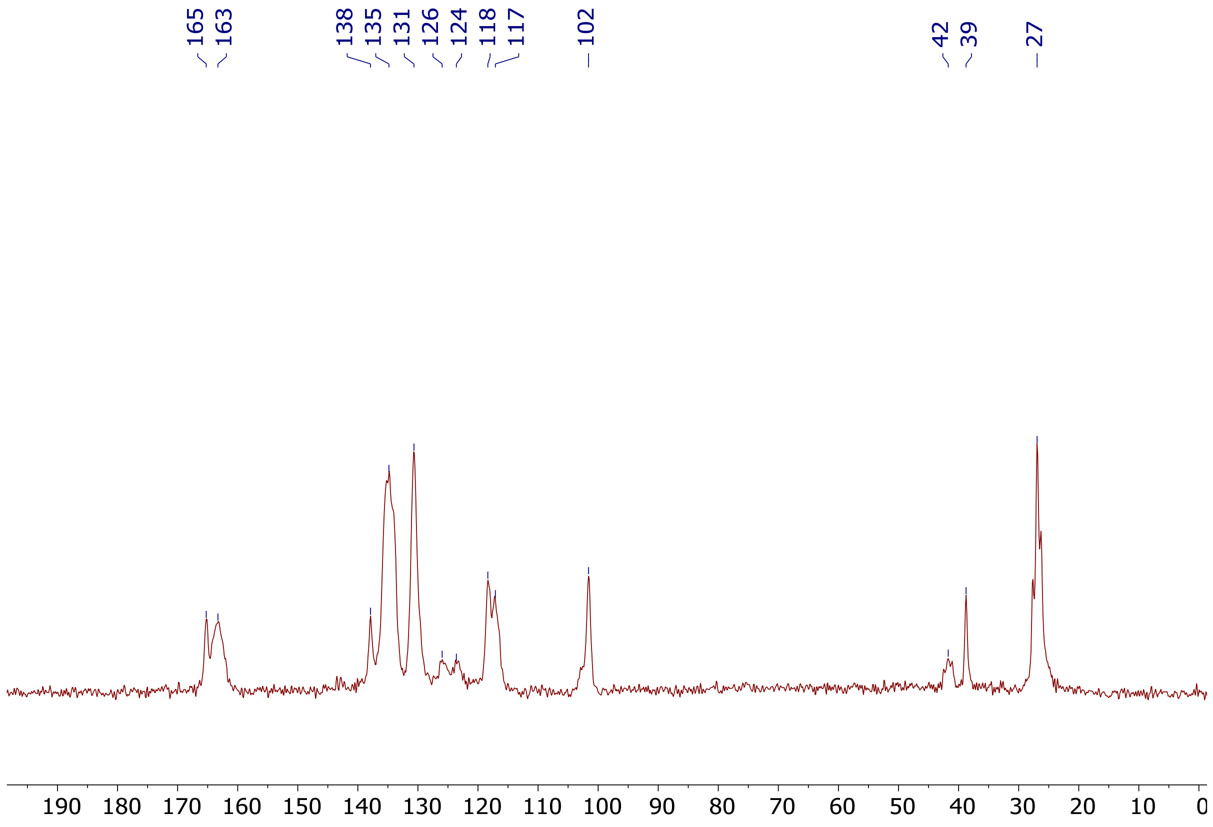

Figure S29. $^{13}C\{^{1}H\}$ CPMAS NMR spectrum (20 KHz, 100 MHz, 298 K) of crystalline ***[4][BAr$^{F}_{4}$]****.*

Visual representation of the single-crystal to single-crystal reaction of **[1][BAr^F$_4$]** with CO over time.

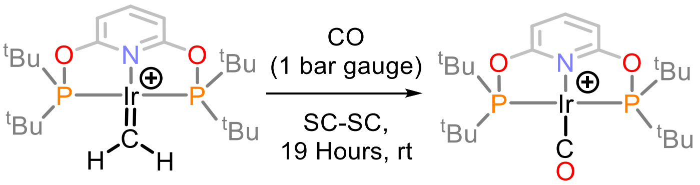

[1] **[4]**

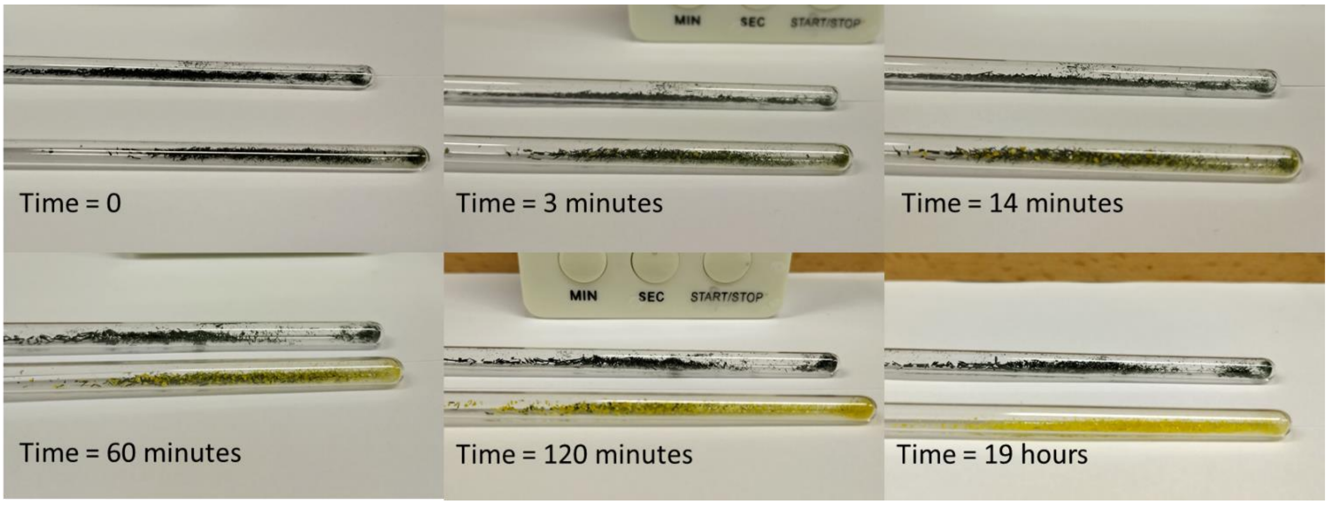

*Figure S30. Visual representation time lapse of the SC-SC reaction of **[1][BAr^F$_4$]** with CO to give **[4][BAr^F$_4$]**. Top NMR tube corresponds to reference sample of **[1][BAr^F$_4$]**; bottom NMR tube corresponds to SC-SC reaction.*

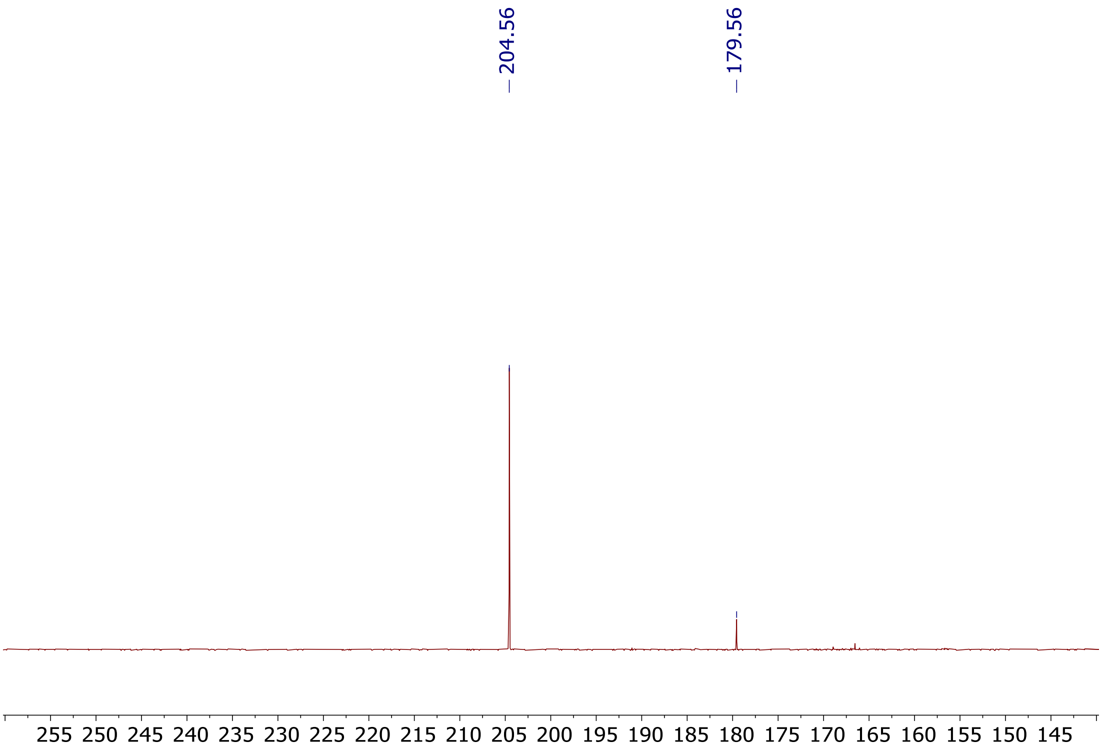

*Figure S31. $^{31}P\{^1H\}$ NMR spectrum (C_6H_5F, 500 MHz, 243 K) of crystalline **[1][BAr^F$_4$]** after exposure to CO gas (2 bar absolute) for 120 minutes.*

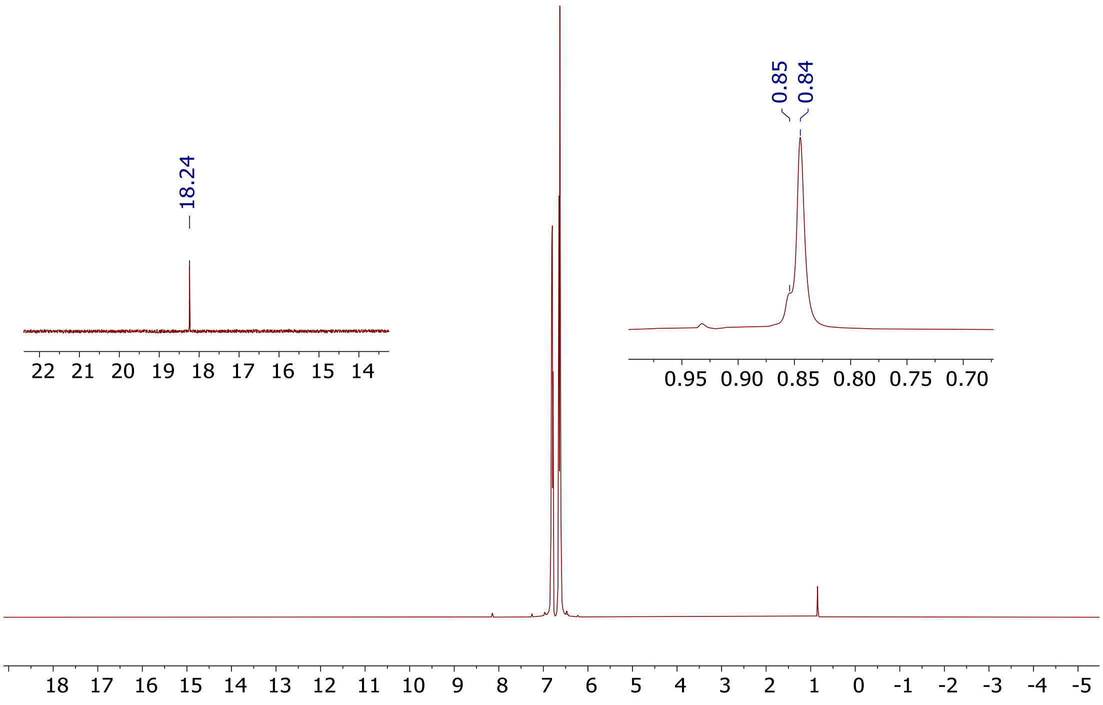

Figure S32. $^{1}H\{^{31}P\}$ NMR spectrum (C_6H_5F, 500 MHz, 243 K) of crystalline ***[1][BAr$^F_4$]*** *after exposure to CO gas (2 bar absolute) for 120 minutes.*

Discussion:

The SC-SC reaction was stopped after 120 minutes by removing the CO atmosphere by vacuum. The atmosphere was replaced with argon and the crystals dissolved in fluorobenzene to show the ratio of **[1][BAr$^F_4$]** to **[3][BAr$^F_4$]**. **[1][BAr$^F_4$]** was still observed by $^{31}P\{^{1}H\}$ and $^{1}H\{^{31}P\}$ NMR spectroscopy, in agreement with the observation of green crystals still present amongst the bulk sample as seen in Figure S28.

NMR analysis of the reaction between **[1][BAr$^{F}_{4}$]** and NH_3.

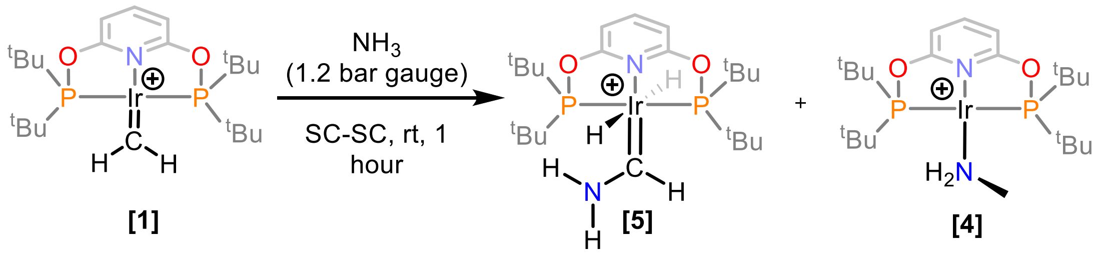

Analysis of the SC-SC reaction of **[1][BAr$^{F}_{4}$]** with ammonia after 1 hour by NMR spectroscopy reveals the presence of the kinetic product **[5][BAr$^{F}_{4}$]**. NMR spectroscopic assignments of the resonances associated with **[5][BAr$^{F}_{4}$]** are shown in the following figures.

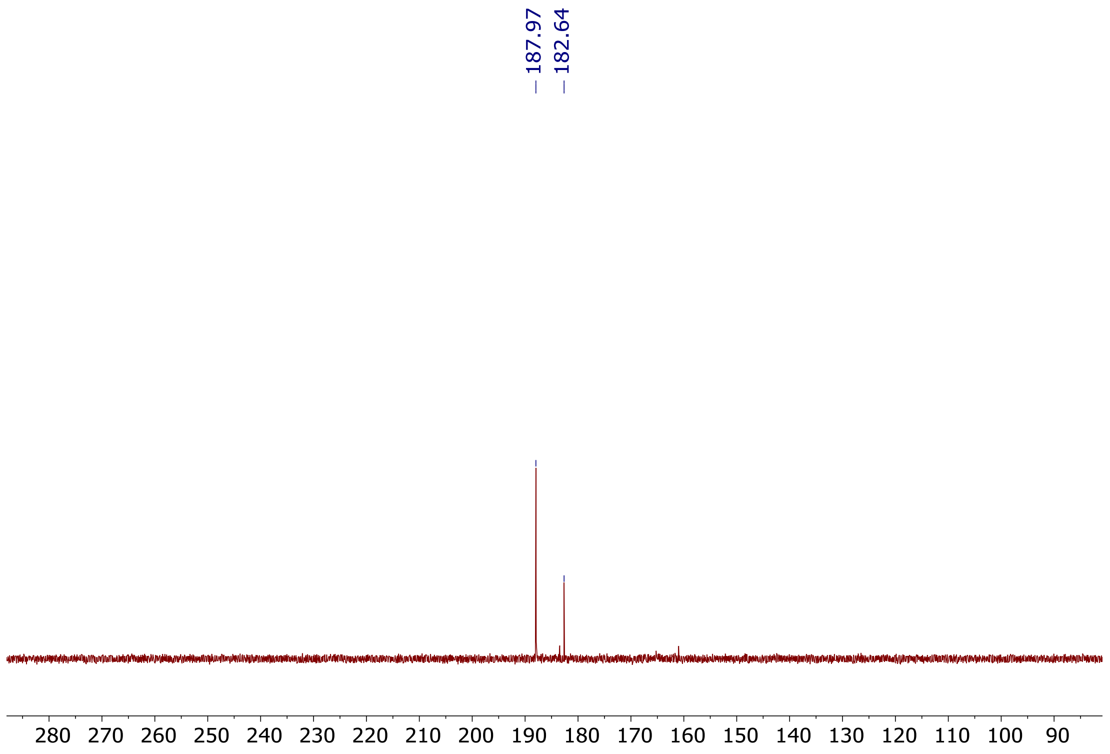

*Figure S33. $^{31}P\{^{1}H\}$ NMR spectrum (C_6H_5F, 202 MHz, 298 K) of the SC-SC reaction between **[1][BAr$^{F}_{4}$]** and NH_3 after 1 hour showing two compounds one which being the expected product **[4][BAr$^{F}_{4}$]** (δ 182).*

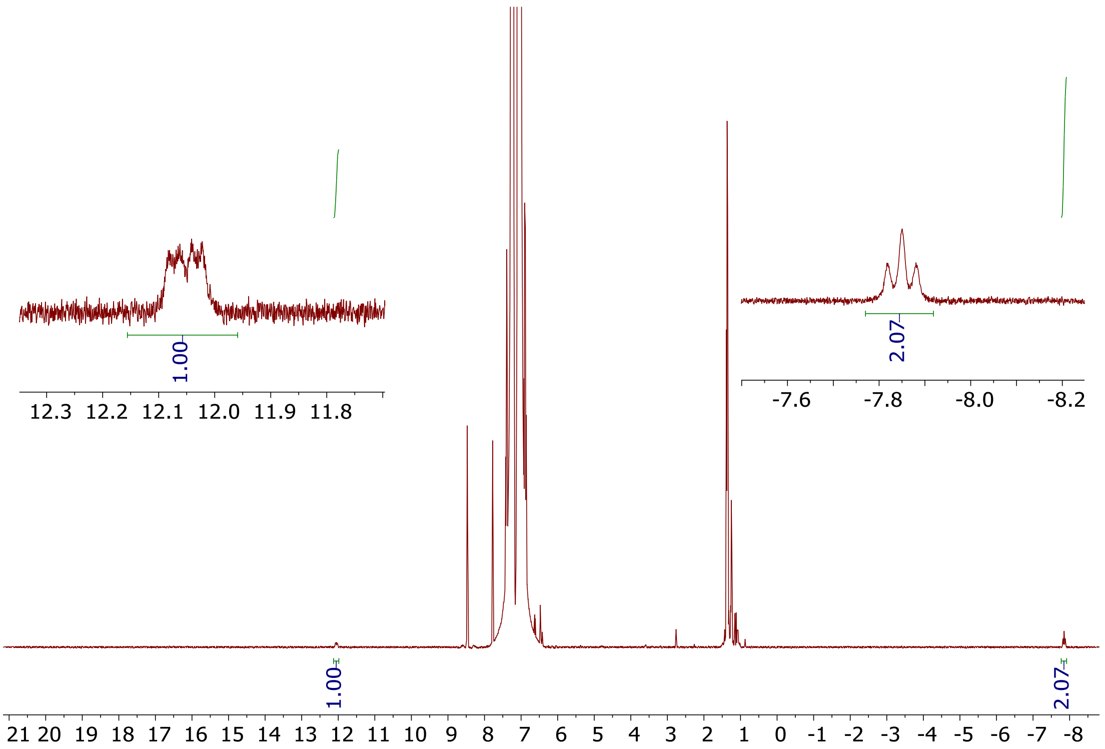

Figure S34. [1]*H NMR spectrum (C_6H_5F, 500 MHz, 298 K) of the SC-SC reaction between* ***[1][BAr^F$_4$]*** *and NH_3 after 1 hour showing the downfield multiplet with integral 1 H and the upfield resonance corresponding to the dihydrogen protons with integral 2 H.*

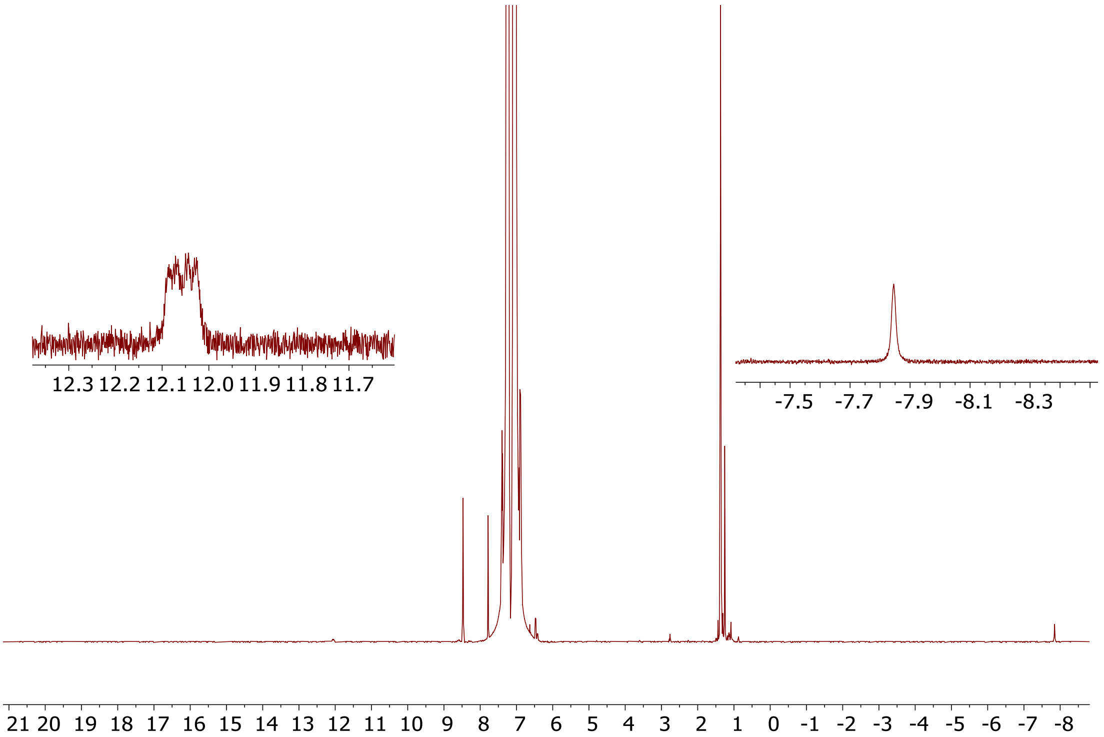

Figure S35. $^1H\{^{31}P\}$ *NMR spectrum (C_6H_5F, 500 MHz, 298 K) of the SC-SC reaction between* ***[1][BAr^F$_4$]*** *and NH_3 after 1 hour showing the collapse of the dihydride resonance into a singlet. However, the downfield resonance of the aminocarbene does not lose multiplicity suggesting some degree N-C double bond character giving rise to a doublet of doublets from the non-equivalent NH protons (J_{H-H} = 20 Hz and J_{H-H} = 9.5 Hz).*

Solid-State Calculations

All static Kohn-Sham DFT calculations were performed on periodic models of the studied iridium complexes, employing the Gaussian Plane Wave (GPW) formalism as implemented in the QUICKSTEP[S3] module within the CP2K program suite (Version 2023.1).[S4] Molecularly optimised basis sets of double-ζ quality plus polarization in their short-range variant (DZVP-MOLOPT-SR-GTH)[S5] were used for all atomic species. The interaction between the core electrons and the valence shell (Ir: 17, B: 3, C: 4, N: 5, O: 6, P: 5, F: 7, H: 1 electrons) was described by Goedecker-Teter-Hutter (GTH) pseudo potentials.[S6-S8] The generalized gradient approximation (GGA) to the exchange-correlation functional according to Perdew-Burke-Ernzerhof (PBE)[S9] was used in combination with Grimme's D3 correction for dispersion interactions.[S10] The auxiliary plane wave basis set was truncated at a cutoff of 500 Ry. The maximum force convergence criterion was set to 10^{-4} Eh·Bohr^{-1}, whilst default values were used for the remaining criteria. The convergence criterion for the self-consistent field (SCF) accuracy was set to 10^{-7} Eh and 10^{-8} Eh for geometry optimizations. The Brillouin zone was sampled using the Γ-point. Initial coordinates for the Ir-methylene complex, **[1][BAr$^F_4$]**, were obtained from the experimental crystallographic data. Periodic boundary conditions (PBC) were applied throughout in combination with fixed unit cell parameters obtained from experiment.

Reactivity studies in the solid state were centred one of the cations within the unit cell, all of which are equivalent under the crystal symmetry (Z = 8; Z' = 1). The reaction was modelled in both directions, i.e. starting from the crystal structure of **[1][BAr$^F_4$]** and starting from the crystal structure of **[4][BAr$^F_4$]**. Only minor differences in the computed geometries and energies were seen (see Figures S50 and S51). Transition state calculations were performed using dimer method with tighter convergence criteria (maximum force 10^{-4} Hartree·Bohr^{-1} and SCF 10^{-7} Eh).[S11] Optimized stationary points were characterised by analysis of their numerical second derivatives with a displacement of 0.01 Bohr, where only the atoms of the reacting cation were included in the analysis. Minima and transition states have none or exactly one imaginary eigenvalue, respectively.A[S12] A partial Hessian vibration analysis (PHVA) analysis was performed to compute the thermodynamic data for the reactions using the TAMKIN tool.[S13] Test calculations showed similar results were obtained when all atoms were included in the Hessian calculation and the subsequent PHVA analysis, as reported by Ghysels and co-workers (see Table S1).[S14]

Molecular Calculations

The solid-state cation and ion-pair geometries were extracted from the fully optimised periodic DFT structure and single point energy calculations and full geometry optimizations were performed as required in the vacuum using the Gaussian 16 (Revision A.03) program package.[S15]These employed the PBE functional with Grimme's D3 dispersion correction.[S9, S16] Stuttgart-Dresden (SDD)[S17] relativistic effective core potentials (ECP) in combination with the associated basis sets were utilized to describe Ir and P, with polarization functions added for P (ζ = 0.387).[S18] 6-31G(d,p) basis sets[S19, S20] were used for all remaining atoms. Extended wavefunction (.wfx) files suitable for bonding analysis were generated using single point calculations. Reaction profiles in the vacuum and including a correction for solvation (fluorobenzene, ε = 5.42) are provided in Figures S48 and S49.[S21, S22]

Independent gradient model calculations were performed with Multiwfn[S23] with the Hirshfeld partitioning scheme (IGMH method).[S24] Surfaces were visualized with VMD[S25] QTAIM (Quantum Theory of Atoms in Molecules)[S26] calculations employed the AIMALL package.[S27] NCI calculations were performed using the NCIPLOT program[S28, S29] and employed promolecular electron densities. Wiberg bond indices and natural atomic charges were

computed with the NBO 6.0 program.[S30] The molecular electrostatic potential map for the extracted cation was built with Gaussview6.0.[S31]

NMR chemical shift calculations were performed within the GIAO framework using ADF 2023.[S32, S33] with the B3LYP functional[S34] and Slater-type basis sets of double-ζ (DZP) quality.[S35] Relativistic effects were treated by the 2-component zeroth-order regular approximation (ZORA).[S36]

Electronic Structure Analyses.

[1][BAr$^F_4$]

All analyses preformed on the Structure of **[1]**$^+$ extracted from the solid-state periodic DFT optimisation.

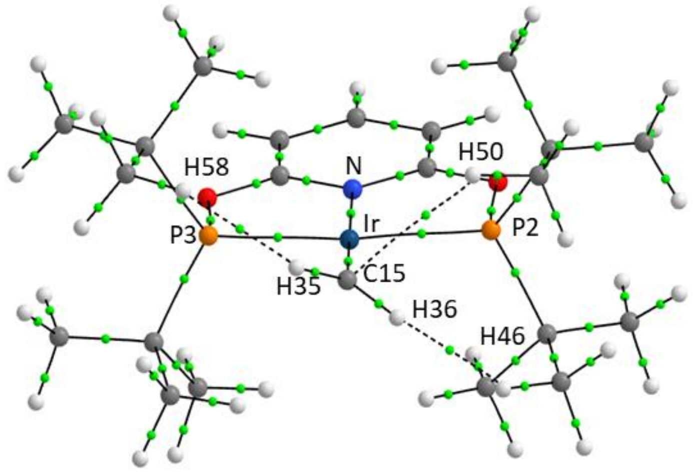

BCP	Distance	ρ	ε	$\nabla^2\rho$	H	DI	Atom	q (e)
Ir−C15	1.887	0.1858	0.0720	+0.2814	−0.1099	1.7333	Ir	+0.2703
Ir−N	2.102	0.1009	0.3574	+0.4278	−0.0228	0.6736	N	−1.2042
Ir−P2	2.285	0.1147	0.0663	+0.0975	−0.0551	0.9814	P2	+1.5737
Ir−P3	2.294	0.1128	0.0792	+0.0983	−0.0533	0.9651	P3	+1.5560
C15−H35	1.100	0.2758	0.0421	−0.9574	−0.2786	0.9430	C15	−0.2098
C15−H36	1.100	0.2761	0.0423	-0.9599	−0.2791	0.9430	H35	+0.0196
H35···H58	2.166	0.0074	0.0949	+0.0263	+0.0014	0.0217	H36	+0.0195
H36···H46	2.267	0.0061	0.1766	+0.0223	+0.0013	0.0173	H46	+0.0067
C15···H50	2.679	0.0081	1.3525	+0.0250	+0.0013	0.0263	H50	+0.0147

*Figure S36. QTAIM molecular graph for **[1]**$^+$ with bond critical points (BCP) shown as green spheres. Structure of **[1]**$^+$ extracted from the solid-state periodic DFT optimised structure. Table shows selected associated QTAIM BCP parameters (au), distances (Å) and atomic charges. Note the atom labelling used here differs from that in the main text.*

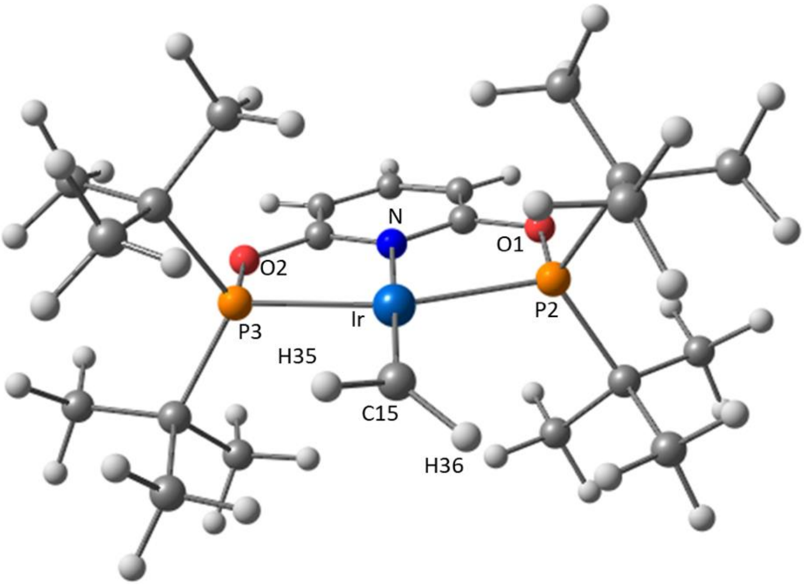

Wiberg bond indices	
Ir−C15	1.4012
Ir−N	0.2838
Ir−P2	0.5550
Ir−P3	1.5454
C15−H35	0.9289
C15−H36	0.9287
**Natural atomic charges**	
Ir	−0.1519
C15	−0.3875
N	−0.5892
P2	1.5064
P3	1.4927
H35	0.2043
H36	0.2065

Figure S37. Structure and tabulated selected Wiberg bond indices and natural atomic charges for ***[1]***$^{+}$.

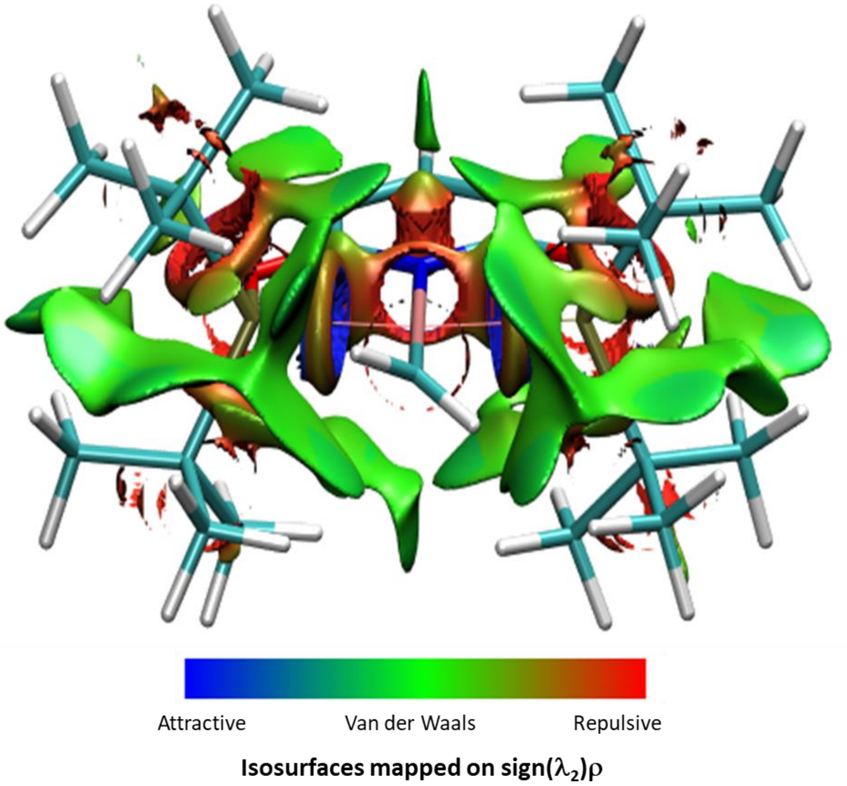

Figure S38. NCI plot for ***[1]***$^{+\cdot}$ *Sign(λ_2)ρ–coloured isosurfaces are plotted with s = 0.45 a.u and $-0.07 < \rho < 0.07$ a.u.*

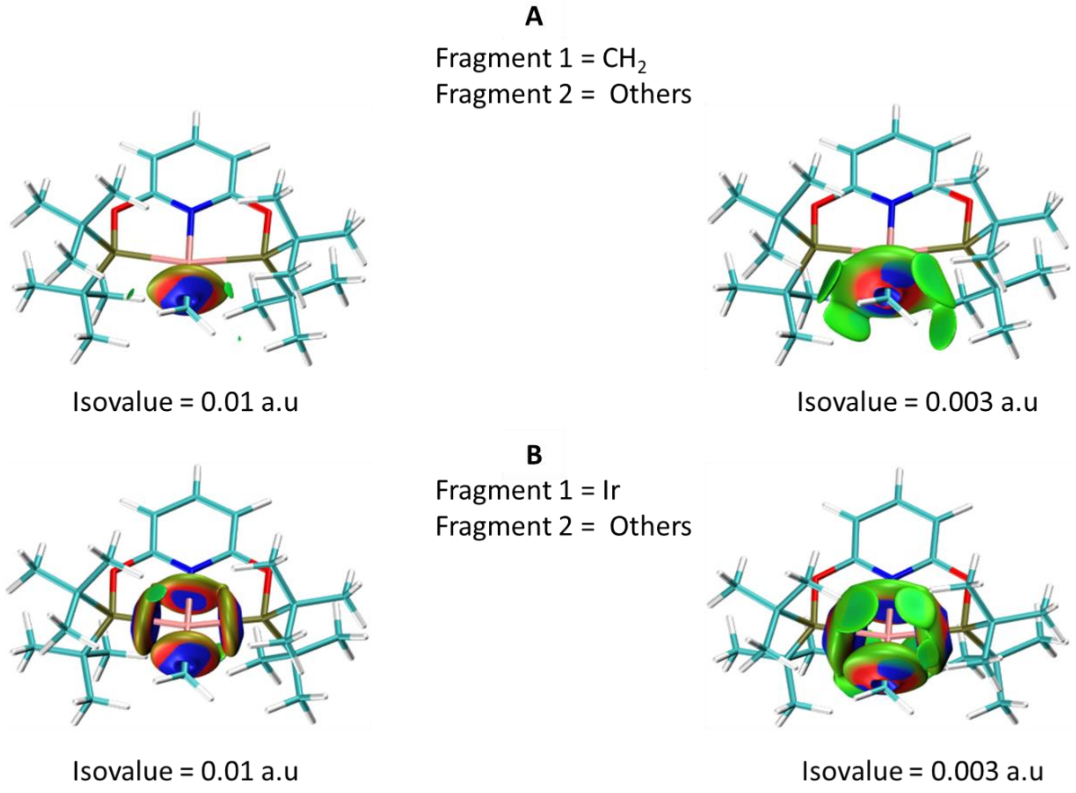

*Figure S39. IGMH plots for **[1]⁺**. **A**. CH_2 group and all other atoms are defined as separate fragments; **B**. Ir and all other atoms are defined as separate fragments. Sign(λ_2)ρ–coloured isosurfaces are plotted with δG^{inter} = 0.01 a.u and 0.003 a.u. respectively.*

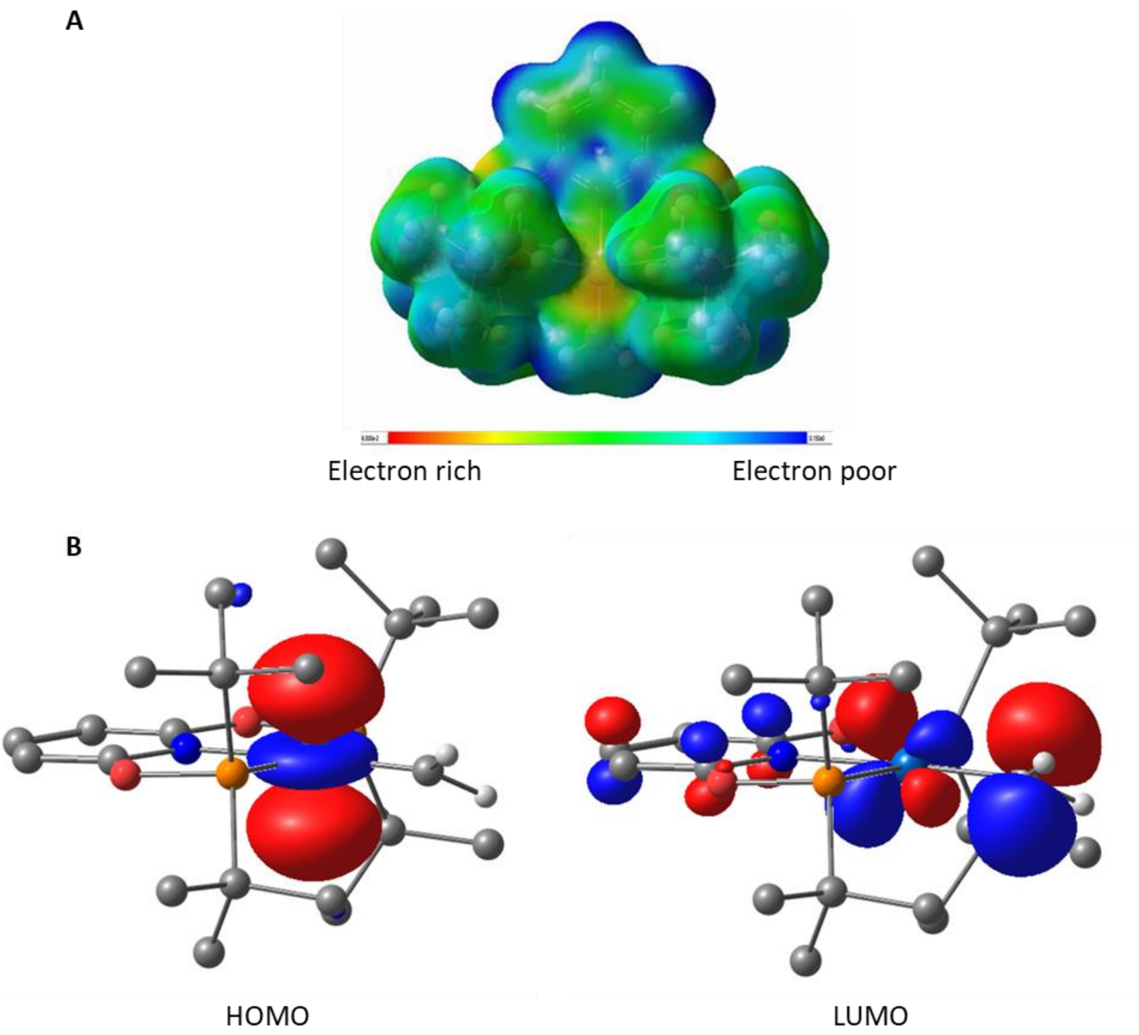

Figure S40. A. Electrostatic potential map for ***[1]***$^{+}$*.* ***B****. HOMO and LUMO of* ***[1]***$^{+}$ *(isosurface value= 0.0518 a.u.; non-alkylidene hydrogens omitted for clarity).*

[(^{t}Bu-PONOP)Ir(Me)(H)][BAr$^F_4$]

All analyses preformed on the structure of [(^{t}Bu-PONOP)Ir(Me)(H)]$^+$ extracted from the solid-state periodic DFT optimisation.

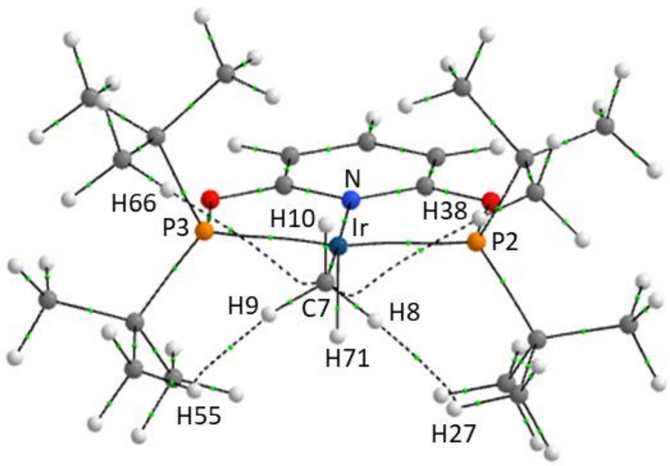

BCP	Distance	ρ	ε	$\nabla^2\rho$	H	DI	Atom	q (e)
Ir−C7		0.1263	0.0143	+0.1084	−0.0569	0.9815	Ir	+0.3526
Ir−N		0.0993	0.1491	+0.4437	−0.0221	0.7009	N	−1.2031
Ir−P2		0.1136	0.1031	+0.1051	−0.0537	0.9792	P2	+1.5944
Ir−P3		0.1132	0.1009	+0.1047	−0.0534	0.9770	P3	+1.5854
Ir−H71		0.1759	0.0243	+0.0001	−0.1120	1.0250	H8	−0.0129
C7−H8		0.2702	0.0081	−0.8853	−0.2698	0.9545	C7	−0.2057
C7−H9		0.2708	0.0083	−0.8894	−0.2709	0.9528	H9	−0.0135
C7−H10		0.2603	0.0130	−0.8111	−0.2538	0.9442	H10	−0.0227
H9···H55		0.0081	0.0749	+0.0300	+0.0017	0.0197	H55	+0.0205
C7···H66		0.0044	0.2514	+0.0152	+0.0009	0.0128	H66	+0.0158
C7···H38		0.0062	0.7291	+0.0224	+0.0013	0.0180	H38	+0.0165
H8···H27		0.0059	0.3464	+0.0215	+0.0013	0.0133	H27	+0.0196
–		–	–	–	–	–	H71	−0.0053

Figure S41. QTAIM molecular graph for [(^{t}Bu-PONOP)Ir(Me)(H)]$^+$ with bond critical points (BCP) shown as green spheres. Structure of [(^{t}Bu-PONOP)Ir(Me)(H)]$^+$ extracted from the solid-state periodic DFT optimised structure. Table shows selected associated QTAIM BCP parameters (au), distances (Å) and atomic charges. Note the atom labelling used here differs from that in the main text.

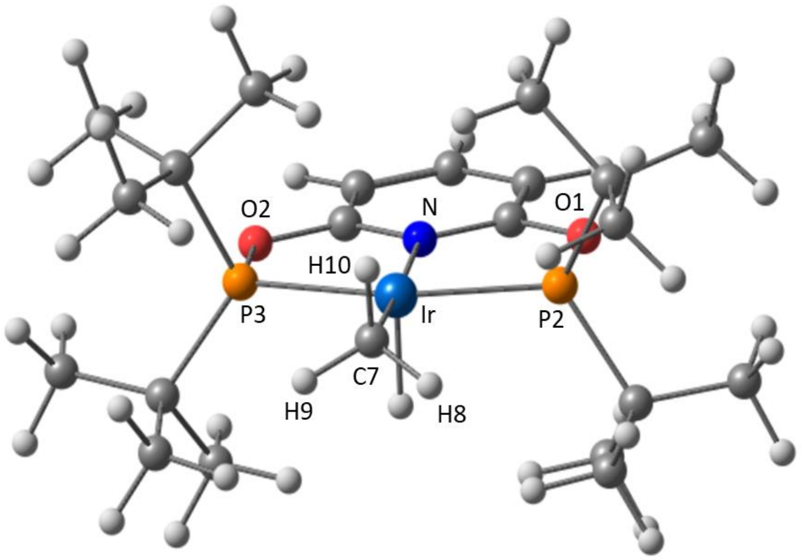

Wiberg bond indices	
Ir−C7	0.6458
Ir−N	0.2809
Ir−P2	0.5166
Ir−P3	0.5154
Ir−H71	0.7713
C7−H8	0.9269
C7−H9	0.9269
C7−H10	0.9232
**Natural atomic charges**	
Ir	−0.0715
C7	−0.9572
N	−0.5584
P2	1.5116
P3	1.5094
H71	0.1958
H8	0.2403
H9	0.2390
H10	0.2322

Figure S42. Structure and tabulated selected Wiberg bond indices and natural atomic charges for [(^{t}Bu-PONOP)Ir(Me)(H)]$^+$.

NMR Chemical Shift Calculations on [1]$^+$.

Geometries were produced via CH_2 rotation in 15 degree steps around the P3-Ir-C15-H36 torsion (rigid scan, no further optimisation). The P3-Ir-C15-H36 torsion in **[1]$^+$** is 30°.

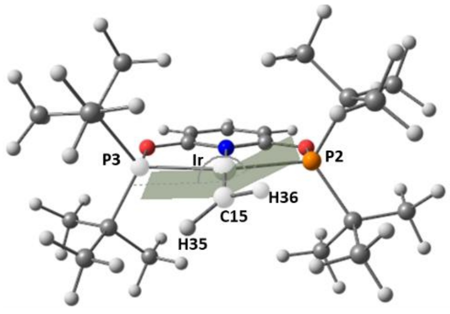

Increment	Relative Energy (kcal/mol)	Δδ C15 (ppm)	Δδ H35 (ppm)	Δδ H36 (ppm)
0	0.00	246.69	14.12	13.94
0*	–	243.68	13.52	13.53
15	0.23	249.00	13.05	12.97
30	0.98	252.24	12.73	12.82
45	2.08	253.05	13.27	13.56
60	3.04	254.91	14.62	15.01
75	3.25	263.37	16.45	16.82
90	2.76	281.76	18.47	18.75
105	2.06	304.91	20.19	20.40
120	1.43	312.70	20.71	20.87
135	0.92	294.98	19.60	19.69
150	0.51	269.49	17.66	17.65
165	0.18	252.38	15.71	15.64
180	0.04	246.70	14.09	13.99

*Figure S43. Computed chemical shifts for the CH_2 group as a function of the P3-Ir-C15-H36 torsion, with relative SCF energies. *corresponds to a calculation including the nearest neighbour anion as an ion-pair.*

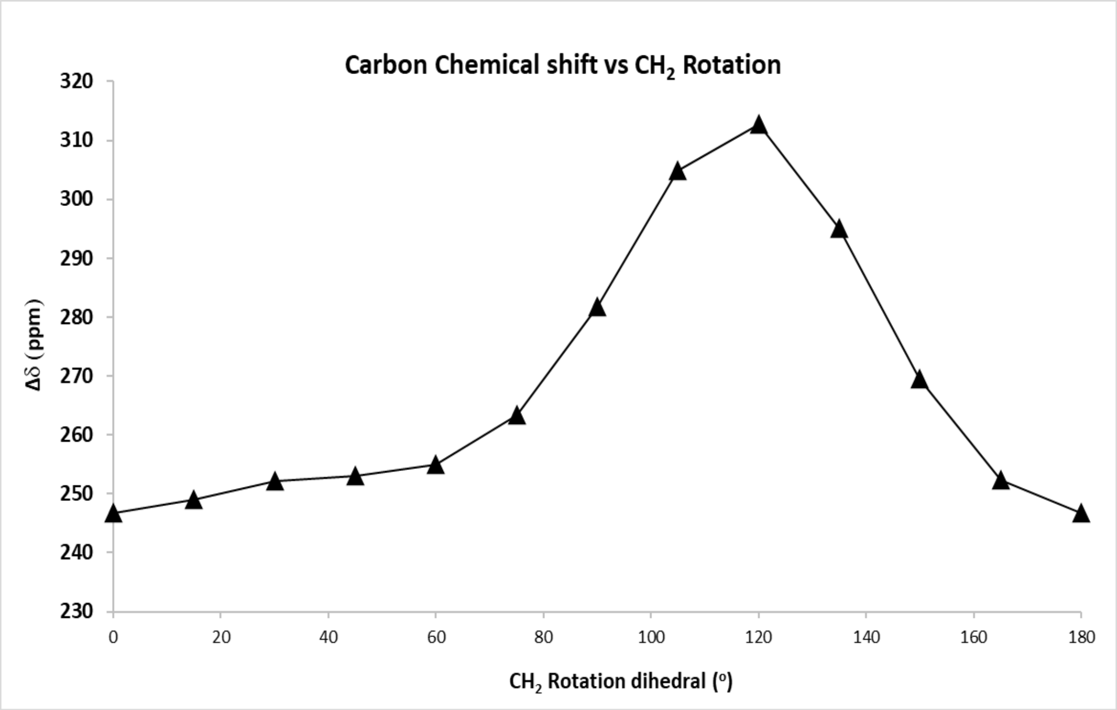

Figure S44. Plot of ^{13}C chemical shift (ppm) of C15 against P3-Ir-C15-H36 torsion angle.

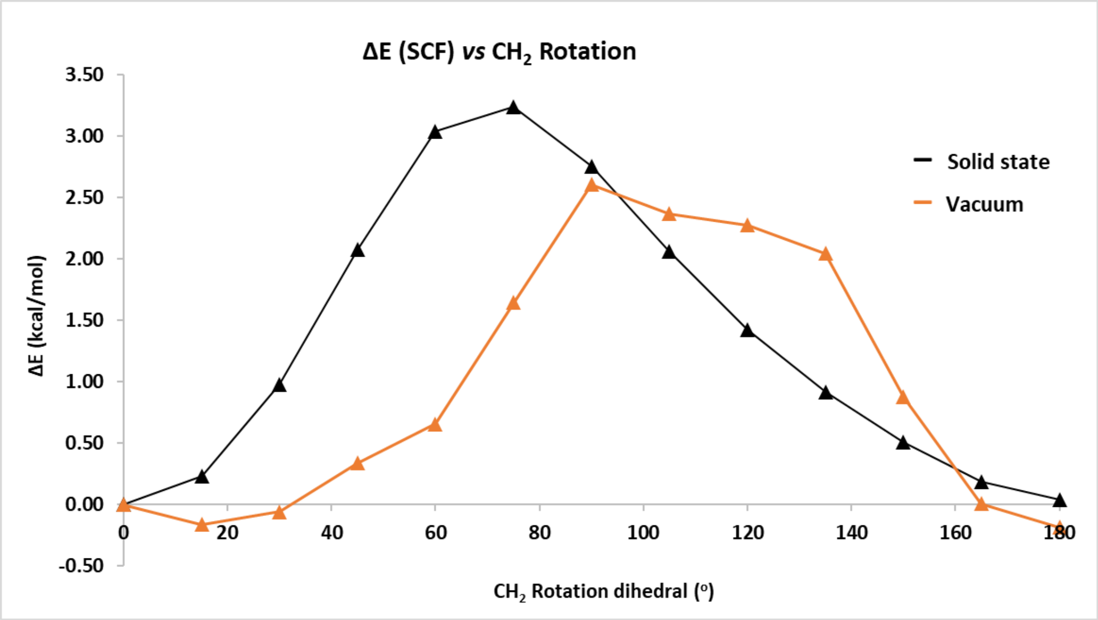

Figure S45. Plot of relative SCF energies (kcal/mol) against P3-Ir-C15-H36 torsion angle computed in the solid state on ***[1][BAr^F$_4$]*** *(black) and the isolated cation (orange).*

[1][BAr$^{F}_{4}$] Ion-pair Interactions

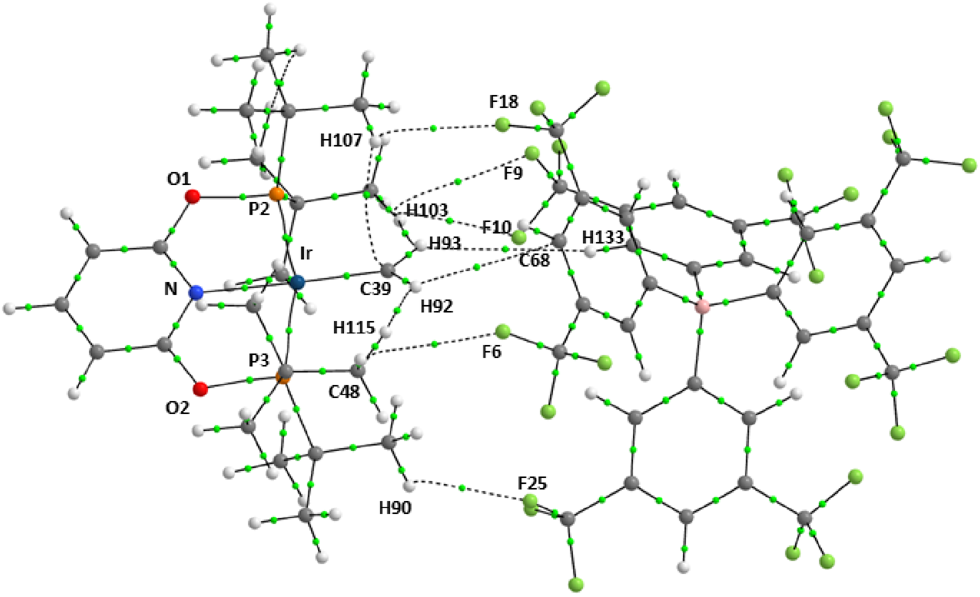

BCP	Distance	ρ	ε	$\nabla^2\rho$	H	DI
H92···C68	3.600	0.0014	0.2689	+0.0041	+0.0003	0.0058
H93···H133	3.603	0.0004	0.1616	+0.0013	+0.0001	0.0011
H107···F18	2.703	0.0048	0.3574	+0.0232	+0.0013	0.0141
H90···F25	2.551	0.0073	0.3473	+0.0330	+0.0016	0.0211
C48···F6	3.269	0.0040	1.0708	+0.0192	+0.0012	0.0154
H103···F10	2.767	0.0050	1.3710	+0.0239	+0.0015	0.0123
H103···F9	3.048	0.0028	2.1930	+0.0142	+0.0010	0.0054

*Figure S46. QTAIM molecular graph showing ion-pair (IP) interactions between **[1]**$^{+}$ and the neighbouring [BAr$^{F}_{4}$]$^{-}$ anion. The IP was extracted from the fully optimised periodic DFT structure. Selected QTAIM BCP parameters (au) and distances (Å) are tabulated.*

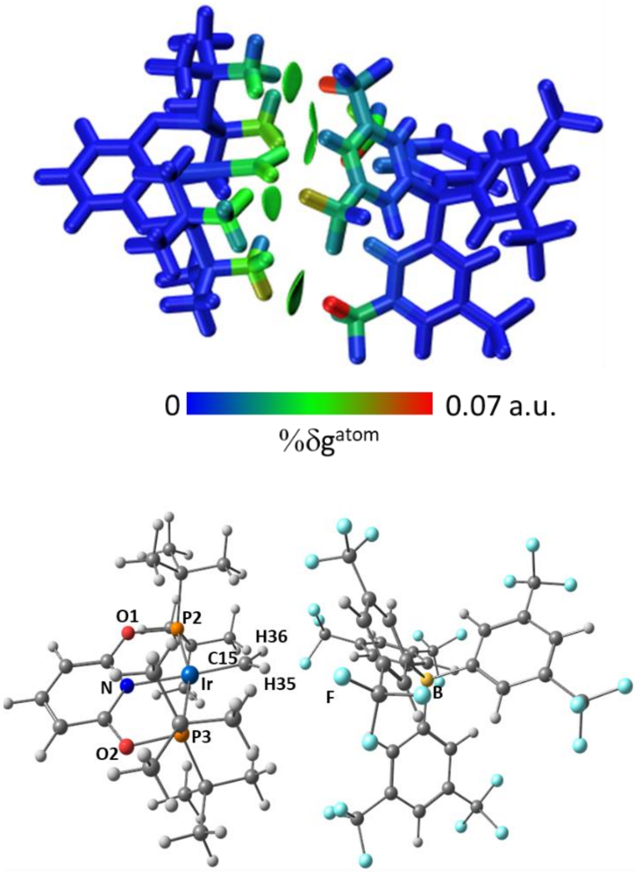

*Figure S47. IGMH plot showing ion-pair (IP) interactions between **[1]**$^+$ and the neighbouring [BAr$^F{}_4$]$^-$ anion. The IP was extracted from the fully optimised periodic DFT structure. Cation and anion are defined as separate fragments; sign(λ_2)ρ–coloured isosurfaces are plotted with δG^{inter} = 0.003 a.u; relative atomic contributions coloured by %δG^{atom}. The molecular structure with selected atom labelling is also shown.*

Reaction of $[1]^+$ with NH_3 (vacuum)

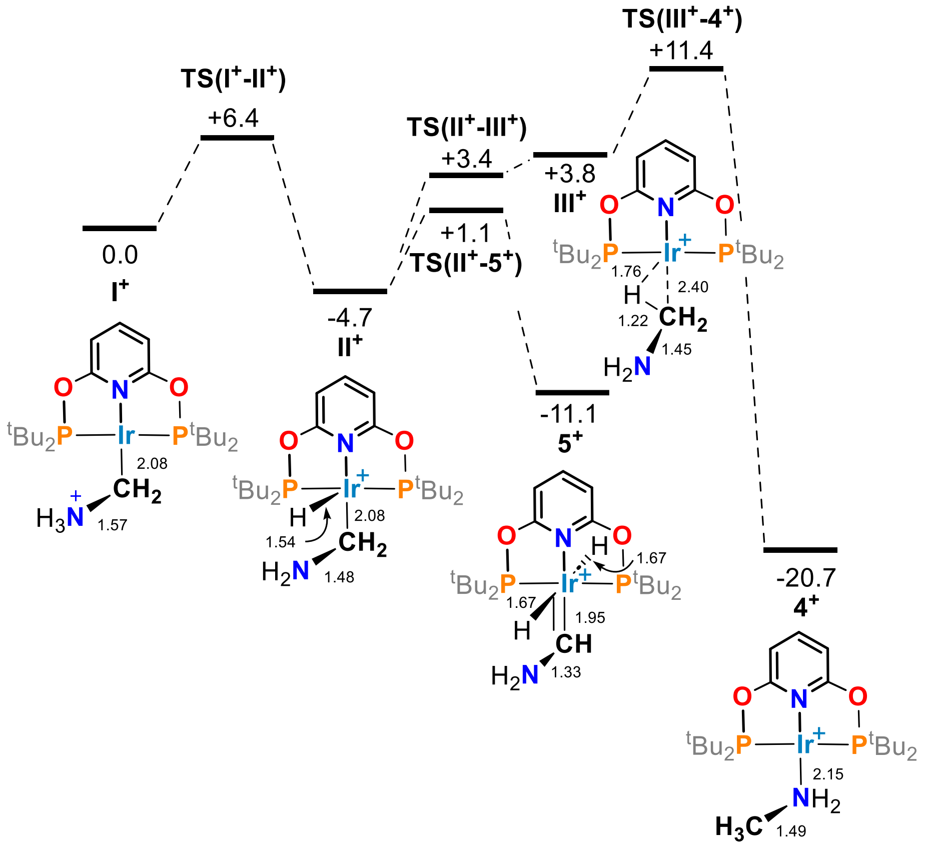

Figure S48. Computed free energy reaction profile (kcal/mol; isolated cation model based on $[1]^+$) for the rearrangement of methylamine ylid, I^+, to methylamine adduct $[4]^+$. Selected distances around the Ir-CH_2NH_3 moiety (Å) are also provided.

Reaction of [1]$^{+}$ with NH_3 (in fluorobenzene solvent)

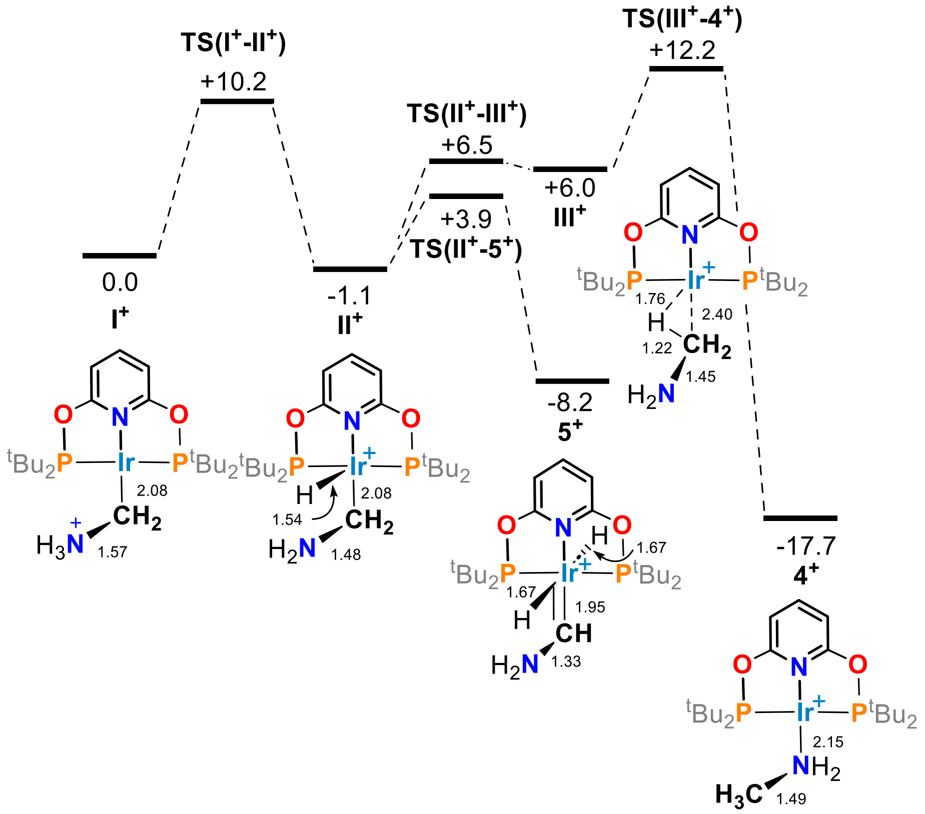

Figure S49. Computed free energy reaction profile (kcal/mol; isolated cation model based on [1]$^{+}$ including a correction for fluorobenzene solvent) for the rearrangement of methylamine ylid, I^{+}, to methylamine adduct [4]$^{+}$. Selected distances around the Ir-CH_2NH_3 moiety (Å) are also provided.

Reaction of [1][BAr$^F_4$] with NH_3 (solid-state, based on [4][BAr$^F_4$])

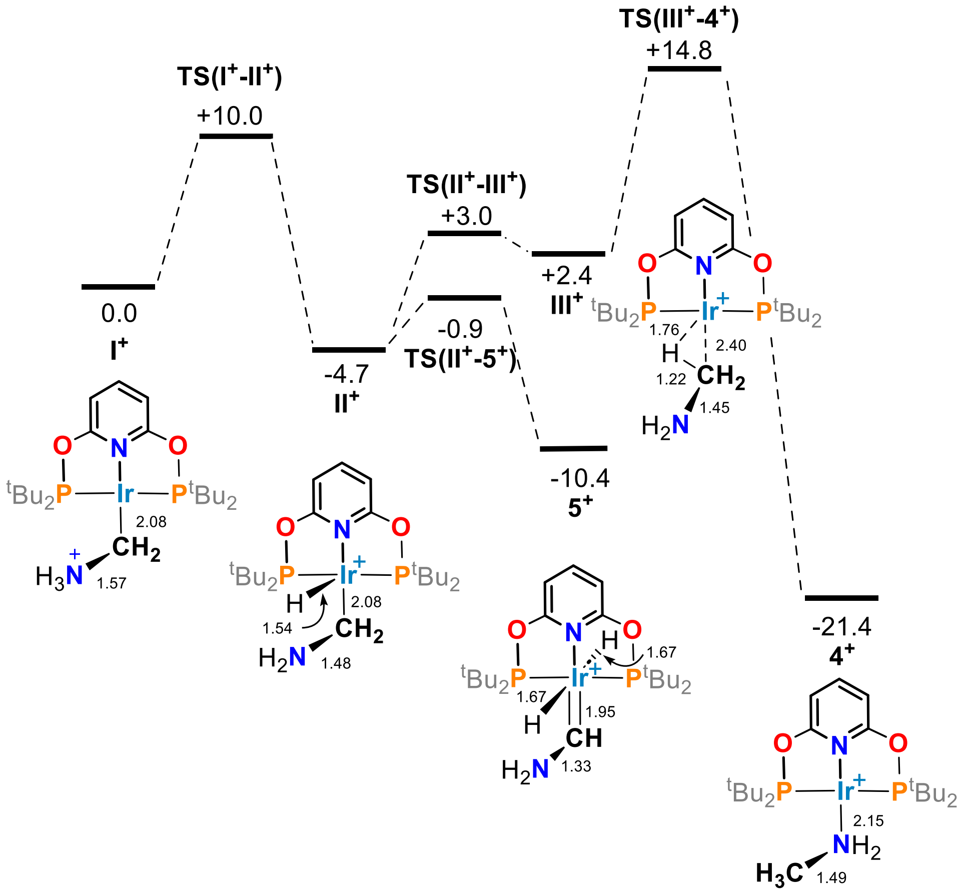

Figure S50. Computed free energy reaction profile (kcal/mol; periodic DFT based on [4][BAr$^F_4$]) for the rearrangement of methylamine ylid, I$^+$, to methylamine adduct [4]$^+$. Selected distances around the Ir-CH_2NH_3 moiety (Å) are also provided.

Reaction of [1][BAr$^F_4$] with NH_3 (solid-state, based on [1][BAr$^F_4$])

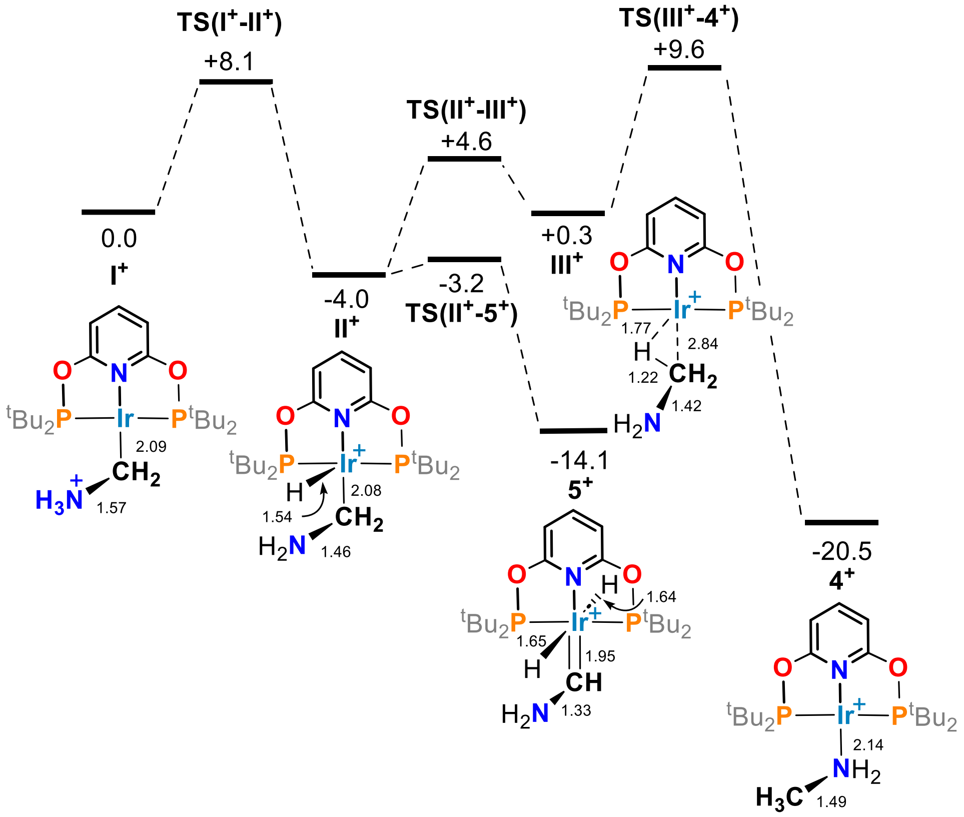

Figure S51. Computed free energy reaction profile (kcal/mol; periodic DFT based on [1][BAr$^F_4$]) for the rearrangement of methylamine ylid, I$^+$, to methylamine adduct [4]$^+$. Selected distances around the Ir-CH_2NH_3 moiety (Å) are also provided. This is the same as Figure 4 in the main text but is included here for ease of comparison with Figure S50.

Table S1. Absolute (au) and relative electronic energies (E_{SCF}) and relative free energies (G, kcal/mol) for stationary points along the rearrangement of rearrangement of methylamine ylid, I⁺, to methylamine adduct [4]⁺.

Stationary Point	Absolute E_{SCF}	Relative E_{SCF}	[a]Relative G^{FVHA}	[b]Relative G^{PVHA1}	[c]Relative G^{PVHA2}	[d]Relative G^{PVHA3}
**I^+**	-8769.09168	0.0	0.0	0.0	0.0	0.0
**TS(I^+-II^+)**	-8769.06874	+14.4	−2.3	9.9	10.0	10.0
**II^+**	-8769.09510	−2.1	−7.4	-4.6	−4.6	−4.7
**TS(II^+-5^+)**	-8769.08587	+3.7	−4.5	−0.8	−0.8	−0.9
**5^+**	-8769.10361	−7.5	−13.9	−10.3	−10.4	−10.4
**TS(II^+-III^+)**	-8769.08146	+6.4	0.2	3.1	3.1	3.0
**III^+**	-8769.08450	+4.5	−0.4	2.5	2.5	2.5
**TS(III^+-4^+)**	-8769.06412	+17.3	11.3	14.9	14.9	14.8
**4^+**	-8769.12622	−21.7	−20.7	−21.3	−21.3	−21.4

[a]Relative free energies based on a full vibrational Hessian analysis on a full unit cell phonon calculation; [b]based on a vibrational Hessian analysis taking into account the reacting cation only based on a full unit cell phonon calculation; [c]based on a partial vibrational Hessian analysis on a full unit cell phonon calculation for the reactant, **I⁺**, and a vibrational Hessian analysis based on a partial phonon calculation taking into account the reacting cation only for all other species. [d]based on a vibrational Hessian analysis based on a partial phonon calculation taking into account the reacting cation only.

Crystallographic data

Single-crystal X-Ray diffraction data were collected on a Rigaku SuperNova diffractometerwith Cu-Kα (λ = 1.54184 Å) radiation equipped with a nitrogen gas Oxford Cryosystems Cryostream unit[S37] at the University of York. Diffraction images from raw frame data were reduced using the CrysAlisPro suite of programmes. The structures were solved using SHELXT[S38] and refined by full convergence on F^2 against all independent reflections by full-matrix least-squares using SHELXL[S39](version 2018/3) through the Olex2 GUI.[S40] All nonhydrogen atoms were refined anisotropically and hydrogen atoms were geometrically placed and allowed to ride on their parent atoms. Disorder of the -CF_3 groups on the $[BAr^F{}_4]^-$ anions were treated by introducing a splitsite model and restraining the geometries and displacement parameters. Distances and angles were calculated using the full covariance matrix. Crystallographic data are available free of charge via the Cambridge Crystallographic Data Centre, under deposition numbers 2342131-2342134.

[1][$BAr^F{}_4$]

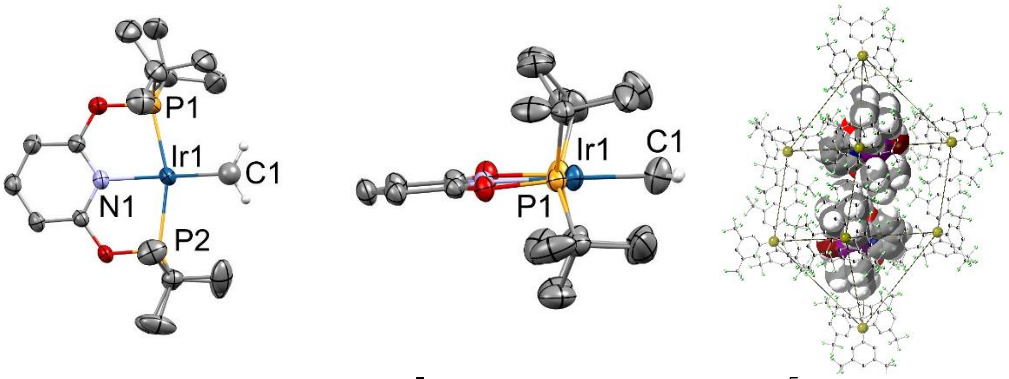

*Figure S36. Solid-state molecular structure of **[1][$BAr^F{}_4$]** and packing arrangement [$BAr^F{}_4$] anions around two molecular cations (displacement ellipsoids are set at 50% probability, non-methylidene hydrogens and [$BAr^F{}_4$] anions have been removed for clarity). Selected bond lengths (Å): Ir-N1 2.080(3), Ir1-P1 2.285(1), Ir-P2 2.289(1), Ir-C1 1.930(5). Selected bond angles (°): P1-Ir-P2 161.09(3), P1-Ir1-C1 100.05(3), N1-Ir-P1 80.24(2), N1-Ir1-P2 80.89(3).*

Table S2 *Crystal data and structure refinement for [1][$BArF_4$].*

CCDC#	2342131
Empirical formula	$C_{54}H_{53}BF_{24}IrNO_2P_2$
Formula weight	1468.92
Temperature/K	110.15
Crystal system	monoclinic
Space group	C2/c
a/Å	16.8854(2)
b/Å	18.0834(2)
c/Å	39.7792(4)
α/°	90
β/°	96.5266(10)
γ/°	90
Volume/Å^3	12067.7(2)
Z	8
ρcalcg/cm^3	1.617
μ/mm^{-1}	5.822
F(000)	5824.0
Crystal size/mm^3	0.14 × 0.07 × 0.06
Radiation	CuKα (λ = 1.54184)
2Θ range for data collection/°	7.704 to 153.526
Index ranges	-21 ≤ h ≤ 20, -10 ≤ k ≤ 22, -50 ≤ 1 ≤ 49
Reflections collected	37908
Independent reflections	11979 [R_{int} = 0.0410, R_{sigma} = 0.0372]
Data/restraints/parameters	11979/186/917
Goodness-of-fit on F^2	1.048
Final R indexes [I>=2σ (I)]	R_1 = 0.0471, wR_2 = 0.1158
Final R indexes [all data]	R_1 = 0.0538, wR_2 = 0.1199
Largest diff. peak/hole / e Å-3	1.60/-1.39

[2][BAr$^F_4$]

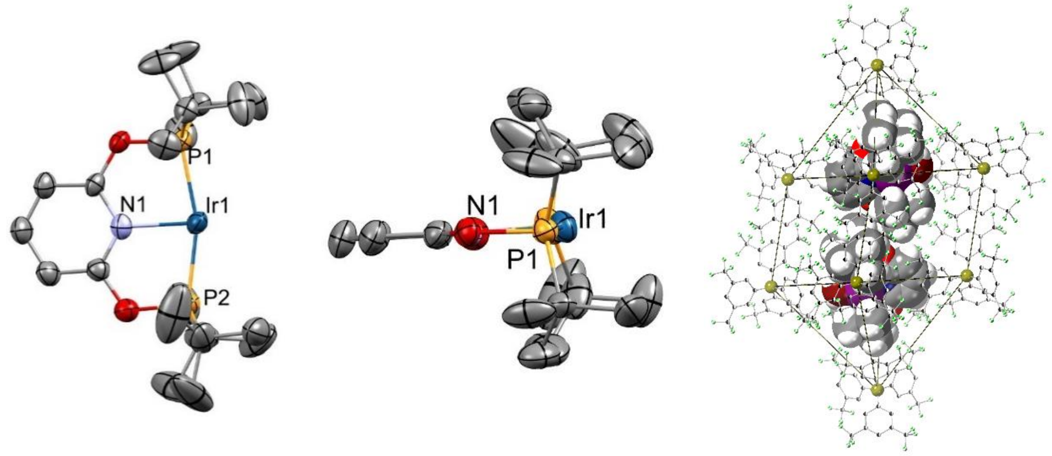

*Figure S50. Solid-state molecular structure of **[2][BAr$^F_4$]** packing arrangement [BAr$^F_4$] anions around two molecular cations (displacement ellipsoids are set at 50% probability, hydrogens and [BAr$^F_4$] anions have been removed for clarity). Selected bond lengths (Å): Ir-N1 2.079(5), Ir1-P1 2.278(2), Ir-P2 2.273(2). Selected bond angles (°): P1-Ir-P2 162.30(6), N1-Ir-p1 81.8(1), N1-Ir1-P2 80.6(1).*

Table S3. *Crystal data and structure refinement for [2][BAr$^F_4$]*	
CCDC#	2342132
Empirical formula	$C_{53}H_{51}BF_{24}IrNO_2P_2$
Formula weight	1454.89
Temperature/K	110.00(10)
Crystal system	monoclinic
Space group	C2/c
a/Å	16.8401(2)
b/Å	18.1311(2)
c/Å	39.9241(5)
α/°	90
β/°	96.5290(10)
γ/°	90
Volume/Å^3	12110.9(2)
Z	8
ρcalcg/cm^3	1.596
μ/mm^{-1}	5.795
F(000)	5760.0
Crystal size/mm^3	0.13 × 0.1 × 0.04
Radiation	Cu Kα (λ = 1.54184)
2Θ range for data collection/°	7.19 to 154.05
Index ranges	-21 ≤ h ≤ 21, -22 ≤ k ≤ 18, -50 ≤ l ≤ 48
Reflections collected	37987
Independent reflections	12342 [R_{int} = 0.0473, R_{sigma} = 0.0457]
Data/restraints/parameters	12342/39/825
Goodness-of-fit on F^2	1.057
Final R indexes [I>=2σ (I)]	R_1 = 0.0627, wR_2 = 0.1553
Final R indexes [all data]	R_1 = 0.0708, wR_2 = 0.1606
Largest diff. peak/hole / e Å^{-3}	2.16/-1.46

[3][BAr$^F_4$]

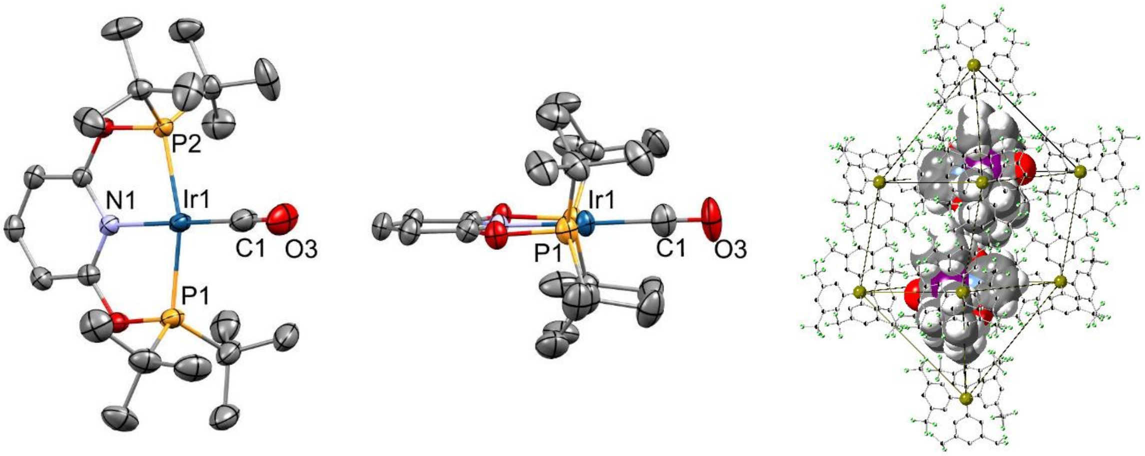

*Figure S51. Solid-state molecular structure of **[3][BAr$^F_4$]** packing arrangement [BAr$^F_4$] anions around two molecular cations (displacement ellipsoids are set at 50% probability, hydrogens and [BAr$^F_4$] anions have been removed for clarity). Selected bond lengths Å: Ir1-C1 1.865(4), Ir1-N1 2.063(3), Ir-P2 2.287(1), Ir-P1 (2.284(1), C1-03 1.129(5). Selected bond angles °: P1-Ir-C1 99.5(1), P2-Ir-C1 99.2(1), Ir1-C1-O3 178.9(4).*

Table S4. *Crystal data and structure refinement for [3][BAr$^F_4$]*	
CCDC#	2342133
Empirical formula	$C_{54}H_{51}BF_{24}IrNO_3P_2$
Formula weight	1482.90
Temperature/K	110.00(10)
Crystal system	monoclinic
Space group	C2/c
a/Å	16.85960(10)
b/Å	18.08390(10)
c/Å	39.6855(2)
α/°	90
β/°	96.2410(10)
γ/°	90
Volume/Å^3	12027.90(12)
Z	8
ρ_{calc}g/cm^3	1.638
μ/mm^{-1}	5.861
F(000)	5872.0
Crystal size/mm^3	0.231 × 0.159 × 0.125
Radiation	Cu Kα (λ = 1.54184)
2Θ range for data collection/°	7.192 to 154.014
Index ranges	-21 ≤ h ≤ 18, -21 ≤ k ≤ 22, -34 ≤ l ≤ 50
Reflections collected	60587
Independent reflections	12505 [R_{int} = 0.0297, R_{sigma} = 0.0219]
Data/restraints/parameters	12505/75/852
Goodness-of-fit on F^2	1.026
Final R indexes [I>=2σ (I)]	R_1 = 0.0347, wR_2 = 0.0888
Final R indexes [all data]	R_1 = 0.0363, wR_2 = 0.0900
Largest diff. peak/hole / e Å^{-3}	1.59/-0.83

[4][BAr$^F_4$]

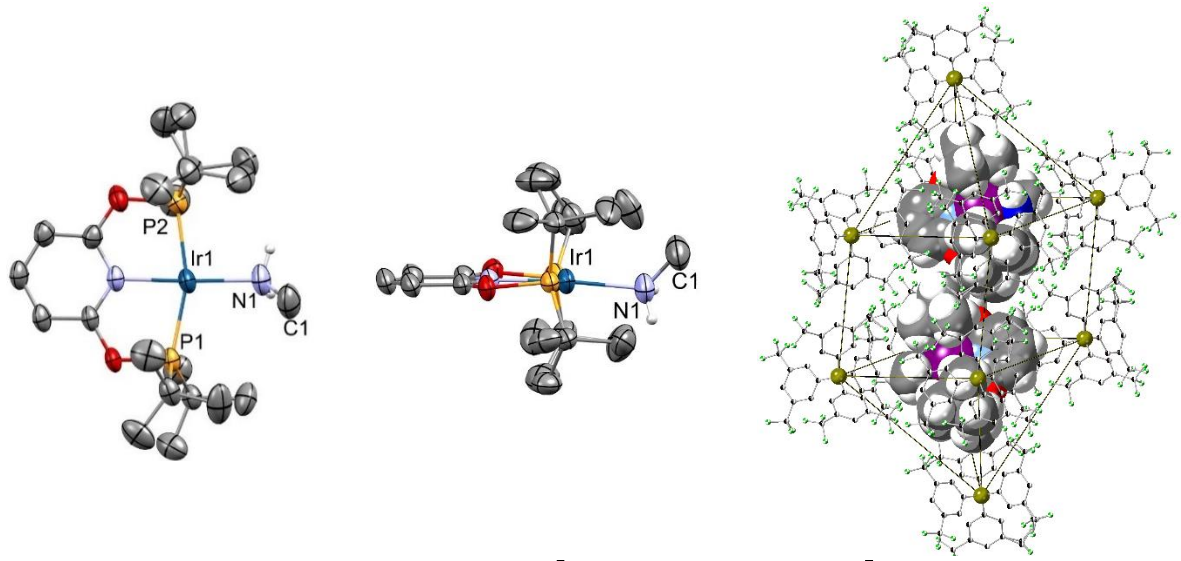

*Figure S37. Solid-state molecular structure of **[4][BAr$^F_4$]** packing arrangement [BAr$^F_4$] anions around two molecular cations (displacement ellipsoids are set at 50% probability, hydrogens and [BAr$^F_4$] anions have been removed for clarity). Selected bond lengths Å: Ir1-N1 2.104(6), Ir1-P1 2.279(2), Ir1-P2 2.262(2), Ir1-N2 2.037(4), N1-C1 1.45(1). Selected bond angles (°): Ir1-N1-C1 122.5.*

***Table S5** Crystal data and structure refinement for [4][BArF$_4$].*	
CCDC#	2342134
Empirical formula	$C_{54}H_{56}BF_{24}IrN_2O_2P_2$
Formula weight	1485.95
Temperature/K	110.15
Crystal system	monoclinic
Space group	C2/c
a/Å	16.93502(9)
b/Å	18.05737(10)
c/Å	40.0220(3)
α/°	90
β/°	96.7065(6)
γ/°	90
Volume/Å^3	12155.06(13)
Z	8
ρcalcg/cm^3	1.624
μ/mm^{-1}	5.792
F(000)	5904.0
Crystal size/mm^3	0.14 × 0.13 × 0.11
Radiation	CuKα (λ = 1.54184)
2Θ range for data collection/°	7.182 to 154.094
Index ranges	-21 ≤ h ≤ 21, -21 ≤ k ≤ 22, -50 ≤ l ≤ 33
Reflections collected	60899
Independent reflections	12463 [Rint = 0.0406, Rsigma = 0.0267]
Data/restraints/parameters	12463/76/881
Goodness-of-fit on F^2	1.023
Final R indexes [I>=2σ (I)]	R_1 = 0.0596, wR_2 = 0.1660
Final R indexes [all data]	R_1 = 0.0624, wR_2 = 0.1688
Largest diff. peak/hole / e Å^{-3}	2.62/-1.34

References

S1. Bernskoetter, W. H.; Hanson, S. K.; Buzak, S. K.; Davis, Z.; White, P. S.; Swartz, R.; Goldberg, K. I.; Brookhart, M. J Am Chem Soc 2009, 131 (24), 8603-8613.

S2. Gu, Z.; Comito, R. J. Organometallics 2022, 41 (15), 1911-1916.

S3. VandeVondele, J.; Krack, M.; Mohamed, F.; Parrinello, M.; Chassaing, T.; Hutter, J. Quickstep: Fast and accurate density functional calculations using a mixed Gaussian and plane waves approach. *Comput. Phys. Commun.* **2005**, *167*, 103-128.

S4. Hutter, J.; Iannuzzi, M.; Schiffmann, F.; VandeVondele, J. cp2k: atomistic simulations of condensed matter systems. *Wires Comput. Mol. Sci.* **2014**, *4*, 15-25.

S5. VandeVondele, J.; Hutter, J. Gaussian basis sets for accurate calculations on molecular systems in gas and condensed phases. *J. Chem. Phys.* **2007**, *127*,

S6. Hartwigsen, C.; Goedecker, S.; Hutter, J. Relativistic separable dual-space Gaussian pseudopotentials from H to Rn. *Phys. Rev. B* **1998**, *58*, 3641-3662.

S7. Goedecker, S.; Teter, M.; Hutter, J. Separable dual-space Gaussian pseudopotentials. *Phys. Rev. B* **1996**, *54*, 1703-1710.

S8. Krack, M. Pseudopotentials for H to Kr optimized for gradient-corrected exchange-correlation functionals. *Theor. Chem. Acc.* **2005**, *114*, 145-152.

S9. Perdew, J. P.; Burke, K.; Ernzerhof, M. Generalized Gradient Approximation Made Simple. *Phys. Rev. Lett.* **1996**, *77*, 3865-3868.

S10. Grimme, S.; Antony, J.; Ehrlich, S.; Krieg, H. A consistent and accurate ab initio parametrization of density functional dispersion correction (DFT-D) for the 94 elements H-Pu. *J. Chem. Phys.* **2010**, *132*, 154104.

S11. Henkelman, G.; Jónsson, H. A dimer method for finding saddle points on high dimensional potential surfaces using only first derivatives. *J. Chem. Phys.* **1999**, *111*, 7010-7022.

S12. Chadwick, F. M.; Krämer, T.; Gutmann, T.; Rees, N. H.; Thompson, A. L.; Edwards, A. J.; Buntkowsky, G.; Macgregor, S. A.; Weller, A. S. Selective C–H Activation at a Molecular Rhodium Sigma-Alkane Complex by Solid/Gas Single-Crystal to Single-Crystal H/D Exchange. *J. Am. Chem. Soc.* **2016**, *138*, 13369-13378.

S13. Ghysels, A.; Verstraelen, T.; Hemelsoet, K.; Waroquier, M.; Van Speybroeck, V. TAMkin: A Versatile Package for Vibrational Analysis and Chemical Kinetics. *J. Chem. Inf. Model.* **2010**, *50*, 1736-1750

S14. Ghysels, A.; Van Speybroeck, V.; Pauwels, E.; Catak, S.; Brooks, B. R.; Van Neck, D.; Waroquier, M. Comparative study of various normal mode analysis techniques based on partial Hessians. *J. Comput. Chem.* **2010**, *31*, 994-1007.

S15. *Gaussian 16 Rev. C.01*; Frisch, M. J.; Trucks, G. W.; Schlegel, H. B.; Scuseria, G. E.; Robb, M. A.; Cheeseman, J. R.; Scalmani, G.; Barone, V.; Petersson, G. A.; Nakatsuji, H.; et al.; Wallingford, CT, 2016.

S16. Grimme, S.; Antony, J.; Ehrlich, S.; Krieg, H. A consistent and accurate ab initio parametrization of density functional dispersion correction (DFT-D) for the 94 elements H-Pu. *J. Chem. Phys.* **2010**, *132*,

S17. Andrae, D.; Häußermann, U.; Dolg, M.; Stoll, H.; Preuß, H. Energy-adjusted ab initio pseudopotentials for the second and third row transition elements. *Theor. Chim. Acta* **1990**, *77*, 123-141.

S18. Höllwarth, A.; Böhme, M.; Dapprich, S.; Ehlers, A. W.; Gobbi, A.; Jonas, V.; Köhler, K. F.; Stegmann, R.; Veldkamp, A.; Frenking, G. A set of d-polarization functions for pseudo-potential basis sets of the main group elements Al-Bi and f-type polarization functions for Zn, Cd, Hg. *Chem. Phys. Lett.* **1993**, *208*, 237-240.

S19. Hehre, W. J.; Ditchfield, R.; Pople, J. A. Self—Consistent Molecular Orbital Methods. XII. Further Extensions of Gaussian—Type Basis Sets for Use in Molecular Orbital Studies of Organic Molecules. *J. Chem. Phys.* **1972**, *56*, 2257-2261.

S20. Hariharan, P. C.; Pople, J. A. The influence of polarization functions on molecular orbital hydrogenation energies. *Theor. Chim. Acta* **1973**, *28*, 213-222.

S21. Tomasi, J.; Mennucci, B.; Cammi, R. Quantum mechanical continuum solvation models. *Chem Rev.* **2005**, *105 (8), 2999-3093*.

S22. Scalmani, G.; Frisch, M. J. Continuous surface charge polarizable continuum models of solvation. I. General formalism. J Chem Phys. ***2010**, 132 (11), 114110*.

S23. Lu, T.; Chen, F. Multiwfn: A multifunctional wavefunction analyzer. *J. Comput. Chem.* **2012**, *33*, 580-592.

S24. Lu, T.; Chen, Q. Independent gradient model based on Hirshfeld partition: A new method for visual study of interactions in chemical systems. *J. Comput. Chem.* **2022**, *43*, 539-555.

S25. Humphrey, W.; Dalke, A.; Schulten, K. VMD: Visual molecular dynamics. *Journal of Molecular Graphics* **1996**, *14*, 33-38.

S26. Bader, R. F. W. *Atoms in Molecules: A Quantum Theory*; Clarendon Press, 1994.

S27. *AIMAll (Version 17.11.14)*; Keith, T. A.; TK Gristmill Software: Overland Park KS, USA, 2017.

S28. Johnson, E. R.; Keinan, S.; Mori-Sánchez, P.; Contreras-García, J.; Cohen, A. J.; Yang, W. Revealing Noncovalent Interactions. *J. Am. Chem. Soc.* **2010**, *132*, 6498-6506.

S29. Contreras-García, J.; Johnson, E. R.; Keinan, S.; Chaudret, R.; Piquemal, J.-P.; Beratan, D. N.; Yang, W. NCIPLOT: A Program for Plotting Noncovalent Interaction Regions. *J. Chem. Theory Comput.* **2011**, *7*, 625-632.

S30. *NBO 6.0*; Glendening, E. D.; Badenhoop, J. K.; Reed, A. E.; Carpenter, J. E.; Bohmann, J. A.; Morales, C. M.; Landis, C. R.; Weinhold, F.; Theoretical Chemistry Institute, University of Wisconsin, Madison, WI, 2013.

S31. *GaussView* Version 6.0; Dennington, R.; Keith, T. A.; Millam, J. M.; Semichem Inc.: Shawnee Mission, KS, 2016.

S32. te Velde, G.; Bickelhaupt, F. M.; Baerends, E. J.; Fonseca Guerra, C.; van Gisbergen, S. J. A.; Snijders, J. G.; Ziegler, T. Chemistry with ADF. *J. Comput. Chem.* **2001**, *22*, 931-967.

S33. *ADF 2020.1*; E.J. Baerends, T. Z., A.J. Atkins, J. Autschbach, O. Baseggio, D. Bashford, A. Bérces, F.M. Bickelhaupt, C. Bo, P.M. Boerrigter, C. Cappelli, L. Cavallo, C. Daul, D.P. Chong, D.V. Chulhai, L. Deng, R.M. Dickson, J.M. Dieterich, F. Egidi, D.E. Ellis, M. van Faassen, L. Fan, T.H. Fischer, A. Förster, C. Fonseca Guerra, M. Franchini, A. Ghysels, A. Giammona, S.J.A. van Gisbergen, A. Goez, A.W. Götz, J.A. Groeneveld, O.V. Gritsenko, M. Grüning, S. Gusarov, F.E. Harris, P. van den Hoek, Z. Hu, C.R. Jacob, H. Jacobsen, L. Jensen, L. Joubert, J.W. Kaminski, G. van Kessel, C. König, F. Kootstra, A. Kovalenko, M.V. Krykunov, P. Lafiosca, E. van Lenthe, D.A. McCormack, M. Medves, A. Michalak, M. Mitoraj, S.M. Morton, J. Neugebauer, V.P. Nicu, L. Noodleman, V.P. Osinga, S. Patchkovskii, M. Pavanello, C.A. Peeples, P.H.T. Philipsen, D. Post, C.C. Pye, H. Ramanantoanina, P. Ramos, W. Ravenek, M. Reimann, J.I. Rodríguez, P. Ros, R. Rüger, P.R.T. Schipper, D. Schlüns, H. van Schoot, G. Schreckenbach, J.S. Seldenthuis, M. Seth, J.G. Snijders, M. Solà, M. Stener, M. Swart, D. Swerhone, V. Tognetti, G. te Velde, P. Vernooijs, L. Versluis, L. Visscher, O. Visser, F. Wang, T.A. Wesolowski, E.M. van Wezenbeek, G. Wiesenekker, S.K. Wolff, T.K. Woo, A.L. Yakovlev; SCM, Theoretical Chemistry, Vrije Universiteit, Amsterdam The Netherlands, 2020.

S34. Becke, A. D. Density-functional thermochemistry. III. The role of exact exchange. *J. Chem. Phys.* **1993**, *98*, 5648-5652.

S35. van Lenthe, E.; Baerends, E. J. Optimized Slater-type basis sets for the elements 1–118. *J. Comput. Chem.* **2003**, *24*, 1142-1156.

S36. van Lenthe, E.; Ehlers, A.; Baerends, E.-J. Geometry optimizations in the zero order regular approximation for relativistic effects. *J. Chem. Phys.* **1999**, *110*, 8943-8953.

S37. J. Cosier and A. M. Glazer, J. Appl. Crystallogr., 1986, 19, 105-107

S38. Sheldrick, G. M. SHELXT – Integrated Space-Group and Crystal-Structure Determination. Acta Crystallogr. Sect. A Found. Adv. 2015, 71 (1), 3–8.

S39. G. Sheldrick, Acta Crystallogr. A, 2008, 64, 112-122.

S40. O. V. Dolomanov, L. J. Bourhis, R. J. Gildea, J. A. K. Howard and H. Puschmann, J. Appl. Crystallogr., 2009, 42, 339-341.
